# *Hyboptera* Chaudoir, 1872 of the Cryptobatida group of subtribe Agrina: A taxonomic revision with notes on their ways of life (Insecta, Coleoptera, Carabidae, Lebiini)

**DOI:** 10.3897/zookeys.714.15113

**Published:** 2017-11-07

**Authors:** Terry L. Erwin, Shasta C. Henry

**Affiliations:** 1 Hyper-diversity Group, Department of Entomology, MRC-187, National Museum of Natural History, Smithsonian Institution, P.O. Box 37012, Washington D.C. 20013-7012, U.S.A.; 2 School of Land and Food, Faculty of Science, Engineering and Technology, University of Tasmania, Private Bag 78, Hobart, Tasmania, Australia, 7001

**Keywords:** Neotropics, Nearctic, Texas, Embioptera, Psocoptera, rain forest, *Hyboptera* Chaudoir, *Thoasia* Liebke, *Straneotia* Mateu, Neotrópicos, Neártico, Texas, Embioptera, Psocoptera, bosque lluvioso, *Hyboptera* Chaudoir, *Thoasia* Liebke, *Straneotia* Mateu

## Abstract

*Hyboptera* Chaudoir, 1872 of the Cryptobatida group of subtribe Agrina, Lebiini, living in the Neotropics and southernmost Nearctic realms are diagnosed, described, and illustrated, and new species are assigned to two inclusive species groups. Occurrences of species range from Texas, USA, to the state of Santa Caterina in Brazil. Seven new species of *Hyboptera* are described:

*Hyboptera biolat* Erwin & Henry, **sp. n.**; Type locality – Perú, Madre de Dios, Manu Reserved Zone, Río Manu, BIOLAT Biological Station, Pakitza; *Hyboptera vestiverdis* Henry & Erwin, **sp. n.**; Type locality – Perú, Madre de Dios, Manu Reserved Zone, Río Manu, BIOLAT Biological Station, Pakitza; *Hyboptera lucida* Henry & Erwin, **sp. n.**; Type locality – French Guiana, Cayenne, Commune de Roura, Montagne des Chevaux; *Hyboptera scheelea* Erwin & Henry, **sp. n.**; Type locality – Perú, Loreto, Pacaya-Samiria National Reserve, Río Samiria (South Branch), Camp Terry; *Hyboptera shasta* Erwin, **sp. n.**; Type locality – Brazil, Amazonas, north of Manaus on Amazonas 010 at Km 26, Reserva Ducke;

*Hyboptera tepui* Erwin & Henry, **sp. n.**; Type locality – Venezuela, Amazonas, Cerro de la Neblina, Río Baria Basecamp; *Hyboptera tiputini* Erwin & Henry, **sp. n.**; Type locality – Ecuador, Orellana, Yasuni National Park (edge), 95.43 km E (heading 101.46°) Coca, Tiputini Biodiversity Station. A revised identification key is provided to the genera of the Cryptobatida group and another to the species of *Hyboptera* Chaudoir and distributional data are provided for all known species of the latter. Adults of these species often occur in the canopy of many tropical tree species and records are reported where known. In addition, adults are found under the webbing of Psocoptera and in fleshy anther rings of Bombacaceae (*Pseudobombax septenatum* (Jacq.) Dugand), on the rain forest floor in the dry season. Larval and pupal stages of one species from Panamá are known from under bark of living fence posts; however, these immature stages are not treated in the current paper.

## Abstract (es)

*Hyboptera* Chaudoir, 1872, del grupo Cryptobatida, perteneciente a la subtribu , Lebiini, viviendo en los Neotrópicos y en las áreas meridionales del Neártico se diagnostican, describen e ilustran y nuevas especies se asignan a dos grupo-especies inclusivos. La distribución de las especies recorre desde Texas, EE.UU., hasta el estado de Santa Caterina en Brasil. Siete nuevas especies de *Hyboptera* se describen:

*Hyboptera tepui* Erwin & Henry, **sp. n.**; Type locality – Venezuela, Amazonas, Cerro de la Neblina, Río Baria Basecamp; *Hyboptera tiputini* Erwin & Henry, **sp. n.**; Type locality – Ecuador, Orellana, Yasuni National Park (edge), 95.43 km E (heading 101.46°) Coca, Tiputini Biodiversity Station. Se provee una clave revisada para los géneros dentro del grupo Cryptobatida y otra para las especies del género *Hyboptera* Chaudoir y se incluye información sobre la distribución de todas las especies conocidas de éste último. Los adultos de estos comúnmente se encuentran en el dosel de muchas especies de árboles tropicales y registros se reportan cuando se conocen. Además, los adultos pueden estar debajo de las telas de sócidos y en los anteridios carnosos de los Bombacaceae (*Pseudobombax septenatum* (Jacq.) Dugand) en el suelo del bosque lluvioso. Las etapas de larva y pupa de una de las especies de Panamá se conocen viviendo debajo de la corteza de postes vivos de cercas; sin embargo, estos so se tratan en este artículo.

## Introduction

The Cryptobatida Group of Lebiini was proposed to include a number of genera including *Hyboptera* Chaudoir (Erwin 2004). This genus was treated most recently by Reichardt (1971, 1973) and those reports were brief and superficial, not full revisions. Before that, only five isolated species descriptions by Chaudoir, Reiche and Oberthür existed. Hence, this small group of very attractive and biologically interesting beetles requires an initial taxonomic revision. The purpose of this paper is to rapidly validate some new species names (Erwin and Johnson 2000) with descriptions and provide more complete re-descriptions of known species, so that the group is available for further study, particularly their way of life in association with Psocoptera. *Hyboptera* are members of the Cryptobatida group by virtue of the structure of their mouthparts, elytral transverse depression, and the male genitalia with a flagellum. Herein, we provide full descriptions of all known taxa, including color images, an up-to-date map of their known distributions, and what is known of their ways of life including host tree species.

## Methods and materials

(modified from Erwin and Zamorano 2014)

As noted in several past contributions, methods and species concepts follow those previously described (Erwin and Kavanaugh 1981; Kavanaugh and Erwin 1991). The species validation and diagnosis format follows as closely as possible that suggested in Erwin and Johnson (2000). For measurements, images of the specimens were taken using a Leica M420 stereoscope coupled to an EntoVision^TM^ system. The resulting image was processed using the software Cartograph version 7.2.5 by Microvision Instruments. The magnification on the zoom was set to calibrate the system and it is embedded into the file of the image. The image was then opened with the software program Archimed version 6.1.4, also by Microvision, and the Measure tool was then used to determine the lengths of the various parts. A total of 239 images were obtained. Measurements of length (ABL, SBL) and width (TW) follow those of Ball (1972) and Kavanaugh (1979): ABL (apparent body length), measured from apex of labrum to apex of longer elytron (in adults of this genus, the abdomen often protrudes beyond the elytral apex, thus the ABL often is much larger that the SBL; SBL (standardized body length), equals the sum of the lengths of the head (measured from apex of clypeus to a point on midline at level of the posterior edge of compound eyes); PL (pronotum length) is measured from apical to basal margin along midline; LE (elytron length) is measured from apex of scutellum to apex of the longer elytron; TW (total width) is measured across both elytra at their widest point with suture closed; and WH (head width) is measured from extreme margin of protuberant eyes left to right. Note that not all specimens available were measured because more than 33 specimens were available, thus we limited “n” to 33 as a statistically valid sample size. Sexes were measured separately, we found slight differences among the species sexes, and hence we report measurements for both sexes in our Tables (see Appendix 1). For the *Hyboptera* treatment below, we provide relative size terms based on the SBL as follows: small-size < 3.3 mm, medium-size 3.3 mm to 4.3 mm, and large-size > 4.3 mm. For an explanation of the measurements and their incorporation in Appendix 1, see Erwin (2011) and Erwin and Ball (2011). For the present study, we report the harmonic mean, as we believe it better reflects the central tendency than the arithmetic mean.

Attributes of the abdominal ventral sterna are referred to using the numbering system generally accepted in carabid studies, i.e., the sternum divided medially by the hind coxae is sternum II (the first being hidden) and the last visible is sternum VII (Liu et al. 2011). In a revision of the genus *Pericompsus* (Erwin 1974), a problem was encountered with the term “stria” for features of their punctate elytra (i.e., the so-called striae were *not* actually striae, rather they were rows of punctures). The result was the use of the term “interneur” to apply to the attribute lying between intervals. Through use of this term, one could describe the feature as interneur striate, punctate, striatopunctate, etc.

A similar problem exists for the proximal end of the median lobe of the male genitalia. In Snodgrass (1935), the term “phallobase” is used, and we have adopted it here (see Erwin 2011). Therefore, by extension, in Carabidae, we can say phallobase hooded (e.g., Lebiini, Pseudomorphini), phallobase of two parallel sclerotized struts (basal trechines and *Andinodontis*), phallobase of two uneven struts (*Bembidion*), etc. Kavanaugh (pers. comm.) points out that with struts there are still connecting membranes surrounding the struts forming a “bulb.” We have chosen the aedeagal illustration of a male *H. angulicollis* Chaudoir (Fig. 5A) and *H. apollonia* Erwin (Fig. 10A) to display the identifying code letters and these apply to all illustrations of male genitalia of *Hyboptera* included.

This study includes 738 adult specimens of *Hyboptera*, all currently at the National Museum of Natural History, Washington, DC (NMNH) and, where appropriate, returned to their corresponding institutions upon publication. Among these specimens, some were received from:

**AMNH** American Museum of Natural History, New York, NY, USA (Lee Herman)

**BMNH** Natural History Museum, London, UK (Beulah Garner)

**CAS** California Academy of Sciences, Berkeley, CA, USA (David H. Kavanaugh)

**CMNH** Carnegie Museum of Natural History, Pittsburgh, PA, USA (Robert L. Davidson)

**HESP** Henry Hespenheide Private Collection, Los Angeles, CA, USA (Henry Hespenheide)

**IAvH** Instituto de Investigación de Recursos Biológicos, Alexander von Humboldt, Bogotá, Colombia (Arturo González)

**INBIO** Instituto Nacional de Biodiversidad, Santo Domingo, Costa Rica (Angel Solis)

**LACM** Los Angeles County Museum, Los Angeles, CA, USA (Brian V. Brown)

**MCZ** Museum of Comparative Zoology, Harvard University, Boston, MA, USA (Philip Perkins)

**MNHP** Muséum national d’Histoire naturelle, Paris, France (Thierry Deuve)

**NBCL** Naturalis Bidiversity Center, Lieden, Netherlands (Luc Willemse)

**OSU** C.A. Triplehorn Insect Collection, Ohio State University, Columbus, OH, USA (Charles A.Triplehorn)

**SEMC** Snow Entomological Museum, University of Kansas, Lawrence, Kansas, USA (Zachary Falin)

**UASM** EH Strickland Entomological Museum, University of Alberta, Edmonton, Canada (George E. Ball & Danny Shpeley)

**UCD** Bohart Museum of Entomology, University of California, Davis, CA, USA (Lynn Kimsey)

**UNMSM** Universidad Nacional Mayor de San Marcos, Lima, Perú (Gerardo Lamas)

Primary type specimens of new species will be deposited in their countries of origin if required by legal agreements, or museums of ownership at the conclusion of our studies on this tribe.

The enhanced habitus images of the adult beetles portray most of the character states referred to in the key provided. Illustrations of male genitalia are standard for descriptive taxonomy of carabid beetles in both preparation and aspects presented, as is the presentation of the female genitalia (one example per genus, in this case *H. lucida* sp. n.). The habitus images of the adults were made with a Visionary Digital^TM^ high resolution imaging system rendered using Photoshop to become “Digital Photo-illustrations.” Figure captions include an ADP number, which is a unique identification number for the specimen that was imaged and links the specimen and associated illustrations and/or images to additional information, such as collecting notes, in electronic databases at the NMNH.

Geographical data are presented for species based on all known specimens available at the time of manuscript preparation, including those in the literature. Geo-referenced data have been determined from locality information provided on specimen labels; only those exact geo-references reported in decimal degrees that are provided on the label are placed in quotes. Otherwise, we have estimated others as closely as possible from places, mileage, or other locality data listed on the label and searched with Google Earth Pro. Latitude and longitude for those are reported in decimal degrees and have been corrected from those reported on the labels, if necessary; our bottom line is that georeferenced locality data reported herein are far more accurate than those provided on specimens labels.

A distribution map is provided for the species of *Hyboptera* (Fig. 11). Here, vernacular names in English are proposed, as common names are becoming increasingly needed in conservation reports and studies, and/or agricultural and forestry applications. These names are based on criteria set forth in Erwin (2011a) and applied in Erwin (2011b).

Host occurrences of rainforest trees are reported using the names provided by botanists who inventoried two fogging transects established by the senior author (TLE) in Ecuador. These names have not been elaborated with author names herein, as is traditional in botanical literature, however, they can be readily found on the internet.

## Accounts of taxa

### Tribe Lebiini, Subtribe Agrina, Cryptobatida Group

#### Diagnosis.

Adults. Head ventrally without suborbital setigerous punctures, neck not markedly narrowed, except somewhat in *Thoasia*. Mandible widened near base, scrobe wide, lateral margin markedly rounded; dentition of occlusal margins reduced, typical for Lebiini (cf. Shpeley and Ball 2001: figs 6–9); palpi with ultimate articles sub-securiform or securiform, paraglossae broad, glabrous, adherent, extended to anterior angle of glossal sclerite. Elytron usually with transverse depression at anterior third, appearing deformed; penultimate setigerous puncture of elytron umbilicate series not displaced laterally nor medially. Posterior tibial spurs subequal, their margins smooth; tarsomere 4 usually bilobed. Except in one genus (i.e., some species of *Aspasiola*), endophallus with flagellum [the absence from males of some species of *Aspasiola* species probably represents a reversal (secondary loss)].

#### Notes.

Subtribe Agrina consists of those species formerly included in the Subtribe Calleidina (cf. Lorenz 2005). The Cryptobatida Group, by virtue of the attributes above, has the type genus *Cryptobatis* Eschscholtz. Many records of species in most genera refer to collections on fungus arrayed on sides or the bottom of fallen tree trunks. It is probable that the Cryptobatida Group contains many species whose adults are predators on other shelf fungus inhabitants (cf. Erwin and Erwin 1976, wherein adults and larvae of a species of *Eurycoleus*, which is a member of the subtribe Pericalina, are predaceous on fungal feeders; other pericalines are also associated with fungi). *Hyboptera* and its adelphotaxon *Hybopteroides* are exceptions (Erwin and Ball 2012), as the adults are likely predators on psocids and embiids beneath silken webs, respectively.

Below, we have added to the Cryptobatida Group the genus *Straneotia* Mateu 1961 by virtue of a study of new specimens collected by fogging and flight intercept traps (FIT) in Ecuador and French Guiana, respectively. Mateu erected this genus based on a single female specimen from eastern Amazonian Brazil (*Straneotia freyi* Mateu) and described a second species in the genus based on a single female from western Amazonian Brazil (*Straneotia amazonica* Mateu). These two species soon will be redescribed and the new species described (Erwin and Aldebron, in prep).

#### Key to the genera of the Cryptobatida Group, Subtribe Agrina (Lebiini)

(Enhanced from Erwin 2004 and Erwin and Ball 2012)

**Table:** 

1	Elytron at basal third depressed, surface uneven, tuberculate or not, and/or margin of pronotum angulate or sub-angulate at mid-lateral setiferous pore, or tubular	**Cryptobatida Group**...**2**
1’	Elytron neither depressed nor tuberculate, surface smooth with normal interneurs and intervals (or interneurs effaced); side of pronotum evenly rounded	**Plochionida Group, Calleidida Group, Agrida Group**
2(1)	Elytron markedly tuberculate overall, or with a series of small setiferous tubercles on intervals 3 and 5, lateral marginal intervals without callus	**3**
2’	Elytron without trace of discal tubercles; lateral marginal intervals with or without callus	**6**
3(2)	Prothorax somewhat tubular, much narrower than head	***Otoglossa* Chaudoir, 1872**
3’	Prothorax wider than head	**4**
4(3’)	Sides of pronotum narrowly reflexed except at mid-lateral seta, there wide; neck well-defined, narrow; elytra metallic blue, fore-body all or mostly rufous	***Thoasia* Liebke, 1939**
4’	Sides of pronotum broad and margins broadly reflexed throughout; neck broad; color otherwise	**5**
5(4’)	Elytron with numerous tubercles on at least three intervals; head dorsum transversely rugose; side margins of pronotum subtly angulate or not; labrum large, broadly bilobed; tarsomere 4 bilobed	***Hyboptera* Chaudoir, 1872**
5’	Elytron not tuberculate; head dorsum longitudinally strigose (wrinkled); side margins of pronotum markedly angulate; labrum normal, rectangulate, slightly emarginate or truncate apically; tarsomere 4 not bilobed	***Hybopteroides* Erwin & Ball, 2012**
6(2’)	Antennomere 4 glabrous except for apical ring setae	**7**
6’	Antennomere 4 multisetiferous from basal third to apex, in addition to apical ring setae	**8**
7(6)	Elytron laterally with callus at apical third; male endophallus without flagellum	***Valeriaaschero* Erwin, 2004**
7’	Elytron laterally without callus at apical third; male endophallus with or without flagellum	***Aspasiola* Chaudoir, 1877**
8 (6’)	Head markedly narrow, elongate, and tubular; eyes more or less flat	***Straneotia* Mateu, 1961**
8’	Head normal with large hemispheric eyes	**9**
9(8’)	Head and pronotum densely and evenly punctate	***Cylindronotum* Putzeys, 1845**
9’	Head and pronotum smooth	**10**
10 (9’)	Pronotum with lateral margin narrowly reflexed from base to apex	***Pseudotoglossa* Mateu, 1961**
10’	Pronotum with lateral margin moderately or markedly reflexed from base to apex	**11**
11(10’)	Elytron laterally at apical third with large callus	***Cryptobatis* Eschscholtz, 1829**
11’	Elytron laterally at apical third without callus	***Onota* Chaudoir, 1872**

### Hyboptera

Taxon classificationAnimaliaColeoptera Carabidae

Chaudoir, 1872

Humps-backed beetles Figs 1 , 2 , 3 , 4 , 5 , 6 , 7 , 8 , 9 , 10 , 11

Hyboptera Chaudoir, 1872: 161. Type species: Hyboptera angulicollis Chaudoir, 1872: 164, designated by Reichardt (1973:50).
Aspasia Reiche, 1842: 310 (not Dejean 1831). Type species: Aspasia verrucosa Reiche, 1842: 310. Synonymized by Chaudoir 1872: 161.

#### Diagnosis.

**Figure 1.**
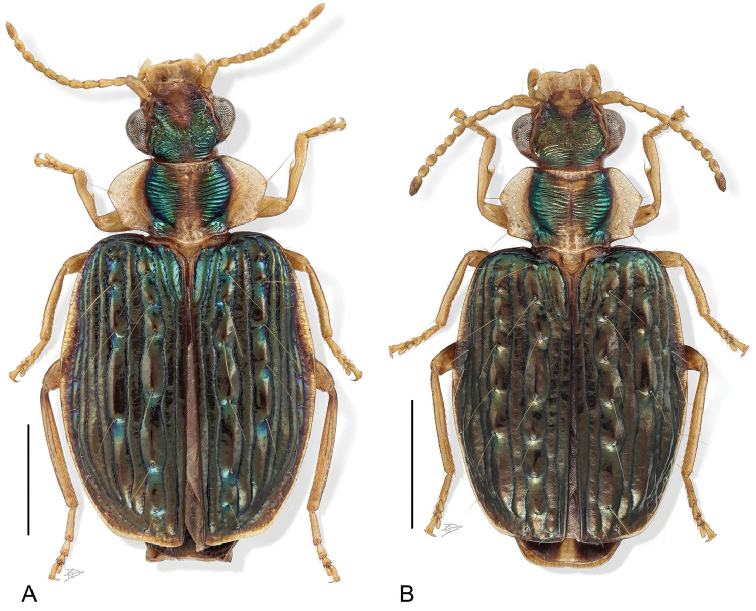
Digital Photo-illustration. Habitus, dorsal aspect. **A** *Hyboptera angulicollis* Chaudoir, female, ADP052729 **B** *Hyboptera biolat* Erwin & Henry, sp. n., female, ADP007398.

**Figure 2.**
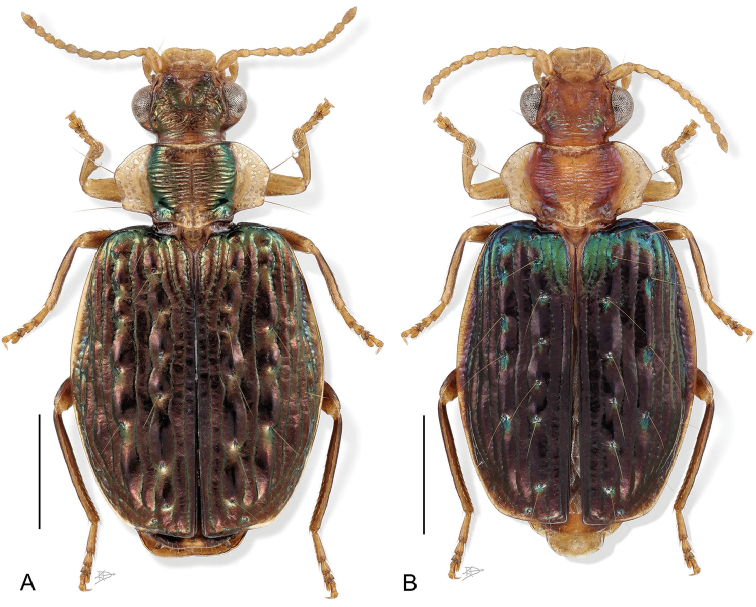
Digital Photo-illustration. Habitus, dorsal aspect. **A** *Hyboptera vestiverdis* Henry & Erwin, sp. n., female, ADP007334 **B** *Hyboptera scheelea* Erwin & Henry, sp. n., female, ADP007575.

**Figure 3.**
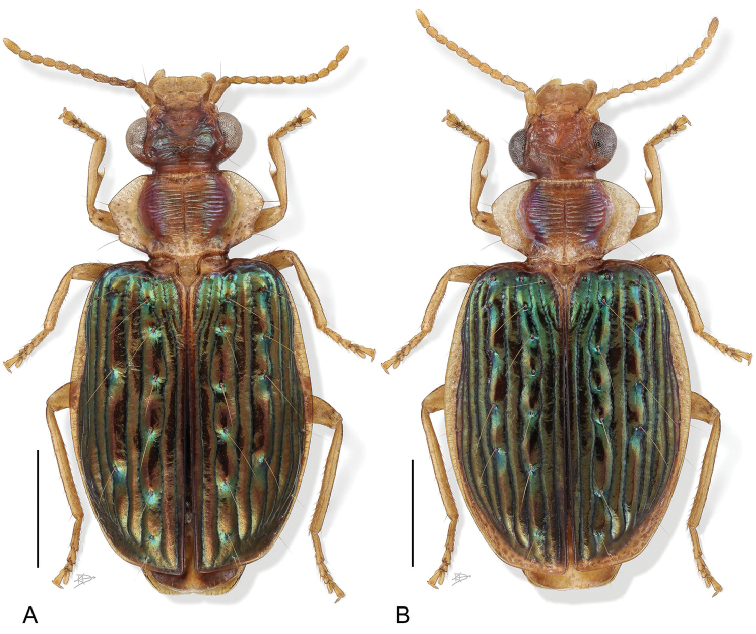
Digital Photo-illustration. Habitus, dorsal aspect. **A** *Hyboptera shasta* Erwin, sp. n., male, ADP135875 **B** *Hyboptera tepui* Erwin & Henry, sp. n., male, ADP007676.

**Figure 4.**
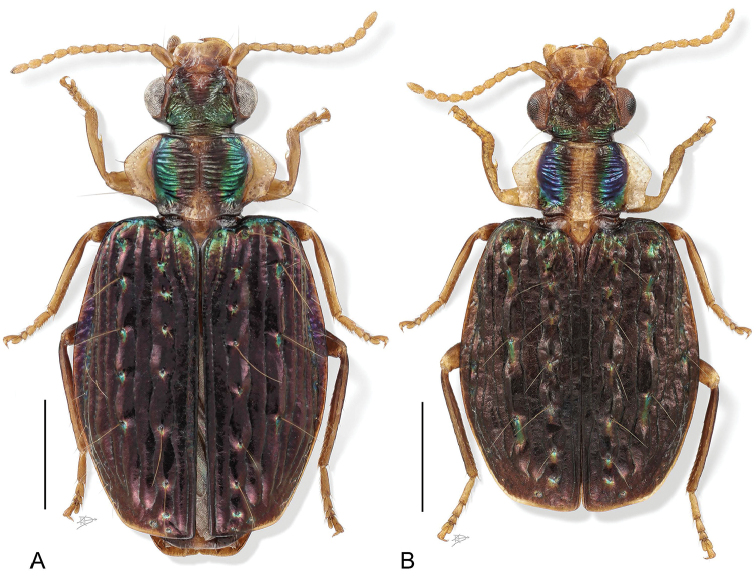
Digital Photo-illustration. Habitus, dorsal aspect. **A** *Hyboptera tiputini* Erwin & Henry, sp. n., male, ADP135850 **B** *Hyboptera viridivittis* Chaudoir, male, ADP007599.

**Figure 5.**
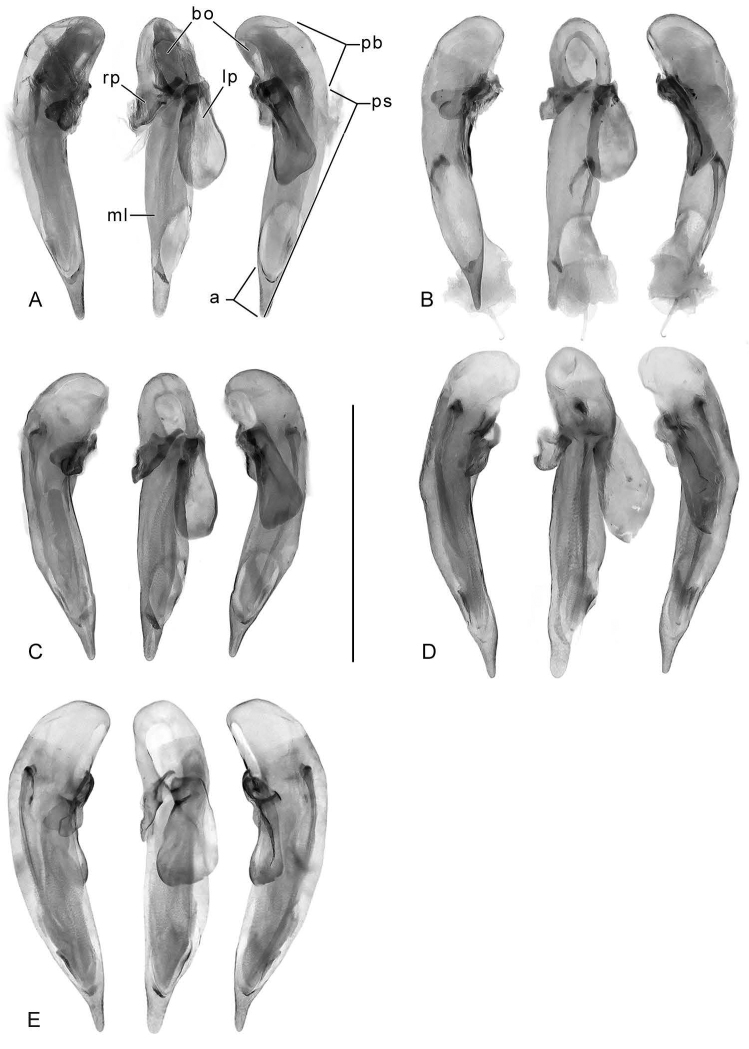
Digital Photo-illustration, male aedeagus in repose, dorsal, ventral, left lateral aspects. **A** *Hyboptera angulicollis* Chaudoir, ADP007330 **B** *Hyboptera biolat* Erwin & Henry, sp. n., ADP007443 **C** *Hyboptera vestiverdis* Henry & Erwin, sp. n., ADP014856 **D** *Hyboptera tiputini* Erwin & Henry, sp. n., ADP135845 **E** *Hyboptera viridivittis* Chaudoir, ADP007601. Legend, lp, left paramere; rp, right paramere; pb, phallobase; bo, phallobase orifice; ps, phalloshaft; a, phalloapex. Scale line = 0.25 mm.

**Figure 6.**
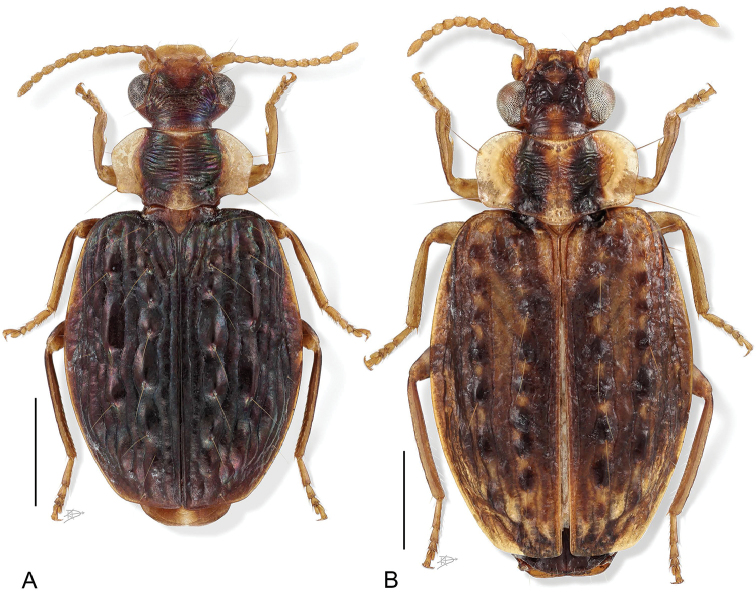
Digital Photo-illustration. Habitus, dorsal aspect. **A** *Hyboptera apollonia* Erwin, male, ADP007944 **B** *Hyboptera auxilidora* Erwin, male, ADP007622.

**Figure 7.**
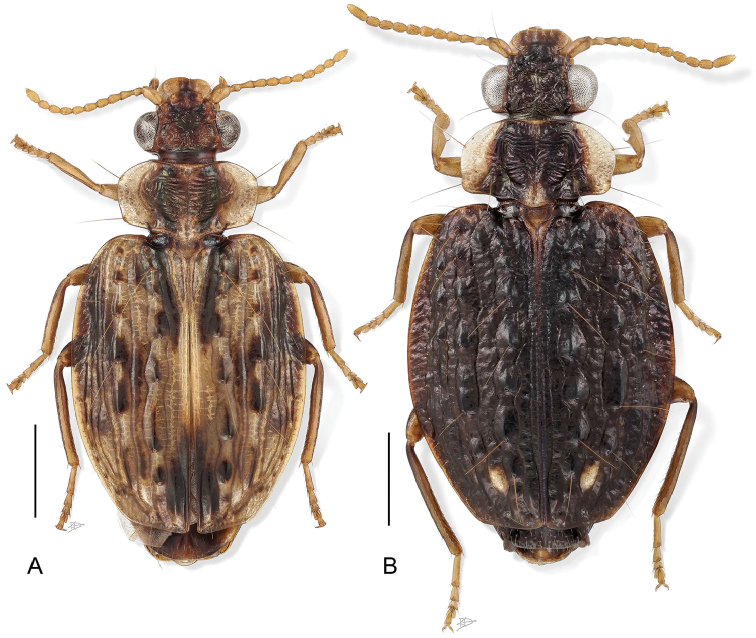
Digital Photo-illustration. Habitus, dorsal aspect. **A** *Hyboptera dilutior* Oberthür, male, ADP094021 **B** *Hyboptera lucida* Henry & Erwin, sp. n., female, ADP135783.

**Figure 8.**
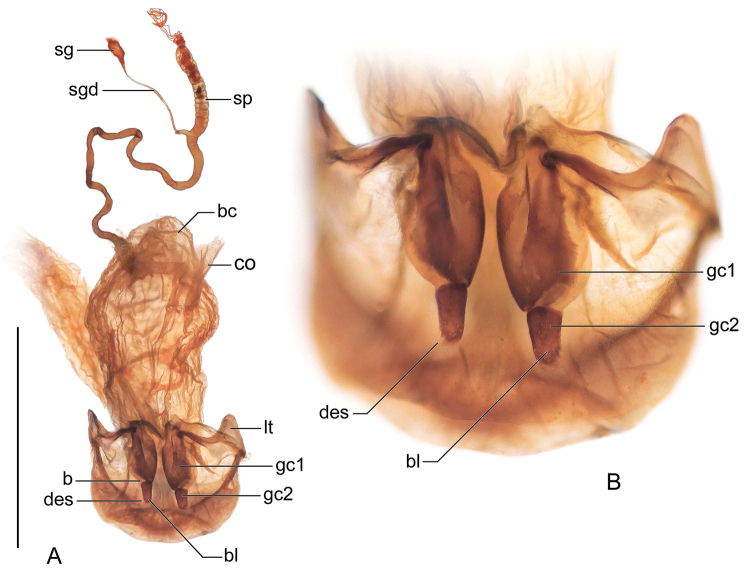
Digital Photo-illustration, female reproductive system dorsal and ventral aspects. *Hyboptera lucida* Henry & Erwin sp. n., ADP148100. Legend, bc, bursa copulatrix; sg, spermathecal gland; sgd, spermathecal gland duct; sp, spermatheca. Dorsal aspect; lt, laterotergite; gc1, gonocoxite 1; gc2, gonocoxite 2, des, dorsal ensiform seta, b, base of gonocoxite 2; bl, blade of gonocoxite 2. Scale line = 0.25 mm.

**Figure 9.**
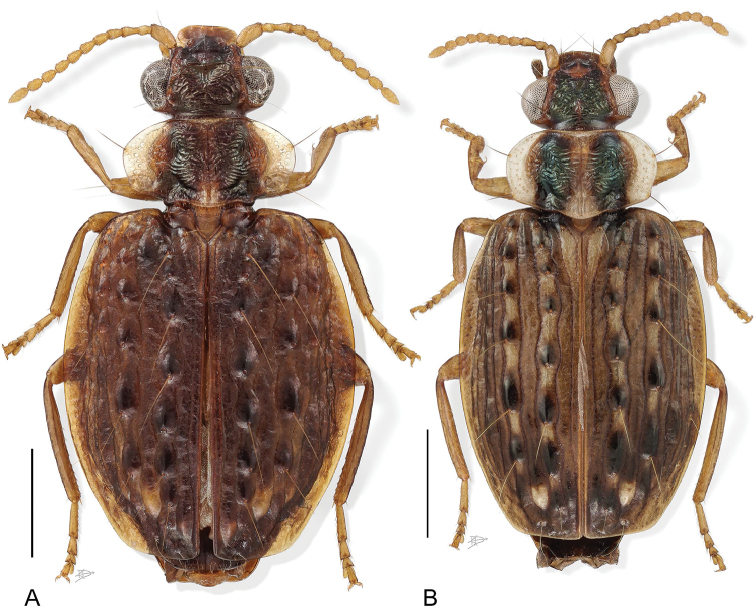
Digital Photo-illustration. Habitus, dorsal aspect. **A** *Hyboptera tuberculata* (Dejean), female, ADP007533 **B** *Hyboptera verrucosa* (Reiche), female, ADP086946.

**Figure 10.**
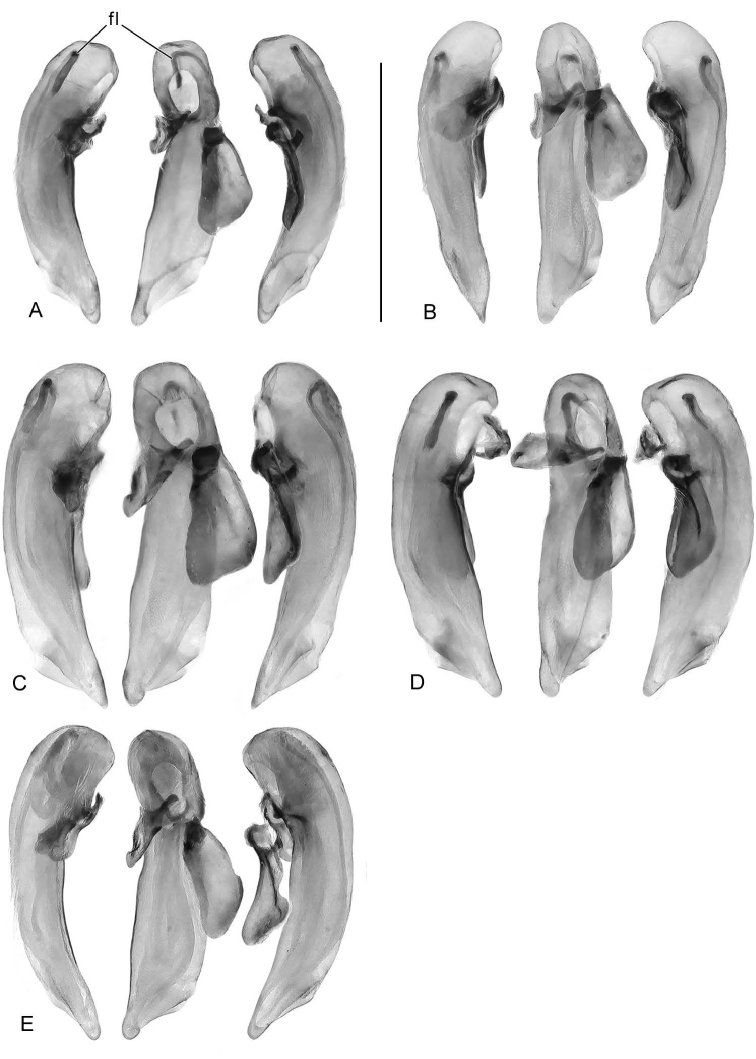
Digital Photo-illustration, male aedeagus in repose, dorsal, ventral, left lateral aspects: **A** *Hyboptera apollonia* Erwin, ADP100266 **B** *Hyboptera dilutior* Oberthür, ADP134143 **C** *Hyboptera lucida* Henry & Erwin, sp. n., ADP135785 **D** *Hyboptera tuberculata* (Dejean), ADP135797 **E** *Hyboptera verrucosa* (Reiche), ADP082523. Legend, fl, flagellum. Scale line = 0.25 mm.

(cf. Figs 1–10). Neck broad; eyes very large, hemispherical; frons markedly rugose, more or less anteriorly depressed. Prothorax wider than head; sides of pronotum broadly reflexed throughout, rounded or subangulate at mid-lateral setigerous pore. Elytron at basal third very slightly transversely depressed, surface uneven, markedly tuberculate overall. Flight wings of a dusky color. Basitarsus of mid and hind legs markedly elongate, coequal to length of tarsomeres 2-5 combined; males with adhesive setae on tarsomeres 1–3. Endophallus with flagellum; flagellum extruded at apex in many specimens.

#### Dispersal potential.

The wings are fully developed in adults of all known species, thus it is likely these beetles are moderate to strong flyers.

#### Geographic distribution.

A widespread southern Nearctic and Neotropical genus known from Texas, USA, south to southeastern Brazil, in the west to Bolivia, and east to French Guiana.

#### Ways of life.

Much is known about the species in this genus and that is reported here for the first time. Adults of various species are regularly collected in both the wet and dry seasons using insecticidal fogging techniques in many species of trees reaching the forest canopy in the Amazon Basin, thus they are certainly mainly arboreal. They are good flyers as evidenced by their capture in Malaise traps, C.D.C. mosquito traps, FITs, and at different types of light traps. At Barro Colorado Island in Panamá, one of us (TLE) collected adults from the forest floor amongst the large shed anther rings of trees of the species *Pseudobombax septenatum* (Jacq.) Dugand; these rings being a moisture source on the forest floor during the dry season (Erwin 1991, 2004). Warren E. Steiner collected several larvae and pupae along with emerging adults of *Hyboptera verrucosa* (Reiche) under bark of a living fence post in Panamá. These immature stages have yet to be described and illustrated (Erwin and Steiner, in prep).

#### Notes.

Five species have been previously described in this genus, along with three species in its adelphotaxon, *Hybopteroides* Erwin & Ball, 2012. Apparently, Lorenz (1998) was unaware of the Reichardt (1973) paper designating a type species (*H. angulicollis* Chaudoir) for the genus and unnecessarily designated *H. tuberculata* (Dejean), as the type species. The former stands.

#### Included species.

The species list below, as well as the arrangement of descriptions that follows, is ordered alphabetically within two species groups.

##### *angulicollis* species group

*Hyboptera angulicollis* Chaudoir, 1872:164; Brazil – Pará; Colombia; Ecuador; French Guiana; Perú; Suriname.

*Hyboptera biolat* Erwin & Henry, **sp. n.**; Perú.

*Hyboptera vestiverdis* Henry & Erwin, **sp. n.**; Ecuador; Perú.

*Hyboptera scheelea* Erwin & Henry, **sp. n.**; Perú.

*Hyboptera shasta* Erwin, **sp. n.**; Brazil – Amazonas.

*Hyboptera tepui* Erwin & Henry, **sp. n.**; Venezuela.

*Hyboptera tiputini* Erwin & Henry, **sp. n.**; Colombia; Ecuador; Perú.

*Hyboptera viridivittis* Chaudoir, 1872:164; Brazil – Minas Gerais, Rio de Janeiro, Santa Catarina.

##### *tuberculata* species group

*Hyboptera apollonia* Erwin, 2004: 33; Costa Rica; Panamá.

*Hyboptera auxilidora* Erwin, 2004: 35; Costa Rica; Honduras; México – VC; Panamá, USA – TX.

*Hyboptera dilutior* Oberthür, 1884: 52; Brazil – Amazonas, Pará, Rondônia; Ecuador; French Guiana; Perú; Venezuela.

*Hyboptera lucida* Henry & Erwin, **sp. n.**; Ecuador; French Guiana.

*Hyboptera tuberculata* (Dejean), 1825: 272; Bolivia; Brazil – Amazonas, Sergipe; Colombia; Ecuador; Guyana; French Guiana; Perú; Suriname.

*Hyboptera verrucosa* (Reiche), 1842: 311; Brazil – Amazonas; Colombia; Ecuador; French Guiana; Panamá; Perú; Suriname; Trinidad and Tobago; Venezuela.

### Accounts of taxa

#### Key to the species of *Hyboptera* Chaudoir, 1872

**Table:** 

1	Adults with only dark non-metallic markings on the pronotal disc	**2**
1’	Adults with patches of bright metallic green para-medially on pronotum	**7**
1’’	Adults without or with only slightly darker, non-metallic pronotal marking	**11**
2(1)	Pronotum with discal rugae etched horizontally and linear (Fig. 6A)	***H. apollonia* Erwin**
2’	Pronotum with discal rugae etched at an angle aimed medio-posteriorly, or somewhat chaotically without clear order	**3**
3(2’)	Elytra black with 4 small medio-apical tubercles pale (Fig. 7B)	***H. lucida* Henry & Erwin, sp. n.**
3’	Elytra mostly testaceous or pale brownish with darkly marked tubercles; small medio-apical tubercles pale or not	**4**
4(3’)	Elytron with sutural margin at apical sixth black markedly contrasting with testaceous background color (Fig. 7A)	***H. dilutior* Oberthür**
4	Elytron with sutural margin at apical sixth pale, not black, if brownish not contrasting with background color	**5**
5(4’)	Elytron just posterior to scutellum with only the suture pale in color otherwise markedly infuscate (Fig. 9A)	***H. tuberculata* (Dejean)**
5’	Elytron just posterior to scutellum with a V-shaped pale area encompassing the suture and the first interval	**6**
6(5’)	Elytron narrow, almost parallel sided. Distribution from Panamá south into the Amazon Basin and across to French Guiana and Trinidad & Tobago (Fig. 9B)	***H. verrucosa* (Reiche)**
6’	Elytron broad with markedly arcuate lateral margin. Distribution from Panamá north to southernmost Texas (Fig. 6B)	***H. auxilidora* Erwin**
7(1’)	Elytra entirely metallic bronzy-green	**8**
7’	Elytra dark matte black or dark and markedly shiny, some individuals with hint of metallic green spots near basal margin	**9**
8(7)	Venter with meso- and metathorax mostly infuscated (Fig. 1B)	***H. biolat* Erwin & Henry, sp. n.**
8’	Venter entirely rufotestaceous (Fig. 1A)	***H. angulicollis* Chaudoir**
9(7’)	Elytra dark matte black (Fig. 4B)	***H. viridivittis* Chaudoir**
9’	Elytra markedly shiny	**10**
10(9’)	Elytron violaceous, broad; meso- and metathorax pale not contrasting with pale abdomen (Fig. 4A)	***H. tiputini* Erwin & Henry, sp. n.**
10’	Elytron dark olivaceous, narrow; meso- and metathorax infuscated, markedly contrasting with pale abdomen (Fig. 2A)	***H. vestiverdis* Henry & Erwin, sp. n.**
11(1’’)	Elytron with lateral margin broadly testaceous from humerus to sutural apex; apical abdominal tergite testaceous with slight infuscation at extreme posterior-lateral corners (Fig. 3B)	***H. tepui* Erwin & Henry, sp. n.**
11’.	Elytron with lateral margin narrowly testaceous to latero-apical corner, not reaching sutural apex; apical abdominal tergite mostly infuscated with narrow median testaceous stripe	**12**
12(11’)	Elytra brilliant metallic green throughout, size larger (Fig. 3A)	***H. shasta* Erwin, sp. n.**
12’.	Elytra blackish-blue with metallic green highlights across the humeri and green points at some larger tubercles (Fig. 2B)	***H. scheelea* Erwin & Henry, sp. n.**

#### *angulicollis* species group

(recognized by Reichardt 1973)

The most distinctive attribute of species in this group is that the pronotum has marked discal rugae etched almost horizontally and linear, as opposed to angulate. Adults of all have metallic green coloration somewhere on the dorsal surface and the general adult size is small to medium for the genus. Male phallus apex elongate, somewhat acuminate.

##### Hyboptera angulicollis

Taxon classificationAnimaliaColeoptera Carabidae

Chaudoir, 1872

**Figure 11.**
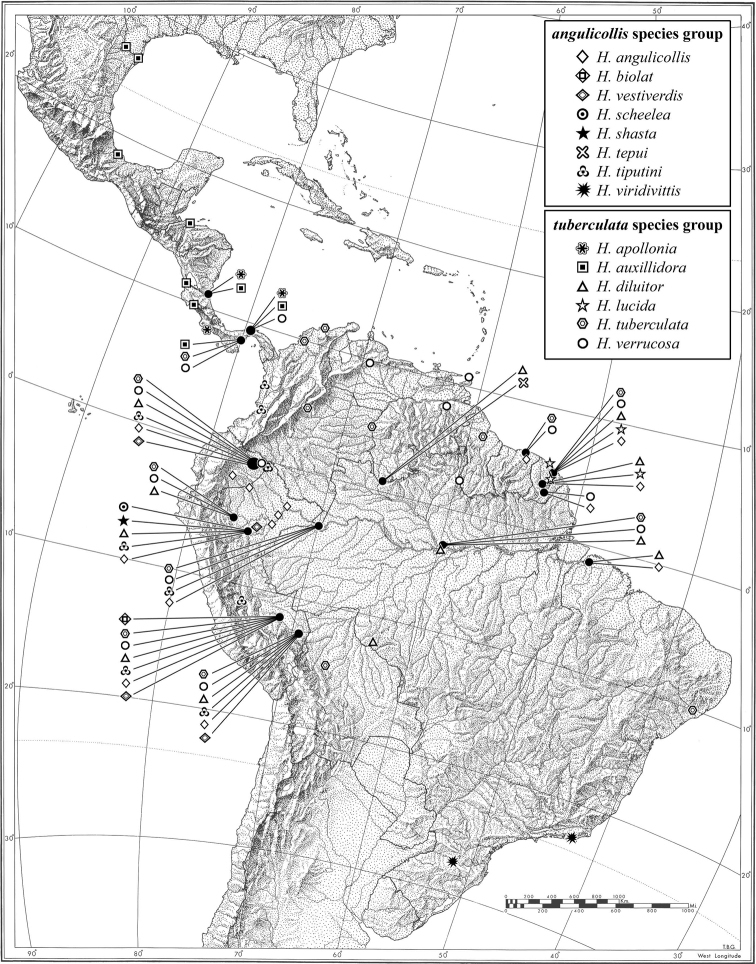
Distribution map for known localities of *Hyboptera* species.

Angled-neck humps-backed beetle Figs 1A , 5A , 11

Hyboptera angulicollis Chaudoir, 1872: 164.

###### Holotype.

Sex unknown. **Brazil**, Amazonas, Ega (Tefé), (HW Bates)(MNHP). Not seen by us; however, we have seen a “homotype” labelled by George E. Ball who studied the holotype in Paris.

###### Derivation of specific epithet.

The epithet, *angulicollis*, is a Latinized singular feminine adjective meaning “angled neck” referring to the angulate lateral sides of the pronotum.

###### Proposed english vernacular name.

Angled-neck humps-backed beetle.

###### Diagnosis.

With the attributes of the genus and *angulicollis* species group as described above and adults with patches of bright metallic green para-medially on pronotum, elytra entirely with metallic green reflection, and venter entirely rufotestaceous.

###### Description.

(Figs 1A, 5A). *Size*: See Appendix 1. Length (SBL) long for genus, ABL = 3.943–4.800 mm, SBL = 3.34–4.15 mm, TW = 1.92–2.53 mm.

*Color: See* diagnosis, above.

*Luster*: Metallic highlights, partially iridescent.

*Microsculpture*: Mostly isodiametric or slightly stretched, well-impressed, cells somewhat more stretched around elytral tubercles.

*Head*: Rugae moderately coarse, mostly transverse. Eye very large, sub-hemispheric, and evenly rounded anteriorly, subtly more prolonged posteriorly. Antenna short, barely reaching humerus. Labrum rectangulate, shallowly bilobed, anterior margin slightly emarginate. Neck finely transversely rugose.

*Prothorax*: Pronotum moderately broad, disc centrally planar with dense transverse rugae. Lateral margins broadly explanate and obtusely angled medially then subtly arcuate to broadly obtuse hind angle, base medially produced and rounded posteriorly.

*Pterothorax*: Normal for Agrina, fully winged. Elytron intervals 3 with 6, and 5 with 5 (4) discal unisetiferous tubercles, other intervals moderately convex, side margin moderately explanate laterally only at middle third. Elytron broad and moderately short, moderately narrower than the pronotum at the broadest part, apex truncate, slightly rounded with distal corner broadly and obtusely rounded, disc not significantly convex, basal third slightly depressed. All interneurs well-impressed.

*Legs*: Femur dorso-ventrally moderately depressed, tibia coequal in length, more depressed; tarsus less than half the length of the tibia, fourth tarsomere markedly bilobed and with tarsal pad of setae.

*Abdomen*: Sparsely setiferous; normal ambulatory setae on sterna 3–5; female with two pairs of ambulatory setae on sternum 6, medial pair of setae less than the length of lateral pair; males with only the lateral pair of longer setae on sternum 6.

*Male genitalia*: Phallus (Fig. 5A) with ostium of 1/5 its length, catopic, apex long, narrowly rounded; endophallus with flagellum (not obvious in illustration), flagellum not barbed. Parameres asymmetric, right very small, left larger.

*Female genitalia*: Not investigated, likely similar to that of *H. lucida* (Fig. 8).

*Variation*: In the large series of specimens of this species available to us for this study from many localities, we noted that the pronotal explanate margins vary considerably in width, the more narrow they are the more prominent the midpoint angle, the broader the explanation the more obtuse the midpoint angle.

###### Dispersal potential.

These beetles are macropterous and capable of flight. They are moderately swift and agile runners. Specimens have been acquired using most modern collecting methods, including insecticidal foggings, UV light sheets and traps, Malaise traps, SLAM Malaise traps, and flight intercept traps (FITs).

###### Way of life.

Adults are common in the lowlands (8 to 700 m a.s.l.) and appear to be generalists in a variety of rainforest biotopes including terra firme, várzea, igapó, second growth, subtropical moist and white sand forests. In these forests, they are commonly found in big trees with vines and epiphytes, in suspended dry leaves, under lianas close to the trunk, in dry *Scheelea* sp. and *Astrocaryum chambira* Burret palm fronds, in very large suspended dry *Cecropia* leaves, and in dry bamboo leaves of the genera *Guadua* [*G. weberbaueri* Pilg.] and *Elytrostachys* [sp.] Individuals can be found in all months of the year, in both the rainy and dry seasons. Member of this species have been recorded from the canopy of the following tree species using insecticidal fogging techniques: *Allophyllus* sp.; Pourouma bicolor ssp. bicolor cf., *Pourouma cecropiifolia* aff., Pourouma mollis ssp. triloba cf.; *Virola obovata*, *Virola decorticans* cf.; Brosimum utile ssp. ovatifolium cf., *Aspidosperma darienense* cf.; *Crepidospermum rhoifolium*; *Castilla ulei* cf.; *Pseudolmedia laevis*, *Pseudolmedia macrophylla* cf.; *Hymenaea oblongifolia*; *Matisia malacocalyx* cf.; *Oenocarpus bataua*; *Siparuna decipiens*; Trattinnickia rhoifolia var. lancifolia cf.; *Cecropia herthae*, *Cecropia ficifolia*; *Pentagonia spathicalyx* cf.; Pouteria reticulata ssp. reticulata cf., Pouteria cuspidata ssp. robusta cf., *Pouteria baehniana* cf., *Pouteria rostrata* aff. añangu; *Gustavia longifolia*; *Leonia glycicarpa*; *Protium sagotianum* cf., *Protium nodulosum* cf.; *Sloanea cordia*; *Iriartea deltoidea*; *Browneopsis ucayalina*; *Eschweilera coriacea* cf., *Eschweilera juruensis* cf.; *Batocarpus orinocensis* cf.; *Pachira aquatica* cf.; *Sarcaulus brasiliensis* aff. burnt; *Blakea* sp. 1; *Parkia multijuga* cf.; *Swartzia león*; *Rhodostemonodaphne kunthiana* cf.; *Nectandra* sp. 1, *Nectandra crassiloba* cf.; *Inga olivaceae*, *Inga capitata*, *Inga bourgonii* cf., *Inga cuadra*; *Rinorea viridifolia*; *Talisia* bitter; *Coussapoa orthoneura* cf., *Coussapoa herthae*; *Aspidosperma darienense* cf.; *Vismia* weedy; *Astrocaryum chambira*; *Tabebuia moby*; *Wittmackanthus stanleyanus* cf.; *Alchornea triplinervia* cf.; *Maquira calophylla*; *Trichilia elsae* cf., *Trichilia solitudinis*; *Trichilia solitudinis*; *Simira cordifolia*/*rubescens* cf.; *Catatola costaricana* aff.; *Brownea grandiceps* cf.; *Pausandra trianae*; *Micropholis venulosa* cf.; *Sagotia racemosa*; *Diospyros sericea*; *Guarea silvatica*; Sorocea pubivena ssp. oligotricha cf.; *Grias neuberthii*; *Simaba guianensis* cf.; *Tapura peruviana* cf.; *Neea* dive-tuberculate; *Semaphyllanthe megistocaula* cf., *Semaphyllanthe garcinia macrophylla* cf.; *Guatteria* sp. 3, sect.; *Meiocarpus*, long petiole.

**Other specimens examined. Brazil**, Pará, Belém, Mocambo, 1.555°S, 48.429°W, 25m, 10 January 1978 (NMNH: ADP007291, female), 20 May 1978 (NMNH: ADP007290, female), 23 June 1978 (NMNH: ADP007289, female). **Colombia**, Amazonas, PNN Amacayacu, February 1989 (M Kelsey)(IAvH: IAvH-E-2434, ADP145214, IAvH-E-2435, ADP145200, males), IAvH-E-2436, ADP145206, IAvH-E-2437, ADP145202, IAvH-E-2438, ADP145204, IAvH-E-73774, ADP145210, females), 100m, July 1988 (M Kelsey)(IAvH: IAvH-E-40649, ADP145215, female), 100m, 19 January 1998 (A Alvarado)(IAvH: IAvH-E-2439, ADP145217, female), 150-200m, 31 August 1997 (D Campos)(IAvH: IAvH-E-73773, ADP145149, male), 1 September 1997 (D Campos, F Fernandez)(IAvH: IAvH-E-73773, ADP145147, male, IAvH-E-2442, ADP145191, female), Boca Caño Matamata, 3.3839°S, 70.0999°W, 150m, 30 August 1997 (Brown, G Kung)(IAvH: IAvH-E-2441, ADP145209m, female), Caño Matamata, 3°23'S, 070°06'W, 150m, 11-17 December 2000 (A Parente)(IAvH: IAvH-E-3534, ADP145195, male), San Martin, 3.776°S, 070.301°W, 80m, 22-30 April 2000 (B Amado)(IAvH: IAvH-E-73757, ADP145189, female), 2-7 July 2000 (B Amado)(IAvH: IAvH-E-73763, ADP145211, female), 13-21 December 2000 (B Amado)(IAvH: IAvH-E-3533, ADP145197, female), Caquetá, Florencia, Santa Rosita, 01°20'N, 076°6'W, 600m, 7-22 July 2000 (E González)(IAvH: IAvH-E-2284, ADP145160, IAvH-E-40757, ADP145162, males), 22 July - 4 August 2000 (F )(IAvH: IAvH-E-73770, ADP145164, female), PNN Chiribiquete, Puerto Abeja, 00°4'N, 072°26'W, 310m, 29 October - 12 November 2000, M Ospina, E González)(IAvH: IAvH-E-2699, ADP145167, IAvH-E-73765, ADP145156, IAvH-E-73766, ADP145158, females), PNN Chiribiquete, Puerto Solano, 00°12'N, 072°25'W, 300m, 19-22 February 2001 (M Ospina, E González)(IAvH: IAvH-E-73771, ADP145198, male), 26 February - 3 March 2001 (M Ospina, E González)(IAvH: IAvH-E-73772, ADP145192, female), 12-19 November 2000 (J Forero)(IAvH: IAvH-E-73767, ADP145172, female), PNN Chiribiquete, Rio Cuñare, 00°30'N, 072°37'W, 300m, 1-4 November 2000 (E González, M Ospina)(IAvH: IAvH-E-73760, ADP145144, female), 1-5 November 2000 (E González, M Ospina)(IAvH: IAvH-E-73768, ADP145150, male), Meta, 8 km SW San Jaun de Arama, 4.2242°S, 69.9449°W, 70m, 27 July 2015 (Jäch)(NMNH, ADP140534, female), Nariño, Territoio Kofan, Orito, 0.499°N, 77.216°W, 700m, 28 September 1998 (E González)(IAvH: IAvH-E-2440, ADP145146, male). **Ecuador**, Napo, Tena, 0.989°S, 77.812°W, 500m, 22 -27 May 1987 (BV Brown)(LACM: ADP006159, male), Orellana, Reserva Ethnica Huaorani, 39 km S Pompeya, Estación Científica Yasuní - Onkone Gare Camp, Erwin Piraña Plot: transect 6, station 2, 0.6561°S, 76.4483°W, 220-250m, 15 January 1994 (TL Erwin, et al.)(NMNH: ADP138234, male), Erwin Piraña Plot: transect 4, station 8, 0.6570°S, 76.4498°W, 220-250m, 16 January 1994 (TL Erwin, et al.)(NMNH: ADP135890, female), Erwin Piraña Plot: transect 7, station 9, 0.6556°S, 76.4475°W, 220-250m, 20 January 1994 (TL Erwin, et al.)(NMNH: ADP135903, male), Erwin Piraña Plot: transect 7, station 6, 0.6556°S, 76.4475°W, 220-250m, 20 January 1994 (TL Erwin, et al.)(NMNH: ADP135905, males), Erwin Piraña Plot: transect 8, station 1, 0.6551°S, 76.4403°W, 220-250m, 24 January 1994 (TL Erwin, et al.)(NMNH: ADP138676, male), Erwin Piraña Plot: transect 8, station 6, 0.6551°S, 76.4403°W, 220-250m, 24 January 1994 (TL Erwin, et al.)(NMNH: ADP135960, male), Erwin Piraña Plot: transect 4, station 9, 0.6570°S, 76.4498°W, 220-250m, 25 June 1994 (TL Erwin, et al.)(NMNH: ADP135968, female), Erwin Piraña Plot: transect 5, station 10, 0.6566°S, 76.4490°W, 220-250m, 25 June 1994 (TL Erwin, et al.)(NMNH: ADP135978, male), Erwin Piraña Plot: transect 2, station 2, 0.6556°S, 76.4475°W, 220-250m, 20 June 1994 (TL Erwin, et al.)(NMNH: ADP135899, ADP135984, males), Erwin Piraña Plot: transect 10, station 6, 0.6540°S, 76.4453°W, 220-250m, 29 June 1994 (TL Erwin, et al.)(NMNH: ADP135958, male), Erwin Piraña Plot: transect 8, station 5, 0.6551°S, 76.4403°W, 220-250m, 21 June 1994 (TL Erwin, et al.)(NMNH: ADP135956, male), Erwin Piraña Plot: transect 9, station 9, 0.6545°S, 76.4460°W, 220-250m, 21 June 1994 (TL Erwin, et al.)(NMNH: ADP135909, female), Erwin Piraña Plot: transect 10, station 10, 0.6540°S, 76.4453°W, 220-250m, 29 June 1994 (TL Erwin, et al.)(NMNH: ADP135904, female), Erwin Piraña Plot: transect 1, station 10, 0.6586°S, 76.4521°W, 220-250m, 3 July 1994 (TL Erwin, et al.)(NMNH: ADP135791, male, ADP135856, female), Erwin Piraña Plot: transect 1, station 8, 0.6586°S, 76.4521°W, 220-250m, 4 October 1994 (TL Erwin, et al.)(NMNH: ADP135895, female), Erwin Piraña Plot: transect 2 station 7, 0.6581°S, 76.4513°W, 220-250m, 4 October 1994 (TL Erwin, et al.)(NMNH: ADP135893, male), Erwin Piraña Plot: transect 10, station 10, 0.6540°S, 76.4433°W, 220-250m, 6 October 1994 (TL Erwin, et al.)(NMNH: ADP137733, female), Erwin Piraña Plot: transect 8, station 10, 0.6551°S, 76.4403°W, 220-250m, 7 October 1994 (TL Erwin, et al.)(NMNH: ADP006160, females), Erwin Piraña Plot: transect 5, station 7, 0.6566°S, 76.4490°W, 220-250m, 9 October 1994 (TL Erwin, et al.)(NMNH: ADP135907, female), Erwin Piraña Plot: transect 4, station 10, 0.6570°S, 76.4498°W, 220-250m, 10 October 1994 (TL Erwin, et al.)(NMNH: ADP137736, female), Erwin Piraña Plot: transect 9, station 4, 0.6545°S, 76.4460°W, 220-250m, 8 February 1995 (TL Erwin, et al.)(NMNH: ADP135972, male), Erwin Piraña Plot: transect 9, station 10, 0.6545°S, 76.4460°W, 220-250m, 8 February 1995 (TL Erwin, et al.)(NMNH: ADP135944, male, ADP135936, female), Erwin Piraña Plot: transect 10, station 3, 0.6540°S, 76.4473°W, 220-250m, 8 February 1995 (TL Erwin, et al.)(NMNH: ADP135942, male), Erwin Piraña Plot: transect 2, station 8, 0.6581°S, 76.4513°W, 220-250m, 9 February 1995 (TL Erwin, et al.)(NMNH: ADP137846, female), Erwin Piraña Plot: transect 6, station 10, 0.6561°S, 76.4483°W, 220-250m, 9 February 1995 (TL Erwin, et al.)(NMNH: ADP137357, female), Erwin Piraña Plot: transect 7, station 8, 0.6556°S, 76.4475°W, 220-250m, 10 February 1995 (TL Erwin, et al.)(NMNH: ADP137740, male), Erwin Piraña Plot: transect 4, station 2, 0.6566°S, 76.4490°W, 220-250m, 11 February 1995 (TL Erwin, et al.)(NMNH: ADP137379, male, ADP137339, female), Erwin Piraña Plot: transect 4, station 7, 0.6570°S, 76.4498°W, 220-250m, 3 July 1995 (TL Erwin, et al. (NMNH: ADP006172, female), Erwin Piraña Plot: transect 3, station 9, 0.6566°S, 76.4490°W, 220-250m, 6 October 1995 (TL Erwin, et al.)(NMNH: ADP135952, male), Erwin Piraña Plot: transect 5, station 7, 0.6566°S, 76.4490°W, 220-250m, 6 October 1995 (TL Erwin, et al.)(NMNH: ADP137325, ADP137373, males, ADP137365, female), Erwin Piraña Plot: transect 6, station 1, 0.6561°S, 76.4483°W, 220-250m, 6 October 1995 (TL Erwin, et al.)(NMNH: ADP137327, female), Erwin Piraña Plot: transect 6, station 6, 0.6561°S, 76.4483°W, 220-250m, 6 October 1995 (TL Erwin, et al.)(NMNH: ADP137391, female), Erwin Piraña Plot: transect 6, station 10, 0.6561°S, 76.44838°W, 220-250m, 6 October 1995 (TL Erwin, et al.)(NMNH: ADP137375, female), Erwin Piraña Plot: transect 8, station 7, 0.6551°S, 76.4403°W, 220-250m, 7 October 1995 (TL Erwin, et al.)(NMNH: ADP135988, female), Erwin Piraña Plot: transect 9, station 8, 0.6545°S, 76.4460°W, 220-250m, 8 October 1995 (TL Erwin, et al.)(NMNH: ADP135982, male), Erwin Piraña Plot: transect 9, station 9, 0.6545°S, 76.4460°W, 220-250m, 8 October 1995 (TL Erwin, et al.)(NMNH: ADP135946, male), Erwin Piraña Plot: transect 10, station 2, 0.6540°S, 76.4453°W, 220-250m, 8 October 1995 (TL Erwin, et al.)(NMNH: ADP135938, male, ADP135948, female), Erwin Piraña Plot: transect 10, station 4, 0.6540°S, 76.4453°W, 220-250m, 8 October 1995 (TL Erwin, et al.)(NMNH: ADP135966, male), Erwin Piraña Plot: transect 10, station 5, 0.6540°S, 76.4453°W, 220-250m, 8 October 1995 (TL Erwin, et al.)(NMNH: ADP135964, female), Erwin Piraña Plot: transect 2, station 3, 0.6581°S, 76.4513°W, 220-250m, 9 October 1995 (TL Erwin, et al.)(NMNH: ADP135915, male), Erwin Piraña Plot: transect 2, station 7, 0.6581°S, 76.4513°W, 220-250m, 4 February 1996 (TL Erwin, et al.)(NMNH: ADP137696, male), Erwin Piraña Plot: transect 2, station 8, 0.6581°S, 76.4513°W, 220-250m, 4 February 1996 (TL Erwin, et al.)(NMNH: ADP135980, female), Erwin Piraña Plot: transect 4, station 6, 0.6576°S, 76.4498°W, 220-250m, 5 February 1996 (TL Erwin, et al.)(NMNH: ADP136473, male), Erwin Piraña Plot: transect 6, station 8, 0.6561°S, 76.4483°W, 220-250m, 7 February 1996 (TL Erwin, et al.)(NMNH: ADP137329, female), Erwin Piraña Plot: transect 10, 0.6586°S, 76.4521°W, 220-250m, 13 February 1996 (TL Erwin, et al.)(NMNH: ADP147731, male), Erwin Piraña Plot: transect 4, station 7, 0.6570°S, 76.4498°W, 220-250m, 21 June 1996 (TL Erwin, et al.)(NMNH: ADP137363, male), Erwin Piraña Plot: transect 9, station 4, 0.6570°S, 76.4498°W, 220-250m, 21 June 1996 (TL Erwin, et al.)(NMNH: ADP137343, male), Erwin Piraña Plot: transect 5, station 3, 0.6566°S, 76.4490°W, 220-250m, 22 June 1996 (TL Erwin, et al.)(NMNH: ADP137345, male), Erwin Piraña Plot: transect 5, station 9, 0.6566°S, 76.4490°W, 220-250m, 22 June 1996 (TL Erwin, et al.)(NMNH: ADP137347, male), Erwin Piraña Plot: transect 6, station 9, 0.6561°S, 76.4483°W, 220-250m, 22 June 1996 (TL Erwin, et al.)(NMNH: ADP137688, female), Erwin Piraña Plot: transect 9, station 7, 0.6545°S, 76.4460°W, 220-250m, 23 June 1996 (TL Erwin, et al.)(NMNH: ADP137744, male, ADP137742, female), Erwin Piraña Plot: transect 9, station 9, 0.6595°S, 76.4460°W, 220-250m, 23 June 1996 (TL Erwin, et al.)(NMNH: ADP137734, male), Erwin Piraña Plot: transect 1, station 3, 0.6586°S, 76.4521°W, 220-250m, 25 June 1996 (TL Erwin, et al.)(NMNH: ADP137748, male), Erwin Piraña Plot: transect 1, station 10, 0.6586°S, 76.4521°W, 220-250m, 30 September 1996 (TL Erwin, et al.)(NMNH: ADP137331, ADP137359, males), Erwin Piraña Plot: transect 2, station 7, 0.6581°S, 76.4513°W, 220-250m, 30 September 1996 (TL Erwin, et al.)(NMNH: ADP137361, female), Erwin Piraña Plot: transect 8, station 7, 0.6561°S, 76.4483°W, 220-250m, 2 October 1996 (TL Erwin, et al.)(NMNH: ADP135928, male), Erwin Piraña Plot: transect 7, station 3, 0.6566°S, 76.4490°W, 220-250m, 3 October 1996 (TL Erwin, et al.)(NMNH: ADP135913, male), Erwin Piraña Plot: transect 8, station 5, 0.6551°S, 76.4403°W, 220-250m, 3 October 1996 (TL Erwin, et al.)(NMNH: ADP135920, male), Erwin Piraña Plot: transect 8, station 10, 0.6551°S, 76.4403°W, 220-250m, 3 October 1996 (TL Erwin, et al.)(NMNH: ADP137385, male, ADP137367, female), Erwin Piraña Plot: transect 9, station 5, 0.6545°S, 76.4460°W, 220-250m, 4 October 1996 (TL Erwin, et al.)(NMNH: ADP137351, female), Erwin Piraña Plot: transect 5, station 2, 0.6566°S, 76.4490°W, 220-250m, 6 October 1996 (TL Erwin, et al.)(NMNH: ADP135862, female), Erwin Piraña Plot: transect 4, station 7, 0.6570°S, 76.4498°W, 220-250m, 20 January 2006 (TL Erwin, et al.)(NMNH: ADP133848, female), Erwin Piraña Plot: transect 5, station 1, 0.6566°S, 76.4490°W, 220-250m, 20 January 2006 (TL Erwin, et al.)(NMNH: ADP133854, female), Erwin Piraña Plot: transect 8, station 4, 0.6556°S, 76.4475°W, 220-250m, 28 January 2006 (TL Erwin, et al.)(NMNH: ADP133866, male), Erwin Piraña Plot: transect 1, station 1, 0.6586°S, 76.4421°W, 220-250m, 7 July 2006 (TL Erwin, et al.)(NMNH: ADP133856, male), Erwin Piraña Plot: transect 1, station 2, 0.6586°S, 76.4421°W, 220-250m, 7 July 2006 (TL Erwin, et al.)(NMNH: ADP133858, male), Erwin Piraña Plot: transect 6, station 7, 0.6561°S, 76.4483°W, 220-250m, 9 July 2006 (TL Erwin, et al.)(NMNH: ADP133850, male), Erwin Piraña Plot: transect 9, station 5, 0.6545°S, 76.4460°W, 220-250m, 12 July 2006 (TL Erwin, et al.)(NMNH: ADP133862, male), Yasuni National Park (edge), 95.43 km E (heading 101.46°) Coca, Tiputini Biodiversity Station, Erwin Harpia Plot: transect 3, station 9, 0.63327°S, 76.1443°W, 207m, 20 June 1998 (TL Erwin, et al.)(NMNH: ADP135860, ADP135906, females), Erwin Harpia Plot: transect 4, station 8, 0.63167°S, 76.1443°W, 208m, 30 June 1998 (TL Erwin, et al.)(NMNH: ADP135795, male), Erwin Harpia Plot: transect 6, station 10, 0.6295°S, 76.1443°W, 199m, 1 July 1998 (TL Erwin, et al.)(NMNH: ADP135834, male), Erwin Harpia Plot: transect 7, station 10, 0.6287°S, 76.1443°W, 203m, 4 July 1998 (TL Erwin, et al.)(NMNH: ADP135868, male), Erwin Harpia Plot: transect 8, station 5, 0.6278°S, 76.1443°W, 203m, 4 July 1998 (TL Erwin, et al.)(NMNH: ADP135910, male), Erwin Harpia Plot: transect 9, station 7, 0.6269°S, 76.1443°W, 203m, 5 July 1998 (TL Erwin, et al.)(NMNH: ADP135841, male), Erwin Harpia Plot: transect 10, station 6, 0.6262°S, 76.1443°W, 214m, 5 July 1998 (TL Erwin, et al.)(NMNH: ADP135854, male), Erwin Harpia Plot: transect 10, station 8, 0.6287°S, 76.1443°W, 214m, 5 July 1998 (TL Erwin, et al.)(NMNH: ADP135838, male), Erwin Harpia Plot: transect 9, station 6, 0.6269°S, 76.1443°W, 203m, 21 October 1998 (TL Erwin, et al.)(NMNH: ADP136756, female), Erwin Harpia Plot: transect 9, station 9, 0.6269°S, 76.1443°W, 203m, 21 October 1998 (TL Erwin, et al.)(NMNH: ADP135866, male), Erwin Harpia Plot: transect 10, station 3, 0.6262°S, 76.1443°W, 214m, 21 October 1998 (TL Erwin, et al.)(NMNH: ADP135882, female), Erwin Harpia Plot: transect 10, station 6, 0.6262°S, 76.1443°W, 215m, 21 October 1998 (TL Erwin, et al.)(NMNH: ADP135846, male), Erwin Harpia Plot: transect 7, station 1, 0.6287°S, 76.1443°W, 203m, 22 October 1998 (TL Erwin, et al.)(NMNH: ADP135884, male, ADP135888, female), Erwin Harpia Plot: transect 8, station 8, 0.6273°S, 76.1443°W, 203m, 22 October 1998 (TL Erwin, et al.)(NMNH: ADP136798, male), Erwin Harpia Plot: transect 8, station 10, 0.6287°S, 76.1443°W, 203m, 22 October 1998 (TL Erwin, et al.)(NMNH: ADP135864, female), Erwin Harpia Plot: transect 2, station 4, 0.6332°S, 76.1443°W, 197m, 23 October 1998 (TL Erwin, et al.)(NMNH: ADP135839, male), Erwin Harpia Plot: transect 2, station 8, 0.6332°S, 76.1443°W, 197m, 23 October 1998 (TL Erwin, et al.)(NMNH: ADP135880, female), Erwin Harpia Plot: transect 3, station 2, 0.6332°S, 76.1443°W, 207m, 24 October 1998 (TL Erwin, et al.)(NMNH: ADP135878, male), Erwin Harpia Plot: transect 3, station 3, 0.6332°S, 76.1443°W, 207m, 24 October 1998 (TL Erwin, et al.)(NMNH: ADP135769, male), Erwin Harpia Plot: transect 3, station 4, 0.6332°S, 76.1443°W, 207m, 24 October 1998 (TL Erwin, et al.)(NMNH: ADP135892, male), Erwin Harpia Plot: transect 3, station 9, 0.6332°S, 76.1443°W, 207m, 24 October 1998 (TL Erwin, et al.)(NMNH: ADP135912, female), Erwin Harpia Plot: transect 4, station 5, 0.6316°S, 76.1443°W, 208m, 24 October 1998 (TL Erwin, et al.)(NMNH: ADP137335, male, ADP135872, female), Erwin Harpia Plot: transect 6, station 5, 0.6295°S, 76.1443°W, 199m, 26 October 1998 (TL Erwin, et al.)(NMNH: ADP135837, male), Erwin Harpia Plot: transect 5, station 2, 0.6304°S, 76.1443°W, 209m, 2 February 1999 (TL Erwin, et al.)(NMNH: ADP139104, female), Erwin Harpia Plot: transect 10, station 6, 0.6262°S, 76.1443°W, 214m, 5 February 1999 (TL Erwin, et al.)(NMNH: ADP135886, male), Erwin Harpia Plot: transect 10, station 7, 0.6262°S, 76.1443°W, 214m, 5 February 1999 (TL Erwin, et al.)(NMNH: ADP135815, male), Erwin Harpia Plot: transect 7, station 3, 0.6287°S, 76.1443°W, 203m, 6 February 1999 (TL Erwin, et al.)(NMNH: ADP135876, female), Erwin Harpia Plot: transect 8, station 4, 0.6278°S, 76.1443°W, 203m, 6 February 1999 (TL Erwin, et al.)(NMNH: ADP135842, female), Erwin Harpia Plot: transect 5, station 7, 0.6304°S, 76.1443°W, 209m, 7 February 1999 (TL Erwin, et al.)(NMNH: ADP135817, male), Erwin Harpia Plot: transect 6, station 2, 0.6295°S, 76.1443°W, 199m, 7 February 1999 (TL Erwin, et al.)(NMNH: ADP135914, female), Erwin Harpia Plot: transect 6, station 6, 0.6295°S, 76.1443°W, 199m, 7 February 1999 (TL Erwin, et al.)(NMNH: ADP135894, female), Erwin Harpia Plot: transect 4, station 6, 0.6316°S, 76.1443°W, 208m, 8 February 1999 (TL Erwin, et al.)(NMNH: ADP135852, female), Erwin Harpia Plot: transect 1, station 2, 0.6342°S, 76.1443°W, 218m, 9 February 1999 (TL Erwin, et al.)(NMNH: ADP135858, male), Erwin Harpia Plot: transect 1, station 3, 0.6332°S, 76.1443°W, 207m, 21 October 1999 (TL Erwin, et al.)(NMNH: ADP135918, female), Erwin Harpia Plot: transect 10, station 5, 0.6262°S, 76.1443°W, 214m, 22 October 1999 (TL Erwin, et al.)(NMNH: ADP135901, male), Erwin Harpia Plot: transect 10, station 6, 0.6262°S, 76.1443°W, 214m, 22 October 1999 (TL Erwin, et al.)(NMNH: ADP137694, male), Erwin Harpia Plot: transect 3, station 8, 0.6232°S, 76.1443°W, 207m, 23 October 1999 (TL Erwin, et al.)(NMNH: ADP139521, male), Erwin Harpia Plot: transect 6, station 9, 0.6287°S, 76.1443°W, 199m, 24 October 1999 (TL Erwin, et al.)(NMNH: ADP137754, male), Erwin Harpia Plot: transect 6, station 3, 0.6295°S, 76.1443°W, 199m, 25 October 1999 (TL Erwin, et al.)(NMNH: ADP137758, female), Erwin Harpia Plot: transect 9, station 8, 0.6269°S, 76.1443°W, 203m, 28 September 2000 (TL Erwin, et al.)(NMNH: ADP139152, female), Erwin Harpia Plot: transect 9, station 9, 0.6269°S, 76.1443°W, 203m, 28 September 2000 (TL Erwin, et al.)(NMNH: ADP139160, male), Erwin Harpia Plot: transect 10, station 4, 0.6262°S, 76.1443°W, 214m, 28 September 2000 (TL Erwin, et al.)(NMNH: ADP139154, ADP139142, ADP139144, males), Erwin Harpia Plot: transect 4, station 5, 0.6316°S, 76.1443°W, 208m, 1 October 2000 (TL Erwin, et al.)(NMNH: ADP139162, female), Erwin Harpia Plot: transect 4, station 6, 0.6316°S, 76.1443°W, 208m, 1 October 2000 (TL Erwin, et al.)(NMNH: ADP139158, male), Erwin Harpia Plot: transect 5, station 3, 0.6316°S, 76.1443°W, 214m, 1 October 2000 (TL Erwin, et al.)(NMNH: ADP139136, female), Erwin Harpia Plot: transect 6, station 1, 0.6304°S, 76.1443°W, 199m, 1 October 2000 (TL Erwin, et al.)(NMNH: ADP139126, female), Erwin Harpia Plot: transect 6, station 3, 0.6304°S, 76.1443°W, 199m, 1 October 2000 (TL Erwin, et al.)(NMNH: ADP139092, ADP139164, females), Erwin Harpia Plot: transect 6, station 5, 0.6295°S, 76.1443°W, 199m, 1 October 2000 (TL Erwin, et al.)(NMNH: ADP139130, male), Erwin Harpia Plot: transect 1, station 3, 0.6316°S, 76.1443°W, 214m, 2 October 2000 (TL Erwin, et al.)(NMNH: ADP139122, male), Erwin Harpia Plot: transect 1, station 5, 0.6342°S, 76.1443°W, 214m, 2 October 2000 (TL Erwin, et al.)(NMNH: ADP139146, female), Erwin Harpia Plot: transect 1, station 6, 0.6342°S, 76.1443°W, 214m, 2 October 2000 (TL Erwin, et al.)(NMNH: ADP139094, male), Erwin Harpia Plot: transect 6, station 1, 0.6342°S, 76.1443°W, 214m, 2 October 2000 (TL Erwin, et al.)(NMNH: ADP139096, female), Erwin Harpia Plot: transect 3, station 10, 0.6330°S, 76.1443°W, 207m, 4 October 2000 (TL Erwin, et al.)(NMNH: ADP139148, female), Erwin Harpia Plot: transect 10, station 4, 0.6332°S, 76.1443°W, 207m, 4 October 2000 (TL Erwin, et al.)(NMNH: ADP139156, male), Erwin Harpia Plot: transect 1, station 4, 0.6342°S, 76.1443°W, 214m, 16 February 2001 (TL Erwin, et al.)(NMNH: ADP139114, female), Erwin Harpia Plot: transect 2, station 2, 0.6332°S, 76.1443°W, 197m, 16 February 2001 (TL Erwin, et al.)(NMNH: ADP139110, male), Erwin Harpia Plot: transect 9, station 7, 0.6269°S, 76.1443°W, 203m, 17 February 2001 (TL Erwin, et al.)(NMNH: ADP139116, female), Erwin Harpia Plot: transect 9, station 8, 0.6269°S, 76.1443°W, 203m, 17 February 2001 (TL Erwin, et al.)(NMNH: ADP139102, female), Erwin Harpia Plot: transect 9, station 9, 0.6268°S, 76.1443°W, 203m, 17 February 2001 (TL Erwin, et al.)(NMNH: ADP139120, male, ADP139118, female), Erwin Harpia Plot: transect 1, station 2, 0.6342°S, 76.1443°W, 214m, 19 July 2001 (TL Erwin, et al.)(NMNH: ADP139463, female), Erwin Harpia Plot: transect 1, station 3, 0.6342°S, 76.1443°W, 214m, 19 July 2001 (TL Erwin, et al.)(NMNH: ADP139469, male), Erwin Harpia Plot: transect 1, station 8, 0.6342°S, 76.1443°W, 214m, 19 July 2001 (TL Erwin, et al.)(NMNH: ADP139106, male), Erwin Harpia Plot: transect 10, station 1, 0.6262°S, 76.1443°W, 214m, 20 July 2001 (TL Erwin, et al.)(NMNH: ADP139523, male), Erwin Harpia Plot: transect 10, station 3, 0.6269°S, 76.1443°W, 214m, 20 July 2001 (TL Erwin, et al.)(NMNH: ADP139465, male), Erwin Harpia Plot: transect 9, station 7, 0.6269°S, 76.1443°W, 203m, 20 July 2001 (TL Erwin, et al.)(NMNH: ADP139467, male), Erwin Harpia Plot: transect 10, station 4, 0.6262°S, 76.1443°W, 214m, 20 July 2001 (TL Erwin, et al.)(NMNH: ADP139525, male), Erwin Harpia Plot: transect 3, station 1, 0.6332°S, 76.1443°W, 207m, 24 July 2001 (TL Erwin, et al.)(NMNH: ADP139461, male); Sucumbíos, Río Napo, Sacha Lodge, Pilchicocha, 0.472°S, 76.459°W, 228m, 12-22 February 1994 (P Hibbs)(SEMC: ADP006158, female) Río Napo, Sacha Lodge, Pilchicocha, 0.472°S, 76.459°W, 228m, 22 February - 4 March 1994 (P Hibbs)(SEMC: ADP006156) Río Napo, Sacha Lodge, Pilchicocha, 0.472°S, 76.459°W, 228m, 4-14 March 1994 (P Hibbs)(SEMC: ADP006112, ADP006131, ADP006155, females) Río Napo, Sacha Lodge, Pilchicocha, 0.472°S, 76.459°W, 228m, 14-24 March 1994 (P Hibbs)(SEMC: ADP006114, ADP006154, females) Río Napo, Sacha Lodge, Pilchicocha, 0.472°S, 76.459°W, 228m, 3-13 April 1994 (P Hibbs)(SEMC: ADP006137, male, ADP006128, ADP006136, females) Río Napo, Sacha Lodge, Pilchicocha, 0.472°S, 76.459°W, 228m, 13-23 April 1994 (P Hibbs)(SEMC: ADP006151, male, ADP006152, female) Río Napo, Sacha Lodge, Pilchicocha, 0.472°S, 76.459°W, 228m, 23 April - 4 May 1994 (P Hibbs)(SEMC: ADP006129, ADP006134, males, ADP006113, ADP006135, female), 13-25 July 1994 (P Hibbs)(SEMC: ADP006115, male, ADP006153, female), 3-16 August 1994 (P Hibbs)(SEMC: ADP006157, male), 20-30 September 1994 (P Hibbs)(SEMC: ADP006130, ADP006150, females), Río Napo, Sacha Lodge, Pilchicocha, 0.472°S, 76.459°W, 228m, 3-16 August 1994, P Hibbs, SEMC, 006138, female paratype) Limoncocha National Biological Reserve, Limoncocha, 0.4069°S, 76.6134°W, 235m, 11 June 1977 (PJ Spangler, D.R Givens)(NMNH: ADP005800, female). **French Guiana**, Cayenne, Mont Itoupe, placette, 3.0220°N, 53.0947°W, 600m, 26 November 2014 (S Brule, PH Dalens, E Poirier)(NMNH: ADP148089, female),16 January 2016 (S Brule, PH Dalens, E Poirier)(NMNH: ADP148830, ADP148831, females), 800m, 2 December 2014 (S Brule, PH Dalens, E Poirier)(NMNH: ADP148201, female); Commune de Roura, Montagne des Chevaux, 4.7127°N, 52.3966°W, 90, 3 January 2010 (S Brule, PH Dalens, E Poirier)(NMNH: ADP128009, female), 3 January 2015 (S Brule, PH Dalens, E Poirier)(NMNH: ADP148090, female), 1 August 2015 (S Brule, PH Dalens, E Poirier)(NMNH: ADP148101, male), 15 km west, Regina, Petit Montagne Tortue, 4.3204°N, 52.2404°W, 94m, 10 June 2010 (G Lamarre)(NMNH: ADP130780, male), Region de Saul, Commune de Saul, Belvedere de Saul, 3.6223°N, 53.2159°W, 283-325m, 5 February 2010 (S Brule, PH Dalens, E Poirier)(NMNH: ADP130778, female), 9 September 2010 (S Brule, PH Dalens, E Poirier)(NMNH: ADP130777, female), 14 February 2011 (S Brule, PH Dalens, E Poirier)(NMNH: ADP134141, male), 11 December 2012 (S Brule, PH Dalens, E Poirier)(NMNH: ADP134138, male), 16 January 2013 (S Brule, PH Dalens, E Poirier)(NMNH: ADP134136, male, ADP134139, female), 6 February 2013 (S Brule, PH Dalens, E Poirier)(NMNH: ADP134140, female) Region de Saul, Commune de Saul, Belvedere de Saul (point de vue), 3.6223°N, 53.2159°W, 283-325m, 19 August 2010 (S Brule, PH Dalens, E Poirier)(NMNH: ADP130779, female), 7 March 2011 (S Brule, PH Dalens, E Poirier)(NMNH: ADP135284, female), 11 December 2012 (S Brule, PH Dalens, E Poirier)(NMNH: ADP134137, female), Camp Inselberg, Nouragues, Commune de Regina, 4.0334°N, 52.6786°W, 411m, 5 September 2013 (S Brule, PH Dalens, E Poirier)(NMNH: ADP147737, female). **Perú**, Junin, Satipo, 11.265°S, 74.634°W, 865m, July 1941 (A. Maller)(AMNH: ADP007287, male paratype); Loreto, Rio Napo, ACEER, Rio Sucusari, 3.2601°S, 72.9161°W, 100m, 3 June 1992 (TL Erwin, E Pfuno S, F Pfuno S)(NMNH: ADP052156, male, ADP052134, female), 15 June 1992 (TL Erwin, E Pfuno S, F Pfuno S)(NMNH: ADP052729, female), 11 June 1992 (TL Erwin, E Pfuno S, F Pfuno S)(NMNH: ADP051235, male, ADP052671, female), 15 June 1992 (TL Erwin, E Pfuno S, F Pfuno S)(NMNH: ADP052704, ADP052730, females), ACEER-Explonapo Camp, Rio Sucusari, 3.2601°S, 72.9161°W, 100m, 3 June 1992 (TL Erwin, E Pfuno S, F Pfuno S)(NMNH: ADP052134, female), 6 June 1992 (TL Erwin, E Pfuno S, F Pfuno S)(NMNH: ADP053140, male), Pacaya-Samiria National Reserve, Río Samiria, Cocha Shinguito, 5.1775°S, 76.6556°W, 112m, 26 May 1990 (TL Erwin, et al.)(NMNH: ADP067108, male, ADP067084, female), 27 May 1990 (D. Silva)(NMNH: ADP007573, female), 20 June 1990 (TL Erwin, et al.)(NMNH: ADP092866, male, ADP066883, ADP066937, females), 27 August 1991 (TL Erwin, MG Pogue, et al.)(NMNH: ADP050386, female), Río Samiria (South Branch), Camp Terry, 5.6951°S, 75.2243°W, 129m, 14 May 1990 (TL Erwin)(NMNH: ADP093061, male, ADP093060, female), 1 km E Hamburgo, Boca del Ingles Camp, 0.5226°S, 75.1192°W, 125m, 20 August 1991 (TL Erwin, MG Pogue)(NMNH: ADP070664, female), 1.5 km N Teniente Lopez, 2.3566°S, 76.0692°W, 210-240m, 22 July 1993 (R Leschen)(SEMC: ADP007288, female), nr. Iquitos, Porvenir, 3.8918°S, 73.5603°W, 24 June 2011 (G. Lamarre)(NMNH: ADP135887, ADP135889, males), nr. Requena, Jenaro Herrera, 4.900°S, 73.6303°W, 1 August 2011 (G. Lamarre)(NMNH: ADP135885, male); Madre de Dios, Reserva Nacional Tambopata, 30 km (air) SW, Pto. Maldonado, Explorer’s Inn, 12.8364°S, 69.2936°W, 209m, 11-15 November 1979 (JB Heppner, et al.)(NMNH: ADP007286, female), 28 February 1984 (TL Erwin, et al.)(NMNH: ADP007269, male, ADP007270, female), 9 March 1984 (TL Erwin, et al.)(NMNH: ADP007271, male), 4 May 1984 (TL Erwin, et al.)(NMNH: ADP007265, female), 10 May 1984 (TL Erwin, et al.)(NMNH: ADP007248, male), 6 September 1984 (TL Erwin, et al.)(NMNH: ADP007267, female), 10 September 1984 (TL Erwin, et al.)(NMNH: ADP007249, female), Manu Reserved Zone, Río Manu, BIOLAT Biological Station, Pakitza, 11.9446°S, 71.2831°W, 356m, 7 September 1988 (TL Erwin (NMNH: ADP007488, male), 2 September 1989 (TL Erwin, BD Farrell)(NMNH: ADP007487, male), 18 September 1988 (TL Erwin, BD Farrell)(NMNH: ADP007489, male), 19-23 September 1989 (N Adams)(NMNH: ADP007508, female), 22 September 1989 (TL Erwin (NMNH: ADP007446, female), 11 October 1989 (TL Erwin, GP Servat)(NMNH: ADP007485, female), 20 September 1991 (TL Erwin)(NMNH: ADP007403, male, ADP007418, female), 21 September 1991 (TL Erwin, et al.)(NMNH: ADP007464, female), 22 September 1991 (TL Erwin (NMNH: ADP007463, ADP007469, males), 26 September 1991 (TL Erwin (NMNH: ADP007381, ADP007335, males), 28 September 1991 (TL Erwin, MG Pogue)(NMNH: ADP007422, male), 30 September 1991 (TL Erwin (NMNH: ADP007424, ADP007309, ADP007330, ADP007396, ADP007400, ADP007425, males), 2 October 1991 (TL Erwin, MG Pogue)(NMNH: ADP007397, female), 4 October 1991 (TL Erwin, MG Pogue)(NMNH: ADP007462, male), 16 October 1991 (TL Erwin, MG Pogue)(NMNH: ADP007419, ADP007420, males), 13-18 February 1992 (B Brown, D Feener)(NMNH: ADP007510, female), 4-9 March 1992, (B Brown, D Feener)(NMNH: ADP007509, female), 22 June 1993 (TL Erwin, F Pfuno S)(NMNH: ADP007465, ADP007468, males), 23 June 1993 (TL Erwin, F. Pfuno S)(NMNH: ADP007491, male, ADP007490, ADP007506, ADP007467, females). **Suriname**, Brokopondo, Phedra, 5.232°N, 55.050°W, 8m, 1-7 November 1964 (DC Geijskes)(NBCL: ADP005804, male),

###### Geographic distribution.

(Fig. 11). This species is currently known from the type locality in the Amazonian lowlands near Tefé, Brazil, and in Brazil – Amazonas, Pará, Goias; Colombia; Ecuador; French Guiana; Perú; Suriname.

###### Notes.

Reichardt (1971, 1973) reported the following additional specimens that we did not see: **Brazil** – Amazonas: Manaus (13 exs. MZSP), Maturacá, alto Rio Cauaburi (2 exs. MZSP), São Paulo de Olivença (1 ex. MZSP), Tefé (2 exs. MNHP), Titirico (1 ex. FAUCV); Pará: Belém (1 ex. MNHP, 1 ex. MZSP), Icoraci (l ex. MNHP). Goias: Jatar (1 ex. MNHP), Rio Verde (1 ex. MNHP). **Perú** – Loreto: Chambiruyaca, near Yurimaguas (1 ex. MNHP), Pebas (2 exs. MNHP).

##### Hyboptera biolat

Taxon classificationAnimaliaColeoptera Carabidae

Erwin & Henry sp. n.

http://zoobank.org/896D1B12-7939-4277-8C55-A975042F72D9

Biolat humps-backed beetle Figs 1B , 5B , 11

###### Holotype.

Male. **Perú**, Madre de Dios, Manu Reserved Zone, Río Manu, BIOLAT Biological Station, Pakitza, 11.9446°S, 71.2831°W, 356m, 20 September 1991 (TL Erwin)(NMNH: ADP007447).

###### Derivation of specific epithet.

The specific epithet, *biolat*, is used as a noun in apposition based on the acronym of the Smithsonian Institution’s past Program “Biodiversity in Latin America” (BIOLAT) which sought to field-train young Latin American biology students in biodiversity techniques and did so for over 200 of them between 1987 and 1991 in Perú and Bolivia. These beetles were collected under the auspices of the BIOLAT Program.

###### Proposed English vernacular name.

Biolat humps-backed beetle.

###### Diagnosis.

With the attributes of the genus and *angulicollis* species group as described above and adults with patches of bright metallic green para-medially on pronotum, elytra entirely with metallic green patches, and venter with meso- and metathorax mostly infuscated.

###### Description.

(Fig. 1B, 5B). *Size*: See Appendix 1. Length (SBL) short for genus, ABL = 3.64–4.17 mm, SBL = 3.16–3.74 mm, TW = 1.68–2.33 mm.

*Color: See* diagnosis, above.

*Luster*: Metallic highlights, partially iridescent.

*Microsculpture*: Mostly isodiametric or slightly stretched, shallowly impressed, cells somewhat more stretched around elytral tubercles.

*Head*: Rugae moderately coarse, mostly transverse. Eye very large, sub-hemispheric, evenly rounded anteriorly, subtly more prolonged posteriorly. Antenna short, barely reaching humerus. Labrum rectangulate, shallowly bilobed, anterior margin slightly emarginate. Neck finely and transversely rugose.

*Prothorax*: Pronotum moderately broad, disc centrally planar with dense transverse rugae. Lateral margins moderately explanate and obtusely angulate medially then straight to narrowly obtuse hind angle, base medially produced and rounded posteriorly.

*Pterothorax*: Normal for Agrina, fully winged. Elytron interval 3 with 7(8), and interval 5 with 5 (4) discal unisetiferous tubercles, other intervals moderately convex, side margin moderately explanate laterally only at middle third. Elytron broad and moderately short, moderately narrower than the pronotum at the broadest part, apex truncate, slightly rounded with distal corner broadly and obtusely rounded, disc not significantly convex, basal third slightly depressed. All interneurs well-impressed.

*Legs*: Femur dorso-ventrally moderately depressed, tibia coequal in length, more depressed; tarsus less than half the length of the tibia, fourth tarsomere markedly bilobed and with tarsal pad of setae.

*Abdomen*: Sparsely setiferous; normal ambulatory setae on sterna 3–5; female with two pairs of ambulatory setae on sternum 6, medial pair of setae less than the length of lateral pair; males with only the outer pair of longer setae on sternum 6.

*Male genitalia*: Phallus (Fig. 5B) with ostium of 1/5 its length, catopic, apex moderately long, narrowly rounded, narrow in dorsal aspect; endophallus with flagellum (apex extruded in illustration), flagellum not barbed. Parameres asymmetric, right very small, left larger.

*Female genitalia*: Not investigated, likely similar to that of *H. lucida* (Fig. 8).

###### Dispersal potential.

These beetles are macropterous and probably capable of flight. They are moderately swift and agile runners. Specimens have been acquired using insecticidal fogging methods.

###### Way of life.

Adults are common in the lowlands (356 m.a.s.l.) and appear to be generalists in a variety of rainforest biotopes including terra firme and upper floodplain forests. In these forests, they are commonly found in suspended dry leaves in *Guadua weberbaueri* Pilg. bamboo patches and among *Astrocaryum chambira* Burret palm dry leaf-skirts. Adults have been obtained in September–October; hence, they are active in the late dry and early rainy seasons.

###### Other specimens examined.

**Perú**, Madre de Dios, Manu Reserved Zone, Río Manu, BIOLAT Biological Station, Pakitza, 11.9446°S, 71.2831°W, 356m, 4 October 1989 (TL Erwin)(NMNH, ADP007486, male paratype), 10 October 1991 (TL Erwin, MG Pogue)(NMNH: ADP007398, female paratype), 11 October 1991 (TL Erwin, MG Pogue)(NMNH: ADP007353, ADP007354, ADP007356, ADP007443, male paratypes, ADP007355 ADP007399, female paratypes), 14 October 1991 (TL Erwin, MG Pogue)(NMNH: ADP007336, male paratype), 16 October 1991 (TL Erwin)(NMNH: ADP007331, male paratype), 23 September 1991 (TL Erwin)(NMNH: ADP007466, male paratype), 28 September 1991 (TL Erwin, MG Pogue)(NMNH: ADP007315, male paratype), 9 October 1991 (TL Erwin, MG Pogue)(NMNH: ADP007311, male paratype).

###### Geographic distribution

(Fig. 11). This species is currently known only from the type locality in Perú in the Río Manu watershed at the Pakitza Vigilante Post where the BIOLAT Biological Station operated from 1987 to 1992.

###### Notes.

The holotype will be deposited in UNMSM and is currently held in trust until the completion of studies at NMNH. Specimen ADP007443 is missing its fore body.

##### Hyboptera vestiverdis

Taxon classificationAnimaliaColeoptera Carabidae

Henry & Erwin sp. n.

http://zoobank.org/54DF5437-5E03-4BA5-AE7F-8352ED2FDA11

Leprechaun humps-backed beetle Figs 2A , 5C , 11

###### Holotype.

Male. **Perú**, Madre de Dios, Manu Reserved Zone, Río Manu, BIOLAT Biological Station, Pakitza, 11.9446°S, 71.2831°W, 356m, 16 October 1991 (TL Erwin, MG Pogue)(NMNH: ADP007421).

###### Derivation of specific epithet.

The species epithet ‘‘vestiverdis” is a Latinized feminine noun meaning green vest, referring to the two lobes of color on the pronotal disc on individuals of this species with central line of pale color bisecting the pattern and appearing as an open vest.

###### Proposed English vernacular name.

Leprechaun humps-backed beetle.

###### Diagnosis.

With the attributes of the genus and *angulicollis* species group as described above and adults with only dark slightly-metallic markings on the pronotal disc, elytra olivaceous, markedly shiny, some individuals with hint of metallic green near basal margin, narrow, with slightly arcuate lateral margin.

###### Description.

(Figs 2A, 5C) *Size*: See Appendix 1. Length (SBL) short for genus, ABL = 3.46–4.62 mm, SBL = 3.14–3.99 mm, TW = 1.83–2.55 mm.

*Color: See* diagnosis, above.

*Luster*: Very shiny elytra, not iridescent.

*Microsculpture*: Mostly isodiametric and slightly stretched, shallowly impressed, cells somewhat more stretched around elytral tubercles.

*Head*: Rugae moderately coarse, mostly transverse, somewhat curved on occiput. Eye very large, nearly perfectly hemispheric, evenly rounded. Antenna short, barely reaching humerus. Labrum rectangulate, shallowly bilobed, anterior margin slightly emarginate. Neck finely transversely rugose.

*Prothorax*: Pronotum markedly broad, disc planar, with dense transverse rugae. Lateral margins broadly explanate and obtusely angulate medially, then markedly arcuate to obtuse hind angle, base medially produced and rounded posteriorly.

*Pterothorax*: Normal for Agrina, fully winged. Elytron intervals 3 with 7 and 5 with 5(6) elongate unisetiferous tubercles, other intervals moderately convex, side margin broadly explanate laterally only at middle third. Elytron broad and short, slightly narrower than the pronotum at the broadest part, apex truncate, slightly rounded with distal corner broadly and obtusely rounded, disc not significantly convex, basal third slightly depressed. All interneurs well-impressed.

*Legs*: Femur dorso-ventrally moderately depressed, tibia coequal in length, more depressed; tarsus less than half the length of the tibia, fourth tarsomere markedly bilobed and with tarsal pad of setae.

*Abdomen*: Sparsely setiferous; normal ambulatory setae on sterna 3–5; female unknown; males with only the lateral pair of longer setae on sternum 6.

*Male genitalia*: Phallus (Fig. 5C) with ostium of 1/5 its length, catopic, apex moderately long, narrowly rounded; endophallus with flagellum (mid-part obvious in illustration), flagellum not barbed. Parameres asymmetric, right very small, left larger.

*Female genitalia*: Not investigated, likely similar to that of *H. lucida* (Fig. 8).

###### Dispersal potential.

These beetles are macropterous and capable of flight. They are moderately swift and agile runners. Specimens have been acquired in Malaise traps and using insecticidal fogging methods.

###### Way of life.

Adults are common in the lowlands (116 to 356 m.a.s.l.) and appear to be generalists in a variety of rainforest biotopes including terra firme and várzea. In these forests, they are commonly found in big trees with vines and epiphytes, in suspended dry leaves, in dry *Scheelea* sp. and *Astrocaryum chambira* Burret palm frond skirts, in dry leaves scattered in vine tangles, and in dry leaves of the bamboo *Guadua weberbaueri* Pilg. Individuals can be found in January-February, May-July, and September-October, in both the rainy and dry seasons. Member of this species have been recorded from the canopy of the following tree species using insecticidal fogging techniques: *Grias neuberthii*; *Eschweilera coriacea* cf.; *Icicopsis* sp. ; *Pentaplaris huaoranica* sp. nov. *Rhodostemonodaphne kunthiana* cf.; *Nectandra* sp.; *Iriartea deltoidea*; *Protium sagotianum* cf.; *Talisia* bitter; *Mouriri guapira*; *Eriotheca globosa* cf.; *Sloane* sp., *Matisia malacocalyx* cf.; *Tabebuia moby*; Pourouma bicolor ssp. bicolor cf.; *Alchornea triplinervia* cf.?; *Naucleopsis krukovii* cf.; *Hyeronima oblonga*; *Brownea grandiceps* cf.; *Virola decorticans* cf.; Pouteria cuspidata ssp. robusta cf.; *Duguetia surinamensis* cf.; *Trichilia rubra* cf.; *Coccoloba densifrons* cf. *Eschweilera coriacea* cf.; *Virola obovata*; Brosimum utile ssp. ovatifolium cf.; *Pseudolmedia laevis*; *Patinoa paraensis*/*sphaerocarpa* cf.; *Matisia bracteolosa* stellate; *Simaba guianensis* cf.; *Trichilia elsae* cf.; *Icicopsis* sp. nov.

###### Other specimens examined.

**Ecuador**, Orellana, Reserva Ethnica Huaorani, 39 km S Pompeya, Estación Científica Yasuní – Onkone Gare Camp, Erwin Piraña Plot, transect 2, station 10, 0.6581°S, 76.4513°W, 220-250m, 4 October 1995 (TL Erwin, et al.)(NMNH: ADP135922, female paratype), Erwin Piraña Plot, transect 3, station 1, 0.6575°S, 76.4505°W, 220-250m, 1 July 1995 (TL Erwin, et al.)(NMNH: ADP135976, male paratype), Erwin Piraña Plot, transect 3, station 9, 0.6575°S, 76.4505°W, 220-250m, 21 January 2006 (TL Erwin, et al.)(NMNH: ADP133860, female paratype), Erwin Piraña Plot, transect 4, station 3, 0.6570°S, 76.4498°W, 220-250m, 11 February 1995 (TL Erwin, et al.)(NMNH: ADP137321, male paratype), Erwin Piraña Plot, transect 4, station 3, 0.6570°S, 76.4498°W, 220-250m, 11 February 1995 (TL Erwin, et al.)(NMNH: ADP135848, female paratype), Erwin Piraña Plot, transect 4, station 3, 0.6570°S, 76.4498°W, 220-250m, 11 February 1995 (TL Erwin, et al.)(NMNH: ADP135908, female paratype), Erwin Piraña Plot, transect 4, station 3, 0.6570°S, 76.4498°W, 220-250m, 16 January 1994 (TL Erwin, et al.)(NMNH: ADP135934, female paratype), Erwin Piraña Plot, transect 5, station 10, 0.6566°S, 76.4490°W, 220-250m, 6 October 1996 (TL Erwin, et al.)(NMNH: ADP135896, female paratype), Erwin Piraña Plot, transect 5, station 5, 0.6575°S, 76.4505°W, 220-250m, 6 January 1995 (TL Erwin, et al.)(NMNH: ADP135962, female paratype), Erwin Piraña Plot, transect 6, station 10, 0.6561°S, 76.4483°W, 220-250m, 2 October 1996 (TL Erwin, et al.)(NMNH: ADP135932, female paratype), Erwin Piraña Plot, transect 6, station 3, 0.6561°S, 76.4481°W, 220-250m, 6 October 1995 (TL Erwin, et al.)(NMNH: ADP137333, male paratype), Erwin Piraña Plot, transect 8, station 10, 0.6551°S, 76.4403°W, 220-250m, 3 October 1996 (TL Erwin, et al.)(NMNH: ADP137371, male paratype), Erwin Piraña Plot, transect 8, station 8, 0.6551°S, 76.4403°W, 220-250m, 8 February 1996 (TL Erwin, et al.)(NMNH: ADP137381, male paratype), Erwin Piraña Plot, transect 9, station 7, 0.6545°S, 76.4460°W, 220-250m, 8 October 1995 (TL Erwin, et al.)(NMNH: ADP135986, female paratype); Yasuni National Park (edge), 95.43 km E (heading 101.46°) Coca, Tiputini Biodiversity Station, Erwin Harpia Plot, transect 1, station 6, 0.6342°S, 76.1443°W, 214m, 2 October 2000 (TL Erwin, et al.)(NMNH: ADP139098, male paratype), Erwin Harpia Plot, transect 5, station 8, 0.6309°S, 76.1443°W, 220-250m, 1 July 1998 (TL Erwin, et al.)(NMNH: ADP135870, female paratype), Erwin Harpia Plot, transect 7, station 6, 0.6287°S, 76.1443°W, 203m, 22 October 1998 (TL Erwin, et al.)(NMNH: ADP135855, male paratype), Erwin Harpia Plot, transect 7, station 9, 0.6287°S, 76.1443°W, 203m, 29 September 2000 (TL Erwin, et al.)(NMNH: ADP139138, male paratype); Sucumbíos, Río Napo, Sacha Lodge, Pilchicocha, 0.472°S, 76.459°W, 228m, 12-22 February 1994 (P Hibbs)(SEMC: ADP006133, male paratype). **Perú**, Loreto, Pacaya-Samiria National Reserve, Rio Samiria, Pithecia, 5.2116 S, 74.6991 W, 116m, 9 May 1990 (TL Erwin, GP Servat, et al.)(NMNH: ADP007293, male paratype), Boca del Ingles Camp, Hamburgo, 5.6951°S, 75.2243°W, 150m, 10 May 1990 (TL Erwin, et al.)(NMNH: ADP007292, male paratype, ADP007574, female paratype); Madre de Dios, Manu Reserved Zone, Río Manu, BIOLAT Biological Station, Pakitza, 11.9446°S, 71.2831°W, 356m, 11 October 1991 (TL Erwin, MG Pogue)(NMNH: ADP005780, male paratype), 11 October 1991 (TL Erwin)(NMNH: ADP007314, ADP007359, ADP007374, ADP007375, ADP007376, ADP007377, ADP007378, ADP007379, ADP007380, male paratypes, ADP007358, ADP007357, female paratypes), 14 October 1991 (TL Erwin, MG Pogue, NMNH: ADP007333, ADP007334, ADP007337, ADP007352, male paratypes), 16 October 1991 (TL Erwin)(NMNH: ADP007332, male paratype), (TL Erwin, MG Pogue)(NMNH: ADP007402, male paratype, ADP007313, ADP007401, female paratypes), 22 June 1993 (TL Erwin, F Pfuno S)(NMNH: ADP007484, female paratype), 23 June 1993 (TL Erwin, F Pfuno S)(NMNH: ADP007507, female paratype), 9 September 1988 (TL Erwin)(NMNH: ADP007445, male paratype), 30 September 1991 (TL Erwin, MG Pogue)(NMNH: ADP007423, male paratype), 9 October 1991 (TL Erwin, MG Pogue)(NMNH: ADP007442, male paratype, ADP007308, ADP007310, ADP007312, ADP007440, ADP007441, female paratypes); Reserva Nacional Tambopata, 30km (air) SW Pto. Maldonado, 12.8364°S, 69.2936°W, 209m, 28 February 1984 (TL Erwin, et al.)(NMNH: ADP007268, male paratype), 4 May 1984 (TL Erwin, et al.)(NMNH: ADP007266, male paratype).

###### Geographic distribution

(Fig. 11). This species is currently known from the western Amazon Basin in lowland Ecuador and Perú.

###### Notes.

The holotype will be deposited in UNMSM and is currently held in trust until the completion of studies at NMNH.

##### Hyboptera scheelea

Taxon classificationAnimaliaColeoptera Carabidae

Erwin & Henry sp. n.

http://zoobank.org/6F9E1741-7310-4CB0-843F-0771EE17059F

Palm-frond humps-backed beetle Figs 2B , 11

###### Holotype.

Female. **Perú**, Loreto, Pacaya-Samiria National Reserve, Río Samiria (South Branch), Camp Terry, 5.6951°S, 75.2243°W, 129m, 14 May 1990 (TL Erwin)(NMNH: ADP007575).

###### Derivation of specific epithet.

The species epithet “scheelea” is used as a noun in apposition which is based on the genus of palm upon which the holotype was found.

###### Proposed English vernacular name.

Palm-frond humps-backed beetle.

###### Diagnosis.

With the attributes of the genus and *angulicollis* species group as described above and adults without pronotal markings, pronotal surface rufotestaceous, elytron blackish-blue with metallic green highlights across the humeri and green points at some larger tubercles with lateral margin narrowly testaceous to latero-apical corner but not reaching sutural apex. Apical abdominal tergite mostly infuscated with narrow median testaceous stripe. Size smaller than *H. shasta* adults.

###### Description.

(Fig. 2B). *Size*: See Appendix 1. Length (SBL) short for genus, ABL = 4.54 mm, SBL = 3.68 mm, TW = 2.10 mm.

*Color: See* diagnosis, above.

*Luster*: Metallic highlights, partially iridescent.

*Microsculpture*: Mostly isodiametric, shallowly impressed, cells somewhat stretched around elytral tubercles.

*Head*: Rugae moderately coarse, mostly transverse. Eye very large, sub-hemispheric, evenly rounded anteriorly, subtly more prolonged posteriorly. Antenna short, barely reaching humerus. Labrum rectangulate, shallowly bilobed, anterior margin slightly emarginate. Neck finely and transversely rugose.

*Prothorax*: Pronotum markedly broad, disc centrally depressed with dense transverse rugae. Lateral margins broadly explanate and obtusely angulate medially then moderately arcuate to obtuse hind angle, base medially produced and rounded posteriorly.

*Pterothorax*: Normal for Agrina, fully winged. Elytron intervals 3 and 5 each with (4)5 discal unisetiferous tubercles, interval 3 with one such tubercle near apex, other intervals moderately convex, side margin broadly explanate laterally only at middle third. Elytron broad and short, slightly narrower than the pronotum at the broadest part, apex truncate, slightly rounded with distal corner broadly and obtusely rounded, disc not significantly convex, basal third slightly depressed. All interneurs well-impressed.

*Legs*: Femur dorso-ventrally moderately depressed, tibia coequal in length, more depressed; tarsus less than half the length of the tibia, fourth tarsomere markedly bilobed and with tarsal pad of setae.

*Abdomen*: Sparsely setiferous; normal ambulatory setae on sterna 3–5; female with two pairs of ambulatory setae on sternum 6, medial pair of setae less than the length of lateral pair; males unknown.

*Male genitalia*: Unknown.

*Female genitalia*: Not investigated, likely similar to that of *H. lucida* (Fig. 8).

###### Dispersal potential.

These beetles are macropterous and probably capable of flight. They are moderately swift and agile runners. The holotype was acquired by insecticidal fogging of a *Sheelea* palm.

###### Way of life.

The single known adult was found in May in lowlands (129 m.a.s.l.) in the secondary floodplain of igapó forests.

###### Other specimens examined.

None.

###### Geographic distribution

(Fig. 11). This species is currently known only from the type locality in the lowland of Amazonian Perú.

###### Notes.

The holotype will be deposited in UNMSM and is currently held in trust until the completion of studies at NMNH.

##### Hyboptera shasta

Taxon classificationAnimaliaColeoptera Carabidae

Erwin sp. n.

http://zoobank.org/F618555F-D7D8-43BA-B762-2E362948D342

Shasta’s humps-backed beetle Figs 3A , 11

###### Holotype.

Male. **Brazil**, Amazonas, north of Manaus on Amazonas 010 at Km 26, Reserva Ducke, 2.918°S, 59.971°W, 70m, 4 July 1978 (J Arias)(NMNH: ADP135875).

###### Derivation of specific epithet.

The species epithet “shasta” is an eponym based on the first name of the coauthor of this paper and former Intern in the laboratory of the senior author of this paper at the Smithsonian Institution.

###### Proposed English vernacular name.

Shasta’s humps-backed beetle.

###### Diagnosis.

With the attributes of the genus and *angulicollis* species group as described above and adults without pronotal markings, pronotal surface rufotestaceous, elytron brilliant metallic green throughout with lateral margin testaceous to latero-apical corner, not quite reaching sutural apex; apical abdominal tergite mostly infuscated with narrow median testaceous stripe; size larger than *H. scheelea* adults.

###### Description.

(Fig. 3A). *Size*: See Appendix 1. Length (SBL) medium for genus, ABL = 4.82 mm, SBL = 3.86 mm, TW = 2.17 mm.

*Color: See* diagnosis, above.

*Luster*: Very shiny elytra, substantially iridescent.

*Microsculpture*: Mostly isodiametric and slightly stretched, shallowly impressed, cells somewhat more stretched around elytral tubercles.

*Head*: Rugae moderately coarse, mostly transverse. Eye very large, nearly perfectly hemispheric, evenly rounded. Antenna short, barely reaching humerus. Labrum rectangulate, shallowly bilobed, anterior margin slightly emarginate. Neck finely transversely rugose.

*Prothorax*: Pronotum markedly broad, disc not centrally depressed, with dense transverse rugae. Lateral margins broadly explanate and obtusely angulate medially, then moderately arcuate to obtuse hind angle, base medially produced and rounded posteriorly.

*Pterothorax*: Normal for Agrina, fully winged. Elytron intervals 3 with 7 and 5 with 3/4 elongate unisetiferous tubercles, other intervals moderately convex, side margin broadly explanate laterally only at middle third. Elytron broad and short, slightly narrower than the pronotum at the broadest part, apex truncate, slightly rounded with distal corner broadly and obtusely rounded, disc not significantly convex, basal third slightly depressed. All interneurs well-impressed.

*Legs*: Femur dorso-ventrally moderately depressed, tibia coequal in length, more depressed; tarsus less than half the length of the tibia, fourth tarsomere markedly bilobed and with tarsal pad of setae.

*Abdomen*: Sparsely setiferous; normal ambulatory setae on sterna 3–5; female unknown; males with only the lateral pair of longer setae on sternum 6.

*Male genitalia*: Not investigated as only the holotype male is available, likely similar to that of *H. angulicollis* (Fig. 5A).

*Female genitalia*: Not investigated, likely similar to that of *H. lucida* (Fig. 8).

###### Dispersal potential.

These beetles are macropterous and capable of flight. They are moderately swift and agile runners. The holotype was acquired in a C.D.C. light trap.

###### Way of life.

The single known adult was found in July in lowlands (70 m.a.s.l.) in the terra firme forests.

###### Other specimens examined.

None.

###### Geographic distribution

(Fig. 11). This species is currently known only from the type locality in lowland Amazonian Brazil.

##### Hyboptera tepui

Taxon classificationAnimaliaColeoptera Carabidae

Erwin & Henry sp. n.

http://zoobank.org/C3DF93E5-F8A5-452F-BDBD-ED2EF3D1F438

Tepui humps-backed beetle Figs 3B , 11

###### Holotype.

Female. **Venezuela**, Amazonas, Cerro de la Neblina, Río Baria Basecamp, 0.837°N, 66.162°W, 138m, 20 February 1985 (PJ Spangler, PM Spangler, et al.)(NMNH: ADP007576).

###### Derivation of specific epithet.

The species epithet “tepui” is used as a noun in apposition and is based on the type of Venezuelan flat-topped upland near which the holotype was found.

###### Proposed English vernacular name.

Tepui humps-backed beetle.

###### Diagnosis.

With the attributes of the genus and *angulicollis* species group as described above and adults without pronotal markings, pronotal surface rufotestaceous. Elytron with lateral margin broadly testaceous from humerus to sutural apex; apical abdominal tergite testaceous with slight infuscation at extreme posterior-lateral corners.

###### Description.

(Fig. 3B). *Size*: See Appendix 1. Length (SBL) long for genus, ABL = 5.16 mm, SBL = 4.29 mm, TW = 2.458 mm.

*Color: See* diagnosis, above.

*Luster*: Metallic green highlights, partially iridescent.

*Microsculpture*: Mostly irregular isodiametric, often stretched, shallowly impressed, cells especially stretched around elytral tubercles.

*Head*: Rugae moderately coarse, mostly transverse. Eye very large, sub-hemispheric, evenly rounded anteriorly, subtly more prolonged posteriorly. Antenna short, barely reaching humerus. Labrum rectangulate, shallowly bilobed, anterior margin slightly emarginate. Neck finely transversely rugose.

*Prothorax*: Pronotum markedly broad, disc with dense transverse rugae. Lateral margins broadly explanate and obtusely angulate medially then moderately arcuate to obtuse hind angle, base medially produced and rounded posteriorly.

*Pterothorax*: Normal for Agrina, fully winged. Elytron intervals 3 and 5 each with 6 discal unisetiferous tubercles, interval 3 with one subtle tubercle near apex, other intervals shallowly to moderately convex, side margin broadly explanate laterally only at middle third. Elytron broad and short, slightly narrower than the pronotum at the broadest part, apex truncate, slightly rounded with distal corner broadly and obtusely rounded, disc not significantly convex, basal third slightly depressed. All interneurs well-impressed.

*Legs*: Femur dorso-ventrally moderately depressed, tibia coequal in length, more depressed; tarsus less than half the length of the tibia, fourth tarsomere markedly bilobed and with tarsal pad of setae.*Abdomen*: Sparsely setiferous; normal ambulatory setae on sterna 3–5; female with two pairs of ambulatory setae on sternum 6, medial pair of setae less than the length of lateral pair; males unknown.

*Male genitalia*: Unknown.

*Female genitalia*: Not investigated, likely similar to that of *H. lucida* (Fig. 8).

###### Dispersal potential.

These beetles are macropterous and capable of flight. They are moderately swift and agile runners. The holotype was acquired at black light in a rainforest clearing.

###### Way of life.

The single known adult was found in February in lowland (138 m.a.s.l.) terra firme forests.

###### Other specimens examined.

None.

###### Geographic distribution

(Fig. 11). This species is currently known only from the type locality in lowland Venezuela.

##### Hyboptera tiputini

Taxon classificationAnimaliaColeoptera Carabidae

Erwin & Henry sp. n.

http://zoobank.org/04C99837-BEFF-4F3E-BE65-69CA3562A4A0

Tiputini humps-backed beetle Figs 4A , 5D , 11

###### Holotype.

Male. **Ecuador**, Orellana, Yasuni National Park (edge), 95.43 km E (heading 101.46°) Coca, Tiputini Biodiversity Station, Erwin Harpia Plot: transect 4, station 4, 0.6316°S, 76.1443°W, 208m, 8 February 1999 (TL Erwin, et al.)(NMNH: ADP135781).

###### Derivation of specific epithet.

The specific epithet, “tiputini” is used as a noun in apposition and is based on the Tiputini Biodiversity Station in the Yasuni area of eastern Ecuador in reference to one of the places in which members of this species are found.

###### Proposed English vernacular name.

Tiputini humps-backed beetle.

###### Diagnosis.

With the attributes of the genus and *angulicollis* species group as described above and adults with patches of bright metallic green para-medially on pronotum, elytra markedly shiny, violaceous, some individuals with hint of metallic green near basal margin, broad, with markedly arcuate lateral margin.

###### Description.

(Figs 4A, 5D). *Size*: See Appendix 1. Length (SBL) long for genus, ABL = 4.21–5.64 mm, SBL = 3.33–4.72 mm, TW = 1.868–2.92 mm.

*Color: See* diagnosis, above.

*Luster*: Matte with a few green reflections, markedly iridescent.

*Microsculpture*: Mostly irregular isodiametric, often stretched, shallowly impressed, cells especially stretched around elytral tubercles.

*Head*: Rugae moderately coarse, transverse. Eye very large, sub-hemispheric, evenly rounded anteriorly, subtly more prolonged posteriorly. Antenna short, barely reaching humerus. Labrum rectangulate, shallowly bilobed, anterior margin slightly emarginate. Neck finely transversely rugose.

*Prothorax*: Pronotum markedly broad, disc medially shallowly depressed along midline, and with dense transverse rugae. Lateral margins broadly explanate and obtusely angulate medially then subtlely arcuate to obtuse hind angle, base medially produced and rounded posteriorly.

*Pterothorax*: Normal for Agrina, fully winged. Elytron interval 3 with 7(8) and interval 5 with 5 unisetiferous tubercles, other intervals shallowly to moderately convex, side margin broadly explanate laterally only at middle third. Elytron broad and short, slightly narrower than the pronotum at the broadest part, apex truncate, slightly rounded with distal corner broadly and obtusely rounded, disc not significantly convex, basal third slightly depressed. All interneurs well-impressed.

*Legs*: Femur dorso-ventrally moderately depressed, tibia coequal in length, more depressed; tarsus less than half the length of the tibia, fourth tarsomere markedly bilobed and with tarsal pad of setae.

*Abdomen*: Sparsely setiferous; normal ambulatory setae on sterna 3–5; female with two pairs of ambulatory setae on sternum 6, medial pair of setae less than the length of lateral pair; males unknown.

*Male genitalia*: Phallus (Fig. 5D) with ostium of 1/5 its length, catopic, apex moderately long, narrowly rounded, broad in dorsal aspect; endophallus with flagellum (mid-part obvious in illustration), flagellum not barbed. Parameres asymmetric, right very small, left larger.

*Female genitalia*: Not investigated, likely similar to that of *H. lucida* (Fig. 8).

###### Dispersal potential.

These beetles are macropterous and probably capable of flight. They are moderately swift and agile runners.

###### Way of life.

Adults are common in the lowlands and lower midlands (20 to 900 m a.s.l.) and appear to be generalists in a variety of rainforest biotopes including terra firme, várzea, and igapó. In these forests, they are commonly found in big trees with vines and epiphytes, in suspended dry leaves, in dry *Astrocaryum chambira* Burret palm frond skirts. Individuals can be found in January-November, in both the rainy and dry seasons. Member of this species have been recorded from the canopy of the following tree species using insecticidal fogging techniques: *Pseudolmedia laevis*; *Iriartea deltoidea*; *Castilla ulei* cf.; *Sorocea steinbachii* cf.; *Matisia malacocalyx* cf. *Swartzia león*; *Eschweilera coriacea* cf.; *Mouriri guapira*; *Trichilia solitudinis*; *Eriotheca globosa* cf.; *Sloanea* 1; *Vismia* weedy; *Tabebuia moby*; Pourouma bicolor ssp. bicolor cf.; *Alchornea triplinervia* cf.?; *Naucleopsis krukovii* cf.; *Hyeronima oblonga*; *Clarisia biflora*; *Pouteria baehniana* cf.; *Inga cuadra*; *Maquira calophylla*; *Brownea grandiceps* cf.; *Talisia* bitter; *Virola decorticans* cf.; Pouteria cuspidata ssp. robusta cf.; *Diospyros sericea*; *Guarea silvatica*; *Scheelea* sp.

###### Other specimens examined.

**Colombia**, Amazonas, PNN Amacayacu, Mocagua, 3.84°S, 70.22°W, 76m, 14-21 August 2000 (A Parente)(IAvH: AvH-E-73764, ADP145179, female paratype), 12-19 March 2000 (A Parente)(IAvH: AvH-E-2971, ADP145201, female paratype), 19-26 June 2000 (A Parente)(IAvH: AvH-E-73760, ADP145205, ADP145213, female paratypes), 2-8 May 2000 (A Parente)(IAvH: AvH-E-73762, ADP145199, female paratype), 29 May - 6 June 2000 (A Parente)(IAvH: AvH-E-73759, ADP145207, female paratype), 3-9 April 2000 (A Parente)(IAvH: AvH-E-73758, ADP145185, female paratype); Chocó, Ensenada de Utría Cocalito, 6.046°N, 77.352°W, 20m, 1 August - 10 October 2000 (S Sarria) (IAvH: IAvH-E-10943, ADP145168, female paratype); Valle del Cauca, PPN Farallones de Cali, 3.527°N, 76.848°W, 650-900m, 1 August - 10 October 2000 (S Sarria) (IAvH: IAvH-E-3531, ADP145212, male paratype, IAvH-E-10942, ADP145208, IAvH-E-40743, ADP145216, female paratypes). **Ecuador**, Orellana, Reserva Ethnica Huaorani, 39 km S Pompeya, Estación Científica Yasuní - Onkone Gare Camp, 0.6551°S, 76.4403°W, 220-250m, 4-14 October 1995 (GE Ball, D.Shpeley)(UASM: ADP135771, male paratype), Erwin Piraña Plot: transect 6, station 9, 0.6561°S, 76.4483°W, 220-250m, 15 January 1994 (TL Erwin, et al.)(NMNH: ADP138483, male paratype), Erwin Piraña Plot: transect 2, station 8, 0.6581°S, 76.4513°W, 220-250m, 20 June 1994 (TL Erwin, et al.)(NMNH: ADP135847, male paratype), Erwin Piraña Plot: transect 4, station 9, 0.6570°S, 76.4498°W, 220-250m, 25 June 1994 (TL Erwin, et al.)(NMNH: ADP135954, male paratype), Erwin Piraña Plot: transect 5, station 9, 0.6566°S, 76.4490°W, 220-250m, 12 February 1995 (TL Erwin, et al.)(NMNH: ADP137319, female paratype), Erwin Piraña Plot: transect 5, station 10, 0.6566°S, 76.4490°W, 220-250m, 6 October 1995 (TL Erwin, et al.)(NMNH: ADP137341, male paratype, ADP137377, female paratype), Erwin Piraña Plot: transect 10, station 1, 0.6540°S, 76.4453°W, 220-250m, 8 October 1995 (TL Erwin, et al.)(NMNH: ADP135950, female paratype), Erwin Piraña Plot: transect 6, station 8, 0.6561°S, 76.4483°W, 220-250m, 7 February 1996 (TL Erwin, et al.)(NMNH: ADP137389, female paratype), Erwin Piraña Plot: transect 4, station 4, 0.6570°S, 76.4498°W, 220-250m, 21 June 1996 (TL Erwin, et al.)(NMNH: ADP137323, female paratype), Erwin Piraña Plot: transect 5, station 5, 0.6566°S, 76.4490°W, 220-250m, 22 June 1996 (TL Erwin, et al.)(NMNH: ADP137738, male paratype, ADP137349, female paratype), Erwin Piraña Plot: transect 1, station 9, 0.6586°S, 76.4521°W, 220-250m, 30 September 1996 (TL Erwin, et al.)(NMNH: ADP137337, male paratype), Erwin Piraña Plot: transect 5, station 10, 0.6566°S, 76.4490°W, 220-250m, 2 October 1996 (TL Erwin, et al.)(NMNH: ADP137750, female paratype), Erwin Piraña Plot: transect 5, station 8, 0.6566°S, 76.4490°W, 220-250m, 6 October 1996 (TL Erwin, et al.)(NMNH: ADP135857, female paratype); Yasuni National Park (edge), 95.43 km E (heading 101.46°) Coca, Tiputini Biodiversity Station, Erwin Harpia Plot: transect 1, station 6, 0.6342°S, 76.1443°W, 218m, 23 October 1998 (TL Erwin, et al.)(NMNH: ADP135902, ADP135874, female paratypes), Erwin Harpia Plot: transect 3, station 9, 0.63327°S, 76.1443°W, 207m, 24 October 1998 (TL Erwin, et al.)(NMNH: ADP135843, female paratype), Erwin Harpia Plot: transect 6, station 9, 0.6295°S, 76.1443°W, 199m, 26 October 1998 (TL Erwin, et al.)(NMNH: ADP135850, male paratype), Erwin Harpia Plot: transect 9, station 8, 0.6269°S, 76.1443°W, 203m, 5 February 1999 (TL Erwin, et al.)(NMNH: ADP135844, male paratype, ADP135853, ADP135898, female paratypes), Erwin Harpia Plot: transect 6, station 7, 0.6295°S, 76.1443°W, 199m, 7 February 1999 (TL Erwin, et al.)(NMNH: ADP135849, male paratype), Erwin Harpia Plot: transect 4, station 4, 0.6316°S, 76.1443°W, 208m, 8 February 1999 (TL Erwin, et al.)(NMNH: ADP135851, ADP135845, male paratypes), Erwin Harpia Plot: transect 6, station 9, 0.6295°S, 76.1443°W, 199m, 25 October 1999 (TL Erwin, et al.)(NMNH: ADP135916, female paratype). **Perú**, Loreto, Pacaya-Samiria National Reserve, 1 km E Hamburgo, Boca del Ingles Camp, 0,5226°S, 75.1192°W, 125m, 10 May 1990 (TL Erwin, et al.)(NMNH: ADP005785, female paratype); Río Samiria (South Branch), Camp Terry, 5.6951°S, 75.2243°W, 129m, 12 May 1990 (TL Erwin, et al.)(NMNH: ADP005786, female paratype); Madre de Dios, Manu Reserved Zone, Río Manu, BIOLAT Biological Station, Pakitza, 11.9446°S, 71.2831°W, 356m, 7 September 1988 (TL Erwin, et al.)(NMNH: ADP005778, ADP005779, male paratypes), 9 September 1988 (TL Erwin, et al.)(NMNH: ADP005763, female paratype), 12 September 1988 (TL Erwin, et al.)(NMNH: ADP005776, male paratype), 15 February 1990 (TL Erwin, GP Servat)(NMNH: ADP005777, male paratype), 21 September 1991 (TL Erwin, et al.)(NMNH: ADP005764, male paratype), 16 October 1991 (TL Erwin, MG Pogue)(NMNH: ADP007444, male paratype), Reserva Nacional Tambopata, 30 km (air) SW Puerto Maldonado, Explorer’s Inn, 12.8364°S, 69.2936°W, 209m, 14 September 1984 (TL Erwin, et al.)(NMNH: ADP005781, female paratype), 25 October 1982 (TL Erwin, et al.)(NMNH: ADP005798, female paratype), 5-6 November 1982 (R Wilkerson)(NMNH: ADP067693, male paratype), 30 April 1984 (TL Erwin, et al.)(NMNH: ADP005784, male paratype), 8 September 1984 (TL Erwin, et al.)(NMNH: ADP005783, female paratype), 14 September 1984 (TL Erwin, et al.)(NMNH: ADP005782, male paratype).

###### Geographic distribution

(Fig. 11). This species is currently known from the type locality and other localities in Ecuador and various localities in Colombia and Perú.

###### Notes.

The holotype will be deposited in Escuela Politécnica Nacional, Quito, Ecuador and is currently held in trust until the completion of studies at NMNH.

##### Hyboptera viridivittis

Taxon classificationAnimaliaColeoptera Carabidae

Chaudoir, 1872

Green-lined humps-backed beetle Figs 4B , 5E , 11

Hyboptera viridivittis Chaudoir, 1872: 164.

###### Lectotype.

(Here designated) Sex unknown. **Brazil**, Rio de Janeiro, Cantagallo (RF Sahlberg)(MNHP).

###### Derivation of specific epithet.

The specific epithet, *viridivittis*, is a feminine Latin adjective referring to the green line pattern of the pronotum.

###### Proposed English vernacular name.

Green-lined humps-backed beetle.

###### Diagnosis.

With the attributes of the genus and *angulicollis* species group as described above and adults with patches of bright metallic green para-medially on pronotum, elytra dark matte black, some individuals with hint of metallic green near basal margin. Venter with gular region, prosternal region, meso- and metathroax, and margins of abdominal sterna infuscated, otherwise rufous.

###### Description.

(Figs 4B, 5E). *Size*: See Appendix 1. Length (SBL) medium for genus, ABL = 4.65–5.27 mm, SBL = 3.76–4.17 mm, TW = 2.18–2.59 mm.

*Color: See* diagnosis, above.

*Luster*: Matte, pronotum and elytra with metallic highlights.

*Microsculpture*: Mostly isodiametric or stretched, shallowly impressed, cells more stretched around elytral tubercles.

*Head*: Rugae moderately coarse, mostly transverse or angulate. Eye large, sub-hemispheric, evenly rounded anteriorly, subtly more prolonged posteriorly. Antenna short, barely reaching humerus. Labrum rectangulate, shallowly bilobed, anterior margin slightly emarginate. Neck finely transversely rugose.

*Prothorax*: Pronotum moderately broad, disc centrally depressed along midline with dense transverse rugae. Lateral margins broadly explanate and obtusely angulate medially then moderately arcuate to obtuse hind angle, base medially produced and rounded posteriorly.

*Pterothorax*: Normal for Agrina, fully winged. Elytron interval 3 with 8 and interval 5 with 6 unisetiferous tubercles, other intervals moderately convex, side margin narrowly explanate laterally, slightly more so medially. Elytron moderately broad and short, slightly narrower than the pronotum at the broadest part, apex truncate, slightly rounded with distal corner broadly and obtusely rounded, disc not significantly convex, basal third slightly depressed. All interneurs well-impressed.

*Legs*: Femur dorso-ventrally moderately depressed, tibia coequal in length, more depressed; tarsus less than half the length of the tibia, fourth tarsomere markedly bilobed and with tarsal pad of setae.

*Abdomen*: Sparsely setiferous; normal ambulatory setae on sterna 3–5; female with two pairs of ambulatory setae on sternum 6, medial pair of setae less than the length of lateral pair; males with only the lateral pair of longer setae.

*Male genitalia*: Phallus (Fig. 5E) with ostium of 1/5 its length, catopic, apex moderately long, narrowly rounded, broad in dorsal aspect; endophallus with flagellum (mid-part obvious in illustration), flagellum not barbed. Parameres asymmetric, right very small, left larger.

*Female genitalia*: Not investigated, likely similar to that of *H. lucida* (Fig. 8).

###### Dispersal potential.

These beetles are macropterous and probably capable of flight. They are moderately swift and agile runners.

###### Way of life.

Adults are found in April-June, the early dry season, in lowlands (75-846 m.a.s.l.) in the Mata Atlântica.

###### Other specimens examined.

**Brazil**, Rio de Janeiro, Rio de Janeiro, 22.9522°S, 43.2109°W, 459m, 1883 (P Germain)(NMNH: ADP136142, female), Santa Catarina, Nova Teutonia, 27.1833°S, 52.3833°W, 300-500m, April 1977 (F Plaumann)(CAS: ADP007617, male), May 1977 (F Plaumann)(CAS: ADP007597, ADP007601, males, ADP007599, ADP007618, females), June 1977 (F Plaumann)(CAS: ADP007600, ADP007616, females).

###### Geographic distribution

(Fig. 11). This species is currently known from the states of Minas Gerais, Rio de Janeiro, Santa Catarina, and São Paulo in the Mata Atlântica of Brazil.

###### Notes.

Reichardt (1971, 1973) reported the following additional specimens that we did not see: **Brazil** – Minas Gerais: no locality (2 exs. MNHP). Rio de Janeiro: Nova Friburgo (l ex. BMNH). Guanabara, (5 exs. BMNH, MNHP, MZSP). São Paulo: Barueri (l ex. MZSP). Santa Catarina: Nova Teutonia (1 ex. BMNH), Corupá (1 ex. MCZ). Additionally, Chaudoir (1872) mentioned he had two specimens; therefore, a lectotype needs to be designated. Reichardt (1973) mistakenly writes he saw the “holotype.” We have chosen the first of Chaudoir’s specimens as the Lectotype (see above).

#### *tuberculata* species group

(recognized by Reichardt 1973)

The most distinctive attribute of species in this group is that the pronotum has discal rugae etched at an angle aimed medio-posteriorly, or somewhat chaotically seemingly without order. Adults of all species have no metallic coloration (except *H. apollonia* with subtle traces only) on the dorsal surface and the general adult size is medium to large for the genus. Male phallus apex short, broadly or narrowly blunt.

##### Hyboptera apollonia

Taxon classificationAnimaliaColeoptera Carabidae

Erwin, 2004

Apollonia’s humps-backed beetle Figs 6A , 10A , 11

Hyboptera apollonia Erwin, 2004: 33.

###### Holotype.

Male. **Panamá**, Colón, 30 km NE Colón, Porto Bello, 9.555°N, 79.653°W, 9m, 23 February 1911)(EA Schwarz)(NMNH: ADP007943).

###### Derivation of specific epithet.

The specific epithet, *apollonia*, is an eponym based on the first name of Michael Corleone’s beautiful young Italian wife in the movie *The Godfather* whose death in a car explosion perpetrated by Mafia competition signifies the useless instantaneous death of so many species when humans put fire to the tropical rain forest in time of drought.

###### Proposed English vernacular name.

Apollonia’s humps-backed beetle.

###### Diagnosis.

With the attributes of the genus and *angulicollis* species group as described above and with only dark non-metallic markings on the pronotal disc; pronotum with discal rugae etched horizontally and linear. Elytra black with paler margin not reaching suture. Mouthparts, appendages, margin of prothorax, venter of head and prothorax, abdominal segments II–V testaceous; meso- and metathorax, and abdominal segment VI infuscated.

###### Description.

(Figs 6A, 10A). *Size*: See Appendix 1. Length (SBL) medium for genus, ABL = 4.19–5.14mm, SBL = 3.53–4.26 mm, TW = 2.09–2.57 mm.

*Color: See* diagnosis, above.

*Luster*: Shiny, no metallic highlights, subtle iridescent around tubercles.

*Microsculpture*: Mostly isodiametric or slightly stretched, well-impressed, cells somewhat stretched around elytral tubercles.

*Head*: Rugae moderately coarse, mostly not arranged. Eye very large, sub-hemispheric, evenly rounded anteriorly, subtly more prolonged posteriorly. Antenna short, barely reaching humerus. Labrum rectangulate, shallowly bilobed, anterior margin slightly emarginate. Neck finely transversely rugose.

*Prothorax*: Pronotum markedly broad, disc centrally slightly depressed with dense transverse rugae. Lateral margins very broadly explanate and obtusely rounded medially then nearly straight to obtuse hind angle, base medially produced and rounded posteriorly.

*Pterothorax*: Normal for Agrina, fully winged. Elytron intervals 3 with 7, and interval 5 with 6 (5) discal unisetiferous tubercles, other intervals moderately convex, side margin moderately explanate laterally only at middle third. Elytron broad and moderately short, moderately narrower than the pronotum at the broadest part, apex truncate, slightly rounded with distal corner broadly and obtusely rounded, disc not significantly convex, basal third slightly depressed. All interneurs well-impressed.

*Legs*: Femur dorso-ventrally moderately depressed, tibia coequal in length, more depressed; tarsus less than half the length of the tibia, fourth tarsomere markedly bilobed and with tarsal pad of setae.

*Abdomen*: Sparsely setiferous; normal ambulatory setae on sterna 3–5; female with two pairs of ambulatory setae on sternum 6, medial pair of setae less than the length of lateral pair; males with only the lateral pair of longer setae on sternum 6.

*Male genitalia*: Phallus (Fig. 10A) with ostium of 1/6 its length, catopic, apex very short, moderately rounded; endophallus with flagellum (base obvious in illustration), flagellum not barbed. Parameres asymmetric, right very small, left larger.

*Female genitalia*: Not investigated, likely similar to that of *H. lucida* (Fig. 8).

###### Dispersal potential.

These beetles are macropterous and capable of flight. They are moderately swift and agile runners. Modern collecting methods have acquired specimens, including insecticidal foggings and malaise traps.

###### Way of life.

An adult of this species was fogged from a tree in the genus *Guarea* at La Selva and another caught in a Malaise trap as part of the ALAS Project. F. Nevermann collected a specimen on a rotten log. The known elevational range of this species is between 9 and 815 m.a.s.l. Adults have been obtained in January–April, and September–October; hence they are active in both the dry and rainy seasons in the lowlands and lower middle altitudes, both on the east and west sides of the Cordillera Central.

###### Other specimens examined.

**Costa Rica**, Heredia, 11 km SE La Virgin, 10.4313°N, 84.0056°W, 450-650m, 11 May 2003 (Proy. ALAS)(INBIO: ADP135881, female), 250-350m, 22 February 2004 (Proy. ALAS)(INBIO: ADP140501, female), 3 km S Pto. Viejo, Estación Biológica La Selva, 10.4313°N, 84.0056°W, 150m, 18 May 1993, (Proy. ALAS)(HESP: ADP102312, female), 13 January 1996 (Proy. ALAS)(INBIO-OET: ADP135879, female); Limón, Rio Reventazon, Ebene, Hamburg Farm, 10.4149°N, 83.7506°W, 50m, 4 October 1928 (F Nevermann)(NMNH: ADP007942, female), 20 April 1932 (F Nevermann)(NMNH: ADP007945, female), P.N. Tortuguero, Tortuguero, Estación Cuatro Esquinas, 10.5338°N, 83.5071°W, sea level, June 1991 (J Solano)(INBIO, 007578, male); Puntarenas, Peninsula de Osa, Rancho Quemado, 8.6790°W, 83.5667°W, 200m, September 1991 (F Quesada)(INBIO: ADP100266, male). **Panamá**, Colón, 30 km NE Colón, Porto Bello, 9.555°N, 79.653°W, 9m, 2-11 March 1911 (EA Schwarz)(NMNH: ADP007944, male), Gamboa, Pipeline Road, 9.124°N, 79.749°W, 37m, 17-22 June 1993 (SW Lingafelter)(SEMC: ADP007577, female); Panamá, Cerro Jefe, 9.2373°N, 79.3549°W, 815m, 20 May 1972 (RT Allen)(NMNH: ADP011167, female).

###### Geographic distribution

(Fig. 11). This species is currently known from the type locality in Panamá and throughout southern Central America north to Costa Rica.

###### Notes.

Adults of this species are unusual in that they subtly bear two attributes similar to species in the *angulicollis* species-group: faint traces of metallic green and a short narrow nubnen-like apex of the phallus, rather than short and broadly blunt. Adults of the *angulicollis* species-group are extensively green and the phallus apex, while narrow is elongate.

##### Hyboptera auxilidora

Taxon classificationAnimaliaColeoptera Carabidae

Erwin, 2004

Maria’s humps-backed beetle Figs 6B , 11

Hyboptera auxilidora Erwin, 2004: 35.

###### Holotype.

Male. **USA**. Texas, Hidalgo County, nr. Mission, Bentson Rio Grande State Park, 26.176°N, 98.385°W, 38m, 18 July 1981 (WE Steiner)(NMNH: ADP007623).

###### Derivation of specific epithet.

The specific epithet, *auxiliadora*, is an eponym based on the middle name of María Auxiliadora Sanchez, who for many years was responsible for the welfare of participating visiting taxonomists at INBio and its facilities and who made life easy therein while we undertook our studies of the rich Costa Rican fauna and flora.

###### Proposed English vernacular name.

Maria’s humps-backed beetle.

###### Diagnosis.

With the attributes of the genus and *tuberculata* species group as described above and adults with only dark non-metallic markings on the pronotal disc, venter substantially infuscated, pronotum with discal rugae etched at an angle aimed medio-posteriorly, or somewhat chaotically; elytron with sutural margin at apical sixth pale, not black, if brownish not contrasting with background color, and elytra just posterior to scutellum with a V-shaped pale area encompassing the sutures and first intervals. Elytron broad with markedly arcuate lateral margin.

###### Description.

(Fig. 6B). *Size*: See Appendix 1. Length (SBL) long for genus, ABL = 5.06–5.93 mm, SBL = 4.39–4.98 mm, TW = 2.42–3.28 mm.

*Color: See* diagnosis, above.

*Luster*: Shiny, not metallic.

*Microsculpture*: Mostly isodiametric or stretched, shallowly impressed, cells somewhat stretched around elytral tubercles.

*Head*: Rugae moderately coarse, mostly chaotic. Eye markedly large, sub-hemispheric, evenly rounded anteriorly, subtly more prolonged posteriorly. Antenna short, barely reaching humerus. Labrum rectangulate, shallowly bilobed, anterior margin slightly emarginate. Neck transversely rugose.

*Prothorax*: Pronotum markedly broad, disc centrally depressed along midline with dense arcuate rugae directed anteriorly in apical half, posteriorly in posterior half of disc. Lateral margins broadly explanate and subtly produced at lateral seta, but not acute medially then straight to obtuse hind angle, base medially slightly produced and rounded posteriorly.

*Pterothorax*: Normal for Agrina, fully winged. Elytron interval 3 with 10 discal unisetiferous tubercles and interval 5 with 9(8) discal unisetiferous tubercles, interval 3 with one such tubercle near apex, other intervals moderately convex, side margin broadly explanate laterally at middle third. Elytron moderately broad and short, moderately narrower than the pronotum at the broadest part, apex truncate, slightly rounded with distal corner broadly and obtusely rounded, disc not significantly convex, basal third slightly depressed. All interneurs well-impressed.

*Legs*: Femur dorso-ventrally moderately depressed, tibia coequal in length, more depressed; tarsus less than half the length of the tibia, fourth tarsomere markedly bilobed and with tarsal pad of setae.

*Abdomen*: Sparsely setiferous; normal ambulatory setae on sterna 3–5; female with two pairs of ambulatory setae on sternum 6, medial pair of setae less than the length of lateral pair; males with only the lateral pair of longer setae.

*Male genitalia*: Phallus (see fig. 20 in Erwin, 2004) with ostium 1/4 length, catopic and apex short and evenly rounded, endophallus with flagellum, flagellum not barbed. Parameres asymmetric, right very small, left very large.

*Female genitalia*: Not investigated, likely similar to that of *H. lucida* (Fig. 8).

###### Dispersal potential.

These beetles are macropterous and capable in flight. They are moderately swift and agile runners. Specimes have been collected from light traps (white and UV light) and Malaise traps.

###### Way of life.

Adults are found in July in lowlands (10–240 m.a.s.l.) in terra firme forests. The holotype was collected by W. Steiner from under bark of the tree *Celtis levigata* Willd. Vogt collected another individual from under the web tent of a psocid colony (Psocoptera). Adults have been obtained in March, April, May, July, August, and September; hence they are active in both the dry and rainy seasons in the lowlands on both sides of the Cordillera Central.

###### Other specimens examined.

**Costa Rica**, Guanacaste, P.N. Guanacaste, 30 km N Liberia, Finca Jenny, 10.8655°N, 85.5735°W, 240m, 14-21 August 1993 (E Araya)(INBIO: ADP007640, female); Limón, Rio Reventazon, Ebene, Hamburg Farm, 10.4149°N, 83.7506°W, 50m, 6 November 1925 (F Nevermann)(NMNH: ADP007947, female); Puntarenas, P.N. Carara, Estación Quebrada Bonita, 9.7737°N, 84.6122°W, 100m, 17 March - 30 April ---- (P Campos)(INBIO: ADP007639, male). **Honduras**, Atlantida, Tela, 15.719°N, 87.458°W, 85m, 15 May 1995 (R Cave)(NMNH: ADP135859, female). **México**, Veracruz, 3.58 km NE Catemaco, Lake Catemaco, 18.444 N, 95.078 W, 355m, 23 August 1967 (GE Ball, TL Erwin, et al.)(UASM: ADP007621, female). **Panamá**, Canal Zone, Barro Colorado Nature Monument, Barro Colorado Island, Barro Colorado Research Station, Mess Hall, 9.1652°N, 79.8368°W, 70m, 19 February 1975 (TL Erwin, JL Lawrence)(NMNH: ADP027570, male); Cocle, El Valle, 8.603°N, 80.152°W, 829m, 26 May 1983 (WE Steiner)(NMNH: ADP135861, ADP135863, males, ADP135865, ADP005801, females). **USA**, Texas, southeast Hidalgo County, 1 December 1946 (GB Vogt)(NMNH: ADP007638, female), Bentson Rio Grande State Park, Mission, 26.176°N, 98.385°W, 38m, 16 July 1975 (CA Triplehorn)(OSU: ADP007622, female), Sabal Palm Grove Sanctuary, nr. Southmost, 25.8419°N, 97.4247°W, 8m, 1 May 1979 (R.Turnbow)(UASM: ADP007620, male).

###### Geographic distribution

(Fig. 11). This species is currently known only from the type locality and nearby areas in Texas, and from Panamá, and in between those extremes only from México, Honduras, and Costa Rica. It likely is to be found in other Central American countries with further sampling.

##### Hyboptera dilutior

Taxon classificationAnimaliaColeoptera Carabidae

Oberthür, 1884

Oberthür’s humps-backed beetle Figs 7A , 10B , 11

Hyboptera dilutior Oberthür, 1884: 52.

###### Holotype.

Sex unknown. **Brazil**. Amazonas: Tefé (MNHP).

###### Derivation of specific epithet.

The specific epithet, *dilutior*, is an adjective referring to the “washed out” appearance of the color of adults of this species in comparison with those of other species.

###### Proposed English vernacular name.

Oberthür’s humps-backed beetle.

###### Diagnosis.

With the attributes of the genus and *tuberculata* species group as described above and adults with only dark non-metallic markings on the head and pronotal disc, pronotum with discal rugae in basal half etched at an angle aimed medio-posteriorly, or somewhat chaotically. Elytra mostly testaceous with darkly marked tubercles; small medio-apical tubercles also infuscated; with sutural margin at apical sixth infuscated markedly contrasting with testaceous background color. Venter of head and thorax substantially infuscated; abdominal sterna mostly pale with subtle infuscation.

###### Description.

(Figs 7A, 10B). *Size*: See Appendix 1. Length (SBL) long for genus, ABL = 3.92–5.98 mm, SBL = 3.55–4.67 mm, TW = 1.95–3.21 mm.

*Color*: *See* diagnosis, above.

*Luster*: Without any metallic highlights, shiny.

*Microsculpture*: Mostly isodiametric and stretched, shallowly impressed, cells somewhat more stretched around elytral tubercles.

*Head*: Rugae moderately coarse, mostly without patterned arrangement. Eye moderately large, hemispheric, evenly rounded. Antenna short, barely reaching humerus. Labrum rectangulate, shallowly bilobed, anterior margin slightly emarginate. Neck finely transversely rugose.

*Prothorax*: Pronotum markedly broad, disc centrally markedly depressed with coarse angulate rugae. Lateral margins broadly explanate and evenly rounded to obtuse hind angle, base medially produced and rounded posteriorly.

*Pterothorax*: Normal for Agrina, fully winged. Elytron intervals 3 and 5 each with 7 promient discal unisetiferous tubercles, other intervals moderately convex, side margin broadly explanate laterally only at middle third. Elytron broad and short, much narrower in width to that of the pronotum at the broadest part, apex truncate, slightly rounded with distal corner broadly and obtusely rounded, disc not significantly convex, basal third slightly depressed. All interneurs well-impressed.

*Legs*: Femur dorso-ventrally moderately depressed, tibia coequal in length, more depressed; tarsus less than half the length of the tibia, fourth tarsomere markedly bilobed and with tarsal pad of setae.

*Abdomen*: Sparsely setiferous; normal ambulatory setae on sterna 3–5; female with two pairs of ambulatory setae on sternum 6, medial pair of setae less than the length of lateral pair; males with only the lateral pair of longer setae.

*Male genitalia*: Phallus (Fig. 10B) with ostium of 1/6 its length, catopic, apex very short, narrowly pointed, broadly rounded in dorsal aspect; endophallus with flagellum (obvious in illustration), flagellum not barbed. Parameres asymmetric, right very small, left larger.

*Female genitalia*: Not investigated, likely similar to that of *H. lucida* (Fig. 8).

###### Dispersal potential.

These beetles are macropterous and capable of flight. They are moderately swift and agile runners. Adults of this species are attracted to C.D.C. light traps and captured in SLAM Malaise traps.

###### Way of life.

Adults are common in the lowlands (7 to 356 m a.s.l.) and appear to be generalists in a variety of rainforest biotopes including terra firme and várzea. In these forests, they are commonly found in big trees with vines and epiphytes, in suspended dry leaves, in dry *Sheelea* sp. and *Astrocaryum chambira* Burret palm frond skirts. Individuals can be found from January to December, in both the rainy and dry seasons. Member of this species have been recorded from the canopy of the following tree species using insecticidal fogging techniques: *Chrysophyllum argenteum* cf.; *Sterculia colombiana* cf.; *Parkia multijuga* cf.; *Naucleopsis herrerensis* cf.; *Matisia malacocalyx* cf.; *Pseudolmedia laevigata*; *Cecropia herthae*; *Pentagonia spathicalyx* cf.; *Eschweilera coriacea* cf.; *Batocarpus orinocensis* cf.; Zanthoxylum riedelianum ssp. kellermanii cf.; *Cecropia ficifolia*; *Inga capitata*; *Leonia glycicarpa*; *Coussapoa herthae*; Pourouma mollis ssp. triloba cf.; *Pausandra trianae*; *Coussapoa orthoneura* cf.; *Alchornea triplinervia* cf.; *Protium sagotianum* cf.; *Guatteria* sp.; *Meiocarpus*, long petiole; *Oenocarpus bataua*; *Neea* dive-tuberculate; *Semaphyllanthe megistocaula* cf.; Lauraceae redvein; *Siparuna decipiens*; *Trichilia solitudinis*.

###### Other specimens examined.

**Brazil**, Amazonas, Parana do Xiboreninho, 3.2482°S, 59.9791°W, 7m, 7 August 1979 (TL Erwin, J Adis, et al.)(NMNH: ADP006180, female), 40 km SW Manaus, Paraña Costa da Ilha de Curarí, 3.4165°S, 60.2508°W, 17m, 3 August 1979 (TL Erwin, J Adis)(NMNH: ADP005799, male), north of Manaus, on Amazonas 010 at Km 26, Reserva Ducke, 2.918°S, 59.971°W, 70m, 2 May 1978 (J Arias)(NMNH: ADP135826, female), 11 May 1978 (J Arias)(NMNH: ADP135786, male), 1 August 1978 (J Arias)(NMNH: ADP135804, female), 6 September 1978 (J Arias)(NMNH: ADP135792, male), 13 September 1978 (J Arias)(NMNH: ADP135790, ADP135818, females), 20 September 1978 (J Arias)(NMNH: ADP135778, male); Pará, Belém, 1.555°S, 48.429°W, 25m, 25 January 1969 (L O’Brien, CW O’Brien)(NMNH: ADP006178, female), Parque Estadual do Utinga, Belém, 1.406°S, 48.399°W, 21m, 20 January 1977 (N Guimaraes)(NMNH: ADP006179, female); Rondonia, 7 km E Costa Marques, 12.435°S, 64.292°W, 153m, 11-23 April 1987 (T Klein)(NMNH: ADP135883, female). **Ecuador**, Orellana, Reserva Ethnica Huaorani, 39 km S, Pompeya, Estación Científica Yasuní - Onkone Gare Camp, Erwin Piraña Plot: transect 4, station 7, 0.6570°S, 76.4498°W, 220-250m, 16 January 1994 (TL Erwin, et al.)(NMNH: ADP137700, female), Erwin Piraña Plot: transect 5, station 7, 0.6566°S, 76.4490°W, 220-250m, 26 January 1994 (TL Erwin, et al.)(NMNH: ADP135940, male), Erwin Piraña Plot: transect 10, station 10, 0.6540°S, 76.4453°W, 220-250m, 8 February 1995 (TL Erwin, et al.)(NMNH: ADP006175, male), Erwin Piraña Plot: transect 8, station 5, 0.6551°S, 76.4403°W, 220-250m, 30 June 1995 (TL Erwin, et al.)(NMNH: ADP137756, male), Erwin Piraña Plot: transect 1, station 4, 0.6586°S, 76.4521°W, 220-250m, 4 February 1996 (TL Erwin, et al.)(NMNH: ADP137692, female), Erwin Piraña Plot: transect 5, station 7, 0.6566°S, 76.4490°W, 220-250m, 9 February 1996 (TL Erwin, et al.)(NMNH: ADP135974, male), Erwin Piraña Plot: transect 2, station 7, 0.6581°S, 76.4513°W, 220-250m, 30 September 1996 (TL Erwin, et al.)(NMNH: ADP137698, male), Erwin Piraña Plot: transect 6, station 2, 0.6561°S, 76.4483°W, 220-250m, 2 October 1996 (TL Erwin, et al.)(NMNH: ADP135926, female), Erwin Piraña Plot: transect 8, station 5, 0.6551°S, 76.4403°W, 220-250m, 3 October 1996 (TL Erwin, et al.)(NMNH: ADP135930, male), Erwin Piraña Plot: transect 9, station 8, 0.6545°S, 76.4460°W, 220-250m, 4 October 1996 (TL Erwin, et al.)(NMNH: ADP137387, ADP137690, males), Erwin Piraña Plot: transect 3, station 7, 0.6575°S, 76.4505°W, 220-250m, 21 January 2006 (TL Erwin, et al.)(NMNH: ADP133846, male), Yasuni National Park (edge), 95.43 km E (heading 101.46°) Coca, Tiputini Biodiversity Station, Erwin Harpia Plot: transect 10, station 2, 0.6262°S, 76.1443°W, 214m, 5 February 1999 (TL Erwin, et al.)(NMNH: ADP135832, female), Orellana, Yasuni National Park (edge), 95.43 km E (heading 101.46°) Coca, Tiputini Biodiversity Station, Erwin Harpia Plot: transect 3, station 9, 0.6332°S, 76.1443°W, 207m, 30 June 1998 (TL Erwin, et al.)(NMNH: ADP135782, female), Erwin Harpia Plot: transect 10, station 8, 0.6262°S, 76.1443°W, 214m, 21 October 1998 (TL Erwin, et al.)(NMNH: ADP135836, male), Erwin Harpia Plot: transect 10, station 3, 0.6262°S, 76.1443°W, 214m, 5 February 1999 (TL Erwin, et al.)(NMNH: ADP135784, female), Erwin Harpia Plot: transect 6, station 5, 0.6295°S, 76.1443°W, 199m, 7 February 1999 (TL Erwin, et al.)(NMNH: ADP135840, male, ADP135806, female), Erwin Harpia Plot: transect 5, station 6, 0.6332°S, 76.1443°W, 207m, 8 February 1999 (TL Erwin, et al.)(NMNH: ADP135794, female), Erwin Harpia Plot: transect 10, station 4, 0.6262°S, 76.1443°W, 214m, 28 September 2000 (TL Erwin, et al.)(NMNH: ADP139140, male), Erwin Harpia Plot: transect 6, station 1, 0.6304°S, 76.1443°W, 199m, 1 October 2000 (TL Erwin, et al.)(NMNH: ADP139128, ADP139134, ADP139150, males), Erwin Harpia Plot: transect 1, station 3, 0.6342°S, 76.1443°W, 214m, 2 October 2000 (TL Erwin, et al.)(NMNH: ADP139124, male), Erwin Harpia Plot: transect 10, station 5, 0.6262°S, 76.1443°W, 214m, 17 February 2001 (TL Erwin, et al.)(NMNH: ADP139112, male), Erwin Harpia Plot: transect 3, station 1, 0.6332°S, 76.1443°W, 207m, 24 July 2001 (TL Erwin, et al.)(NMNH: ADP139527, female); Sucumbíos, Río Napo, Sacha Lodge, Pilchicocha, 0.472°S, 76.459°W, 228m, 22 February - 4 March 1994 (P Hibbs)(SEMC: ADP006174, male), 14-24 March 1994 (P Hibbs)(SEMC: ADP006173, female), 13-23 April 1994 (P Hibbs)(SEMC: ADP006132, female), 24 March - 3 June 1994 (P Hibbs)(SEMC: ADP007645, female). **French Guiana**, Cayenne, Foret de Maya, Commune Macouria, 4.9535°S, 52.4566°W, 32m, 19 December 2016 (S Brule, PH Dalens, E Poirier)(NMNH: ADP148976, male, Mitaraka, Commune Maripasoula, 2.2723°S, 54.5152°W, 445m, 4 March 2016 (S Brule, PH Dalens, E Poirier)(NMNH: ADP148975, female), Commune de Roura, Montagne des Chevaux, 4.7127°N, 52.3966°W, 90m, 19 June 2010 (S Brule, PH Dalens, E Poirier)(NMNH: ADP135774, male, ADP135788, female), 2 January 2011 (S Brule, PH Dalens, E Poirier)(NMNH: ADP130786, female), 28 March 2011 (S Brule, PH Dalens, E Poirier)(NMNH: ADP135814, male), 14 August 2011 (S Brule, PH Dalens, E Poirier)(NMNH: ADP135802, female), 21 August 2011 (S Brule, PH Dalens, E Poirier)(NMNH: ADP135820, female), 11 September 2011 (S Brule, PH Dalens, E Poirier)(NMNH: ADP135776, ADP135824, females), 25 September 2011 (S Brule, PH Dalens, E Poirier)(NMNH: ADP135770, female), 8 October 2011 (S Brule, PH Dalens, E Poirier)(NMNH: ADP135816, female), 23 October 2011 (S Brule, PH Dalens, E Poirier)(NMNH: ADP140522, male, ADP135772, female), 30 October 2011 (S Brule, PH Dalens, E Poirier)(NMNH: ADP135796, female), 12 November 2011 (S Brule, PH Dalens, E Poirier)(NMNH: ADP135780, female), 11 December 2011 (S Brule, PH Dalens, E Poirier)(NMNH: ADP135830, female), 18 December 2011 (S Brule, PH Dalens, E Poirier)(NMNH: ADP135800, ADP135810, females), 21 December 2011 (S Brule, PH Dalens, E Poirier)(NMNH: ADP135768, ADP135808, females), 24 December 2011 (S Brule, PH Dalens, E Poirier)(NMNH: ADP135798, female), 18 February 2012 (S Brule, PH Dalens, E Poirier)(NMNH: ADP128634, female), 3 November 2012 (S Brule, PH Dalens, E Poirier)(NMNH: ADP135828, male), 3 January 2013 (S Brule, PH Dalens, E Poirier)(NMNH: ADP135822, male), 27 January 2013 (S Brule, PH Dalens, E Poirier)(NMNH: ADP135811, male), 31 March 2013 (S Brule, PH Dalens, E Poirier)(NMNH: ADP135812, female), 20 April 2013 (S Brule, PH Dalens, E Poirier)(NMNH: ADP148093, male), 12 April 2014 (S Brule, PH Dalens, E Poirier)(NMNH: ADP140520, male), 19 July 2014 (S Brule, PH Dalens, E Poirier)(NMNH: ADP140531, male), 27 December 2014 (S Brule, PH Dalens, E Poirier)(NMNH: ADP148092, male), 3 January 2015 (S Brule, PH Dalens, E Poirier)(NMNH: ADP148091, male), 17 October 2015 (S Brule, PH Dalens, E Poirier)(NMNH: ADP148094, male), 2 January 2016 (S Brule, PH Dalens, E Poirier)(NMNH: ADP148095, ADP148096, ADP148097, males), 9 January 2016 (S Brule, PH Dalens, E Poirier)(NMNH: ADP148102, male), 30 April 2016 (S Brule, PH Dalens, E Poirier)(NMNH: ADP148871, male), Commune Matoury, Mont Grand, 4.862°N, 52.355°W, 215m, 17 December 2012 (S Brule, PH Dalens, E Poirier)(NMNH: ADP135801, female), Inselberg, Nouragues, Commune de Regina, Saut Parare, 4.0334°N, 52.6786°W, 51m, 13 October 2010 (S Brule, PH Dalens, E Poirier)(NMNH: ADP128636, female), Camp Inselberg, Nouragues, Commune de Regina, 4.0839°N, 52.6813°W, 411m, 22 November 2012 (S Brule, PH Dalens, E Poirier)(NMNH: ADP147735, female), Region de Saul, Commune de Saul, Belvedere de Saul (point de vue), 3.6223°N, 53.2159°W, 283-325m, 5 February 2010 (S Brule, PH Dalens, E Poirier)(NMNH: ADP130781, female), 5 February 2010 (S Brule, PH Dalens, E Poirier)(NMNH: ADP130782, female), 20 December 2010 (S Brule, PH Dalens, E Poirier)(NMNH: ADP130784, female), 24 January 2011 (S Brule, PH Dalens, E Poirier)(NMNH: ADP130783, female), 4 February 2011 (S Brule, PH Dalens, E Poirier)(NMNH: ADP134146, female), 7 February 2011 (S Brule, PH Dalens, E Poirier)(NMNH: ADP130785, ADP134155, females), 22 March 2011 (S Brule, PH Dalens, E Poirier)(NMNH: ADP134142, ADP134150, males, ADP134148, ADP134149, females), 30 March 2011 (S Brule, PH Dalens, E Poirier)(NMNH: ADP134147, male), 15 June 2011 (S Brule, PH Dalens, E Poirier)(NMNH: ADP134296, female), 11 August 2011 (S Brule, PH Dalens, E Poirier)(NMNH: ADP134154, female), 13 December 2011 (S Brule, PH Dalens, E Poirier)(NMNH: ADP134144, female), 3 October 2012 (S Brule, PH Dalens, E Poirier)(NMNH: ADP134298, male), 11 December 2012 (S Brule, PH Dalens, E Poirier)(NMNH: ADP134153, male, ADP134151, ADP134152, females), 16 January 2013 (S Brule, PH Dalens, E Poirier)(NMNH: ADP134145, female), 27 January 2013 (S Brule, PH Dalens, E Poirier)(NMNH: ADP147733, female), Foret de Maya, Commune Macouria, 4.9552°N, 52.4603°W, 30m, 19 December 2016 (S Brule, PH Dalens, E Poirier)(NMNH: ADP151236, male). **Perú**, Loreto, Pacaya-Samiria National Reserve, Río Samiria, Cocha Shinguito, 5.1775°S, 76.6556°W, 112m, 29 August 1991 (TL Erwin, MG Pogue)(NMNH: ADP051385, ADP051413, males, ADP051412, ADP051415, females), 19 June 1990 (TL Erwin, et al.)(NMNH: ADP094114, ADP094121, males, ADP094067, female), Río Samiria (South Branch), Camp Terry, 5.6951°S, 75.2243°W, 129m, 14 May 1990 (TL Erwin, et al.)(NMNH: ADP007644, female), 16 May 1990 (TL Erwin)(NMNH: ADP094121, ADP094084, females); Madre de Dios, Manu Reserved Zone, Río Manu, BIOLAT Biological Station, Pakitza, 11.9446°S, 71.2831°W, 356m, 14 October 1991 (TL Erwin, MG Pogue)(NMNH: ADP007512, ADP007513, females), 16 October 1991 (TL Erwin, MG Pogue)(NMNH: ADP007530, female), 23 June 1993 (TL Erwin, F. Pfuno)(NMNH: ADP007531, male, ADP007529, ADP007551, females), 28 September 1991 (TL Erwin, MG Pogue)(NMNH: ADP007528, female), 6 October 1991 (TL Erwin, MG Pogue)(NMNH: ADP007511, female), Reserva Nacional Tambopata, 30 km (air) SW Puerto Maldonado, Explorer’s Inn, 12.8364°S, 69.2936°W, 209m, 16 March 1982 (TL Erwin, et al.)(NMNH: ADP007642, male), 3 October - 15 November 1983, NE Stork, et al.)(NMNH: ADP135873, female), 2 March 1984 (TL Erwin, et al.)(NMNH: ADP007643, male), 8 September 1984 (TL Erwin, et al.)(NMNH: ADP007641, female).**Venezuela**, Amazonas, Cerro de la Neblina, Rio Baria Basecamp, 0.837°N, 66.162°W, 138m, 10-20 February 1985 (PJ Spangler, PM Spangler, et al.)(NMNH: ADP006177, female), 20 February 1985 (PJ Spangler, PM Spangler, et al.)(NMNH: ADP006176, female), 21-28 February 1985 (PJ Spangler, PM Spangler, et al.)(NMNH: ADP005803, male).

###### Geographic distribution

(Fig. 11). This species is currently known from the type locality at Tefé, Brazil, and from Brazil – Amazonas, Pará, Rondonia; Ecuador, French Guiana, Perú, and Venezuela.

###### Notes.

Reichardt (1973) reported the following additional specimens that we did not see: **Brazil** – Amazonas: Itaituba (3 exs. MNHP); Manaus (1 ex. MZSP); Matuxadi, alto Rio Cauaburi (1 ex. MZSP): Tefé (4 exs. MNHP). Pará: Tapajos (2 exs. MNHP). Lorenz (1998, 2005) failed to record this species.

##### Hyboptera lucida

Taxon classificationAnimaliaColeoptera Carabidae

Henry & Erwin sp. n.

http://zoobank.org/BC3B2B01-C53D-48A6-B2B1-342CA9D2E53A

Pied humps-backed beetle Figs 7B , 8 , 10C , 11

###### Holotype.

Female. **French Guiana**, Cayenne, Commune de Roura, Montagne des Chevaux, 4.7127°N, 52.3966°W, 90m, 17 April 2011 (S Brule, PH Dalens, E Poirier)(NMNH: ADP128638).

###### Specific epithet.

The epithet ‘‘*lucida*’’ is a Latinized singular feminine adjective of lucid, for clear, referring to the translucent patch near the apex of the elytron on adult members of this species.

###### Proposed english vernacular name.

Pied humps-backed beetle.

###### Diagnosis.

With the attributes of the genus and *tuberculata* species group as described above and adults with only dark non-metallic markings on the pronotal disc; elytra black with 4 small pale medio-apical tubercles. Venter completely piceous. Largest adults in the genus.

###### Description.

(Figs 7B, 8, 10C). *Size*: See Appendix 1. Length (SBL) long for genus, ABL = 5.51–6.67 mm, SBL = 4.38–5.27 mm, TW = 2.36–3.25 mm.

*Color: See* diagnosis, above.

*Luster*: Without any metallic highlights, matte.

*Microsculpture*: Mostly isodiametric and stretched, well-impressed, cells somewhat more stretched around elytral tubercles.

*Head*: Rugae moderately coarse, mostly without patterned arrangement. Eye very large, hemispheric, evenly rounded. Antenna short, barely reaching humerus. Labrum rectangulate, shallowly bilobed, anterior margin slightly emarginate. Neck coarsely transversely rugose.

*Prothorax*: Pronotum markedly broad, disc centrally shallowly depressed with coarse angulate rugae. Lateral margins broadly explanate and evenly rounded to level of lateral seta then straight to obtuse hind angle, base medially produced and rounded posteriorly.

*Pterothorax*: Normal for Agrina, fully winged. Elytron intervals 3 with 10 and interval 5 with 7 prominent discal unisetiferous tubercles, interval 4 with sub-apical pale colored “lens,” other intervals moderately convex, side margin broadly explanate laterally only at middle third. Elytron broad and moderately short, much narrower in width than that of the pronotum at the broadest part, apex truncate, slightly rounded with distal corner broadly and obtusely rounded, disc not significantly convex, basal third slightly depressed. All interneurs well-impressed.

*Legs*: Femur dorso-ventrally moderately depressed, tibia coequal in length, more depressed; tarsus less than half the length of the tibia, fourth tarsomere markedly bilobed and with tarsal pad of setae.

*Abdomen*: Sparsely setiferous; normal ambulatory setae on sterna 3–5; female with two pairs of ambulatory setae on sternum 6, medial pair of setae less than the length of lateral pair; males with only the lateral pair of longer setae.

*Male genitalia*: Phallus (Fig. 10C) with ostium of 1/6 its length, catopic, apex very short, narrowly pointed, broadly rounded in dorsal aspect; endophallus with flagellum (obvious in illustration), flagellum not barbed. Parameres asymmetric, right very small, left larger.

*Female genitalia*: (Fig. 8). Ovipositor with broad triangular laterotergite (lt) and two robust gonocoxites (gc 1, gc 2); gonocoxite 1 apicolaterally not setose; gonocoxite 2 apically rounded, base (b) medium-size co-equal in width with blade (bl) which is short, blunt, with several dorsal ensiform setae (des), ventral ensiform seta absent, ensiform setae moderately short and robust; without ventral preapical nematiform setae. Reproductive tract proximally with moderately long, broad bursa copulatrix (bc), common oviduct (co) enters the bursa ventrally just anterior to bursal midlength, and long narrow corregated spermatheca (sp) distal to villous canal; spermathecal gland small, cylindrical; spermathecal gland duct (sgd) very narrow, attached to oviduct at base of its broadened portion.

###### Dispersal potential.

These beetles are macropterous and capable of flight. They are moderately swift and agile runners. Adults of this species are attracted to light traps, and have been collected in FIT, Malaise, and SLAM traps.

###### Way of life.

Adults are found in January-March, May-December, in all seasons, in lowlands (13–325 m.a.s.l.) in the lowland forests of Guyane.

###### Other specimens examined.

**French Guiana**, Cayenne, Commune de Roura, Montagne des Chevaux, 4.7127°N, 52.3966°W, 90m, 16 July 2011 (S Brule, PH Dalens, E Poirier)(NMNH: ADP135813, male paratype), 2 January 2016 (S Brule, PH Dalens, E Poirier)(NMNH: ADP148100, female paratype), 26 March 2016 (S Brule, PH Dalens, E Poirier)(NMNH: ADP148222, male paratype), 20 May 2015 (S Brule, PH Dalens, E Poirier)(NMNH: ADP148099, male paratype), 23 October 2011 (S Brule, PH Dalens, E Poirier)(NMNH: ADP135793, female paratype), 23 September 2011 (S Brule, PH Dalens, E Poirier)(NMNH: ADP135785, male paratype), 25 September 2011 (S Brule, PH Dalens, E Poirier)(NMNH: ADP135825, female paratype), 31 March 2013 (S Brule, PH Dalens, E Poirier)(NMNH: ADP135807, male paratype), 4 February 2013 (S Brule, PH Dalens, E Poirier)(NMNH: ADP135831, male paratype), 16 April 2016 (S Brule, PH Dalens, E Poirier)(NMNH: ADP152487, female paratype), Commune Matoury, La Desiree, 4.8449°N, 52.3484°W, 20m, 27 September 2014 (S Brule, PH Dalens, E Poirier)(NMNH: ADP148098, female paratype), Trou Poisson, 5.4206°N, 53.0716°W, 13m, 7 May 2014 (S Brule, PH Dalens, E Poirier)(NMNH: ADP135783, female paratype), 7 May 2014 (S Brule, PH Dalens, E Poirier)(NMNH: ADP135829, female paratype), Inselberg Nouragues, Commune de Regina, Saut Parare, 4.0334°N, 52.6786°W, 51m, 30 September 2009 (S Brule, PH Dalens, E Poirier)(NMNH: ADP126231, female paratype), Region de Saul, Commune de Saul, Belvedere de Saul (point de vue), 3.6223°N, 53.2159°W, 283-325m, 11 December 2012 (S Brule, PH Dalens, E Poirier)(NMNH: ADP134135, female paratype), 20 December 2010 (S Brule, PH Dalens, E Poirier)(NMNH: ADP130776, female paratype), 21 June 2011 (S Brule, PH Dalens, E Poirier)(NMNH: ADP134134, female paratype), Cirque Orfion, Orapu RN2 PK65, Commune de Regina, 4.4962°N, 52.3454°W, 81m, 17 September 2016 (S Brule, PH Dalens, E Poirier)(NMNH: ADP151235, male paratype).

###### Geographic distribution

(Fig. 11). This species is currently known from the type locality in French Guiana and nearby areas.

##### Hyboptera tuberculata

Taxon classificationAnimaliaColeoptera Carabidae

(Dejean), 1825

Tuberculate humps-backed beetle Figs 9A , 10D , 11

Lebia tuberculata Dejean, 1825: 272.
Cryptobatis tuberculata ; Gemminger and Harold (1868: 135).
Hyboptera tuberculata (Dejean); Chaudoir (1872: 162).
Aspasia verrucosa Reiche; Reichardt (1973: 53).

###### Lectotype.

Here designated. Male. **French Guiana**, Cayenne, (MNHP).

###### Derivation of specific epithet.

The species epithet ‘‘*tuberculata*’’ is a Latinized singular feminine adjective, referring to the bumpy attributes of the elytra.

###### Proposed English vernacular name.

Tuberculate humps-backed beetle.

###### Diagnosis.

With the attributes of the genus and *tuberculata* species group as described above and adults with only dark non-metallic markings on the pronotal disc, elytron just posterior to scutellum with only the suture pale in color, otherwise markedly infuscate.

###### Description.

(Figs 9A, 10D). *Size*: See Appendix 1. Length (SBL) long for genus, ABL = 4.32–5.88 mm, SBL = 3.94–4.85 mm, TW = 2.18–3.12 mm.

*Color: See* diagnosis, above.

*Luster*: Without any metallic highlights, shiny.

*Microsculpture*: Mostly isodiametric and stretched, shallowly impressed, cells somewhat more stretched around elytral tubercles.

*Head*: Rugae moderately coarse, mostly chaotic. Eye markedly large, sub-hemispheric, evenly rounded anteriorly, subtly more prolonged posteriorly. Antenna short, barely reaching humerus. Labrum rectangulate, shallowly bilobed, anterior margin slightly emarginate. Neck transversely finely rugose.

*Prothorax*: Pronotum markedly broad, disc centrally moderately depressed with coarse angulate rugae. Lateral margins broadly explanate and evenly rounded then straight to obtuse hind angle, base medially produced and rounded posteriorly.

*Pterothorax*: Normal for Agrina, fully winged. Elytron intervals 3 with 8(9) and interval 5 with 6(7) prominent discal unisetiferous tubercles, interval 4 and 6 with subapical pale colored “lens,” other intervals moderately convex, side margin broadly explanate laterally only at middle third. Elytron broad and moderately short, moderately narrower in width than that of the pronotum at the broadest part, apex truncate, slightly rounded with distal corner broadly and obtusely rounded, disc not significantly convex, basal third slightly depressed. All interneurs well-impressed.

*Legs*: Femur dorso-ventrally moderately depressed, tibia coequal in length, more depressed; tarsus less than half the length of the tibia, fourth tarsomere markedly bilobed and with tarsal pad of setae.

*Abdomen*: Sparsely setiferous; normal ambulatory setae on sterna 3–5; female with two pairs of ambulatory setae on sternum 6, medial pair of setae less than the length of lateral pair; males with only the outer pair of longer setae.

*Male genitalia*: Phallus (Fig. 10D) robust with ostium of 1/6 its length, catopic, apex very short, broadly rounded in dorsal and lateral aspects; endophallus with flagellum (obvious in illustration), flagellum not barbed. Parameres asymmetric, right very small, left larger.

*Female genitalia*: Not investigated, likely similar to that of *H. lucida* (Fig. 8).

###### Dispersal potential.

These beetles are macropterous and capable of flight. They are moderately swift and agile runners. Adults of this species are attracted to C.D.C. Light traps.

###### Way of life.

Adults are common in the lowlands and lower midlands (7–914 m a.s.l.) and appear to be generalists in a variety of rainforest biotopes including terra firme, dry and humid tropical forests, in gallery forests, and savanna forest patches. In these forests, they are commonly found in big trees with vines and epiphytes, in suspended medium to large-sized dry leaves, and in dense vine tangle plus leaves with light bamboo occurrence. Individuals can be found from January to December, in both the rainy and dry seasons. Member of this species have been recorded from the canopy of the following tree species using insecticidal fogging techniques: Pouteria reticulata ssp. reticulata cf.; *Sloanea cordia*; *Browneopsis ucayalina*; *Talisia* bitter; *Mouriri guapira*; *Trichilia solitudinis*; *Eriotheca globosa* cf.; *Sloanea* 1; *Trichilia solitudinis*; *Simira cordifolia/rubescens* cf.; *Sarcaulus brasiliensis* aff. “burnt”; *Catatola costaricana* aff.; Pourouma mollis ssp. triloba cf.; *Alchornea triplinervia* cf.; *Duguetia surinamensis* cf.; *Casearia avitensis* cf.; *Nectandra crassiloba* cf.; *Trichilia rubra* cf.; *Coccoloba densifrons* cf. traditional; *Iriartea deltoidea*; *Micropholis venulosa* cf.; *Sagotia racemosa*; *Virola obovata*; *Pouteria baehniana* cf.; Pourouma bicolor ssp. bicolor cf.; *Inga bourgonii* cf.; *Garcinia macrophylla* cf.; *Chrysobalanaceae surreptitious*; *Oenocarpus bataua*; *Neea* dive-tuberculate; *Semaphyllanthe megistocaula* cf.; Lauraceae redvein.

###### Other specimens examined.

**Bolivia**, Beni, 53 km E San Borja, Estación Biológica Beni, Palm Camp, 14.8676°S, 66.3265°W, 177m, 25-30 July 1988 (TL Erwin)(NMNH: ADP005759, male, ADP135777, female). **Brazil**, Amazonas, north of Manaus, on Amazonas 010 at Km 26, Reserva Ducke, 2.918°S, 59.971°W, 70m, 27 October 1977 (J Arias)(NMNH: ADP005758, female), 8 August 1978 (J Arias)(NMNH: ADP135819, female); Sergipe, Estação Ecológica da Serra de Itabaiana, 10.7297°S, 37.3064°W, 166m, 14-20 September 1999 (A Bonaldo)(NMNH: MCM166.415, ADP144921, male). **Colombia**, Amazonas, PNN Amacayacu, Mocagua, 3.84°S, 70.22°W, 76m, 3-9 April 2000 (A Parente)(IAvH: IAvH-E-3063, ADP145177, male)(IAvH: IAvH-E-10944, ADP145183, female), 6-12 June 2000 (A Parente)(IAvH: IAvH-E-73787, ADP145145, male), 12-19 June 2000 (A Parente)(IAvH: IAvH-E-3056, ADP145193)(IAvH: IAvH-E-3093, ADP145203)(IAvH: IAvH-E-3095, ADP145173, males)(IAvH: IAvH-E-3096, ADP145187, female), 26 June - 6 July 2000 (A Parente)(IAvH: IAvH-E-73786, ADP145175, male), 7-19 July 2000 (A Parente)(IAvH: IAvH-E-40755, ADP145171)(IAvH: IAvH-E-73785, ADP145169, males)(IAvH: IAvH-E-73775, ADP145181, female); Bolivar, Zambrano, Hda. Monterrey - Rio Magdalena, 9.6293°N, 74.9123°W, 70m, 16-30 August 1993 (F Fernanadez, G Ulloa)(IAvH: IAvH-E-2449, ADP145154, female), September 1993 (F Fernanadez, G Ulloa)(IAvH: IAvH-E-73780, ADP145186, male), 8 October 1993 (G Ulloa, F Fernanadez)(IAvH: IAvH-E-2449, ADP145182, female), SFF Los Colorados, Diana, 9.90°N, 75.11°W, 237m, 2-16 January 2001 (E Deulufeut)(IAvH: IAvH-E-2320, ADP145194)(IAvH: IAvH-E-73779, ADP145180, males)(IAvH: IAvH-E-73778, ADP145196, female), La Suiris, 9.900°N, 75.1154°W, 236m, 1-15 September 2001 (E Deulufeut)(IAvH: IAvH-E-40756, ADP145184, male), SFF Los Colorados Venado, 9.900°N, 75.1153°W, 235m, 2-17 October 2000 (E Deulufeut)(IAvH: IAvH-E-73776, ADP145190)(IAvH: IAvH-E-73777, ADP145188, females); Casanare, Aguazul, 5.18°N, 72.55°W, 701m, 22 September 1995 (F Fernanadez)(IAvH: IAvH-E-2450, ADP145152, female); Magdelena, PNN Tayrona, Canaveral, 11.342°N, 074.031°W, 25m, 17 October - 3 November 2000 (R Henriquez)(IAvH: IAvH-E-73781, ADP145178, male)(IAvH: IAvH-E-73782, ADP145174)(IAvH: IAvH-E-73783, ADP145170, females), 4-15 December 2000, R Henriquez)(IAvH: IAvH-E-3532, ADP145176)(IAvH: IAvH-E-73784, ADP145166, females), Vichada, PNN Tuparro, 5.36°N, 67.84°W, 71m, 8-14 December 2000 (W Villalba)(IAvH: IAvH-E-2741, ADP145148, male). **Ecuador**, Orellana, Reserva Ethnica Huaorani, 39 km S Pompeya, Estación Científica Yasuní - Onkone Gare Camp, Erwin Piraña Plot, transect 10, station 2, 0.6590°S, 76.453°W, 220-250m, 8 October 1995 (TL Erwin, et al.)(NMNH: ADP137752, female paratype), Erwin Piraña Plot, transect 10, station 5, 0.6540°S, 76.453°W, 220-250m, 4 October 1996 (TL Erwin, et al.)(NMNH: ADP137369, male paratype), Erwin Piraña Plot, transect 10, station 9, 0.6540°S, 76.453°W, 220-250m, 23 January 2006 (TL Erwin, et al.)(NMNH: ADP133868, male paratype), Erwin Piraña Plot, transect 4, station 4, 0.6570°S, 76.498°W, 220-250m, 3 July 1995 (TL Erwin, et al.)(NMNH: ADP135805, male paratype), Erwin Piraña Plot, transect 5, station 2, 0.6566°S, 76.490°W, 220-250m, 12 February 1995 (TL Erwin, et al.)(NMNH: ADP005762, female paratype), Erwin Piraña Plot, transect 5, station 2, 0.6566°S, 76.496°W, 220-250m, 9 October 1994 (TL Erwin, et al.)(NMNH: ADP138806, male paratype), Erwin Piraña Plot, transect 6, station 1, 0.6561°S, 76.483°W, 220-250m, 7 February 1996 (TL Erwin, et al.)(NMNH: ADP137383, male paratype), Erwin Piraña Plot, transect 6, station 1, 0.6561°S, 76.485°W, 220-250m, 29 January 2006 (TL Erwin, et al.)(NMNH: ADP133852, female paratype), Erwin Piraña Plot, transect 6, station 4, 0.6561°S, 76.4483°W, 220-250m, 22 June 1996 (TL Erwin, et al.)(NMNH: ADP137355, female paratype), Erwin Piraña Plot, transect 6, station 6, 0.6561°S, 76.483°W, 220-250m, 22 July 1996 (TL Erwin, et al.)(NMNH: ADP137746, male paratype), Erwin Piraña Plot, transect 6, station 7, 0.6561°S, 76.4483°W, 220-250m, 2 October 1996 (TL Erwin, et al.)(NMNH: ADP135891, male paratype), Erwin Piraña Plot, transect 6, station 8, 0.6561°S, 76.4483°W, 220-250m, 7 February 1996 (TL Erwin, et al.)(NMNH: ADP137353, female paratype), Erwin Piraña Plot, transect 8, station 4, 0.6551°S, 76.4403°W, 220-250m, 7 October 1995 (TL Erwin, et al.)(NMNH: ADP135970, male paratype), Erwin Piraña Plot, transect 8, station 6, 0.6551°S, 76.4483°W, 220-250m, 7 October 1995 (TL Erwin, et al.)(NMNH: ADP137760, female paratype); Yasuni National Park (edge), 95.43 km E (heading 101.46°) Coca, Tiputini Biodiversity Station, Erwin Harpia Plot: transect 4, station 8, 0.6316°S, 76.1443°W, 208m, 24 October 1998 (TL Erwin, et al.)(NMNH: ADP135821, male), Erwin Harpia Plot: transect 8, station 4, 0.6278°S, 76.1443°W, 203m, 4 July 1998 (TL Erwin, et al.)(NMNH: ADP135797, male). Yasuni National Park (edge), 95.43 km E (heading 101.46°) Coca, Tiputini Biodiversity Station, Erwin Harpia Plot, transect 10, station 6, 0.6262°S, 76.1443°W, 214m, 21 October 1998 (TL Erwin, et al.)(NMNH: ADP135775, female paratype), Erwin Harpia Plot, transect 10, station 9, 0.6269°S, 76.1443°W, 214m, 5 July 1998 (TL Erwin, et al.)(NMNH: ADP135803, female paratype), Erwin Harpia Plot, transect 3, station 10, 0.6332°S, 76.1443°W, 207m, 8 February 1999 (TL Erwin, et al.)(NMNH: ADP135773, male paratype), Erwin Harpia Plot, transect 4, station 1, 0.6316°S, 76.1443°W, 208m, 24 October 1998 (TL Erwin, et al.)(NMNH: ADP135779, female paratype), Erwin Harpia Plot, transect 6, station 9, 0.6295°S, 76.1443°W, 199m, 7 February 1999 (TL Erwin, et al.)(NMNH: ADP135835, female paratype), Erwin Harpia Plot, transect 7, station 1, 0.6295°S, 76.1443°W, 203m, Erwin Harpia Plot: transect 4, station 4, 0.6316°S, 76.1443°W, 208m, 20 February 2001 (TL Erwin, et al.)(NMNH: ADP139100, female), 29 September 2000 (TL Erwin, et al.)(NMNH: ADP139132, female paratype), Erwin Harpia Plot, transect 7, station 6, 0.6287°S, 76.1443°W, 203m, 6 February 1999 (TL Erwin, et al.)(NMNH: ADP135827, male paratype); Sucumbíos, Río Napo, Sacha Lodge, Pilchicocha, 0.472°S, 76.459°W, 228m, 12-22 February 1994 (P Hibbs)(SEMC: ADP005757, female paratype), 24 May - 3 June 1994 (P Hibbs)(SEMC: ADP005756, female paratype). **French Guiana**, Cayenne, Commune de Roura, Montagne des Chevaux, 4.7127°N, 52.3966°W, 90m, 23 January 2016 (S Brule, PH Dalens, E Poirier)(NMNH: ADP144920, male). **Guyana**, Bartica, 6.3970°N, 58.6268°W, 20m, 12 May 1924 (AMNH: ADP005760, male). **Panamá**, Chiriqui, Volcan de Chiriqui, 610-914m, 26 May 1983 (GC Champion)(BMNH: ADP144995, male); Cocle, El Valle, 8.603°N, 80.152°W, 800m, 25-28 May 1983 (WE Steiner)(NMNH: ADP144994, male). **Perú**, Loreto, Pacaya-Samiria National Reserve, Río Samiria, Cocha Shinguito, 5.1775°S, 76.6556°W, 112m, 22 May 1990 (TL Erwin, et al.)(NMNH: ADP086940, male, ADP086960, female); Madre de Dios, Manu Reserved Zone, Río Manu, BIOLAT Biological Station, 11.9446°S, 71.2831°W, 356m, August-September 1988 (TL Erwin)(NMNH: ADP007532, ADP007535, males, ADP007533, ADP007534, females), 7 September 1988 (TL Erwin)(NMNH: ADP007550, female), 21 September 1991 (TL Erwin)(NMNH: ADP007552, male), 30 September 1991 (TL Erwin, MG Pogue)(NMNH: ADP135823, male), 22 June 1993 (TL Erwin, F. Pfuno S)(NMNH: ADP007553, ADP007554, femaemales), Reserva Nacional Tambopata, 30 km (air) SW Puerto Maldonado, Explorer’s Inn, 12.8364°S, 69.2936°W, 209m, 30 April 1984 (TL Erwin, et al.)(NMNH: ADP005754, male), 2 May 1984 (TL Erwin, et al.)(NMNH: ADP005755, female). **Suriname**, Paramaribo, Combe, 5.8427°N, 55.1600°W, 15m, 6 January 1956 (DC Geijskes)(NBCL: ADP005761, male); Wanica, Lelydorp, Sumatra weg, 5.700°N, 55.198°W, 7m, 28 November 1939 (DC Geijskes)(NBCL: ADP005805, male).

###### Geographic distribution

(Fig. 11). This species is currently known from the type locality in French Guiana and nearby areas and from Bolivia, Brazil – (Amazonas, Sergipe), Colombia, Ecuador, Guyana, Perú, and Suriname.

###### Notes.

Reichardt (1971) synonomized the following species with *H. tuberculata* Dejean. We do not agree with this based on the numerous attributes we studied and included in the descriptions.

##### Hyboptera verrucosa

Taxon classificationAnimaliaColeoptera Carabidae

(Reiche), 1842, stat. rest.

Verrucose humps-backed beetle Figs 9B , 10E , 11

Aspasia verrucosa Reiche, 1842: 311.
Hyboptera verrucosa (Reiche); Chaudoir (1872: 164).
Lebia tuberculata Dejean; Reichardt (1971: 53).

###### Holotype. Sex unknown.

**Colombia** (“Nouvelle-Grenade”), (MNHP). Type locality herewith restricted to **Colombia**, Amazonas, Leticia, 4.2242°S, 69.9449°W, 70 m.s.a.l.

###### Derivation of specific epithet.

The specific epithet, *verrucosa*, is a feminine Latin adjective referring to the tuberculate elytra.

###### Proposed English vernacular name.

Verrucose humps-backed beetle.

###### Diagnosis.

With the attributes of the genus and *tuberculata* species group as described above and adults with only dark non-metallic markings on the pronotal disc, pronoum with rounded margins, elytron just posterior to scutellum with a V-shaped pale area encompassing the scutellum and sutural and first interval, venter substantially infuscate, and elytra narrow, not broadly arcuate.

###### Description.

(Figs 9B, 10E). *Size*: See Appendix 1. Length (SBL) long for genus, ABL = 4.47–5.66 mm, SBL = 3.79–4.90 mm, TW = 2.06–2.85 mm.

*Color: See* diagnosis, above.

*Luster*: Without any metallic highlights, shiny and matte.

*Microsculpture*: Mostly isodiametric and stretched, shallowly impressed, cells somewhat more stretched around elytral tubercles.

*Head*: Rugae moderately coarse, mostly without patterned arrangement. Eye markedly large, sub-hemispheric, evenly rounded anteriorly, subtly more prolonged posteriorly. Antenna short, barely reaching humerus. Labrum rectangulate, shallowly bilobed, anterior margin slightly emarginate. Neck finely transversely rugose.

*Prothorax*: Pronotum moderately broad, disc centrally depressed with coarse angulate rugae. Lateral margins broadly explanate and evenly rounded to obtuse hind angle, base medially produced and rounded posteriorly.

*Pterothorax*: Normal for Agrina, fully winged. Elytron intervals 3 with 8, and interval 5 with 6 prominent discal unisetiferous tubercles, interval 4 with sub-apical pale colored “lens,” other intervals moderately convex, side margin broadly explanate laterally only at middle third. Elytron broad and moderately short, much narrower in width than that of the pronotum at the broadest part, apex truncate, slightly rounded with distal corner broadly and obtusely rounded, disc not significantly convex, basal third slightly depressed. All interneurs well-impressed.

*Legs*: Femur dorso-ventrally moderately depressed, tibia coequal in length, more depressed; tarsus less than half the length of the tibia, fourth tarsomere markedly bilobed and with tarsal pad of setae.

*Abdomen*: Sparsely setiferous; normal ambulatory setae on sterna 3–5; female with two pairs of ambulatory setae on sternum 6, medial pair of setae less than the length of lateral pair; males with only the lateral pair of longer setae.

*Male genitalia*: (Fig. 10E). With ostium of 1/5 its length, catopic, apex short, rounded; endophallus with flagellum, flagellum not barbed. Parameres asymmetric, right very small, left larger.

*Female genitalia*: Not investigated, likely similar to that of *H. lucida* (Fig. 8).

###### Dispersal potential.

These beetles are macropterous and capable of flight. They are moderately swift and agile runners. Adults of this species are attracted to C.D.C. light traps.

###### Way of life.

Adults are common in the lowlands (6 to 829 m.a.s.l.) and appear to be generalists in rainforest biotopes such as terra firme and secondary rain/pine forest. In these forests, they are commonly found in big trees with vines and epiphytes, in suspended dry *Cecropia* leaves, and in dry *Astrocaryum chambira* Burret palm frond skirts. Individuals can be found in January-November, in both the rainy and dry seasons. Member of this species have been recorded from the canopy of the following tree species using insecticidal fogging techniques: *Chrysophyllum argenteum* cf.; *Sterculia colombiana* cf.; *Parkia multijuga* cf.; *Naucleopsis herrerensis* cf.; *Matisia malacocalyx* cf.; *Pseudolmedia laevigata*; *Browneopsis ucayalina*; *Simira cordifolia/rubescens* cf.; *Brownea grandiceps* cf.; *Talisia* bitter; *Virola decorticans* cf.; Pouteria cuspidata ssp. robusta cf.; *Diospyros sericea*; *Guarea silvatica*; *Lacistema nena* cf.; *Guatteria glaberrima* cf.; *Luehea seemanni*; *Cordia alliodora*.

###### Other specimens examined.

**Brazil**, Amazonas, km 60 N Manaus, 18.1 km W Campinas Field Station, 2.468°N, 60.156°W, 113m, 22 February 1979 (TL Erwin, et al.)(NMNH: ADP007595, male), north of Manaus, on Amazonas 010 at Km 26, Reserva Ducke, 2.918°S, 59.971°W, 70m, 29 November 1977 (J Arias)(NMNH: ADP007619, male). **Colombia**, Amazonas, Leticia, 4.2242°S, 69.9449°W, 70m, 19-25 February 1972 (HF Howden, A Howden)(NMNH: ADP007579, male). **Ecuador**, Orellana, Reserva Ethnica Huaorani, 39 km S, Pompeya, Estación Científica Yasuní - Onkone Gare Camp, Erwin Piraña Plot: transect 2, station 6, 0.6581°S, 76.4513°W, 220-250m, 22 January 1994 (TL Erwin, et al.)(NMNH: ADP138694, male), Erwin Piraña Plot: transect 10, station 9, 0.6540°S, 76.4453°W, 220-250m, 23 January 1994 (TL Erwin, et al.)(NMNH: ADP138588, male), Erwin Piraña Plot: transect 8, station 3, 0.6551°S, 76.4403°W, 220-250m, 24 January 1994 (TL Erwin, et al.)(NMNH: ADP138834, male), Erwin Piraña Plot: transect 7, station 10, 0.6556°S, 76.4474°W, 220-250m, 7 October 1994 (TL Erwin, et al.)(NMNH: ADP135867, female), Erwin Piraña Plot: transect 6, station 9, 0.6561°S, 76.4483°W, 220-250m, 12 February 1995 (TL Erwin, et al.)(NMNH: ADP135871, male), Erwin Piraña Plot: transect 1, station 4, 0.6586°S, 76.4521°W, 220-250m, 4 October 1995 (TL Erwin, et al.)(NMNH: ADP135911, male), Erwin Piraña Plot: transect 6, station 10, 0.6561°S, 76.4483°W, 220-250m, 2 October 1996 (TL Erwin, et al.)(NMNH: ADP135924, female), Yasuni National Park (edge), 95.43 km E (heading 101.46°), Coca, Tiputini Biodiversity Station, Erwin Harpia Plot: transect 6, station 1, 0.6295°S, 76.1443°W, 220-250m, 26 October 1998 (TL Erwin, et al.)(NMNH: ADP135869, female). **French Guiana**, Cayenne, Commune de Roura, Montagne des Chevaux, 4.7127°N, 52.3966°W, 90m, 19 October 2013 (S Brule, PH Dalens, E Poirier)(NMNH: ADP140530, male), 29 March 2014 (S Brule, PH Dalens, E Poirier)(NMNH: ADP140518, female), Region de Saul, Commune de Saul, Belvedere de Saul, 3.6223°N, 53.2159°W, 283-325m, 23 February 2011 (S Brule, PH Dalens, E Poirier)(NMNH: ADP134156, female), 17 October 2012 (S Brule, PH Dalens, E Poirier)(NMNH: ADP134297, male), Foret de Maya, Commune Macouria, 4.9552°N, 52.4603°W, 30m, 19 December 2016 (S Brule, PH Dalens, E Poirier)(NMNH: ADP151234, female), Cirque Orfion, Orapu RN2 PK65, Commune de Regina, 4.4962°N, 52.3454°W, 81m, 24 September 2016 (S Brule, PH Dalens, E Poirier)(NMNH: ADP151233, male). **Panamá**, Canal Zone, Ancón, 8.9588°N, 79.5541°W, 36m, 5 November 1921 (H Osborn)(NMNH: ADP058099, female), Lion Hill Island, 9.2266°N, 79.8916°W, 68m, 29 June 1981 (RB Kimsey)(UCD: ADP056612, female), Paraiso, 9.033°N, 79.628°W, 106m, 11 January 1911 (EA Schwarz)(NMNH: ADP007952, female), 24 April 1911 (EA Schwarz)(NMNH: ADP007950, male), 2.2 km W Frijoles, 9.1736°N, 79.7966°W, 37m, 25 July 1977 (RB Kimsey, LS Kimsey)(UCD: ADP058775, female), 4.8 km W Cocoli, 8.980°N, 79.629°W, 188m, 30 August 1974, HP Stockwell, 046080, female), 8 km NW Gamboa, 9.1639°N, 79.7492°W, 100m, 15 July 1976 (TL Erwin, et al.)(NMNH: ADP055841, male), Barro Colorado Nature Monument, Barro Colorado Island, Barro Colorado Research Station, 9.1652°N, 79.8368°W, 70m, 26 June 1926 (NMNH: ADP 007951, female), May 1929 (PJ Darlington Jr.)(MCZ: ADP007948, male, 007957, female), 2 July 1938 (EC Williams)(CMNH: ADP007956, male), January 1941 (KW Cooper)(MCZ: ADP007949, ADP007955, females), April - May 1942 (NMNH: ADP007954, female), 19 February 1975 (TL Erwin, JL Lawrence)(NMNH: ADP027571, female), March 1975 (TL Erwin, JL Lawrence)(NMNH: ADP042850, male), 10 May 1977 (H Wolda)(NMNH: ADP079070, male), 2 June 1977 (H Wolda)(NMNH: ADP077804, female), 5 June 1977 (H Wolda)(NMNH: ADP078274, female), 25 September 1977 (H Wolda)(NMNH: ADP076934, male), 1 November 1977 (H Wolda)(NMNH: ADP082523, male), 24 April 1978 (H Wolda)(NMNH: ADP089657, male), 24 April 1978 (H Wolda)(NMNH: ADP089660, female), 19 May 1978 (H Wolda)(NMNH: ADP062493, male), 22 May 1978 (H Wolda)(NMNH: ADP066554, male), 13 June 1978 (H Wolda)(NMNH: ADP065729, female), 4 October 1978 (H Wolda)(NMNH: ADP080371, female), Parque Nacional Soberania, Madden Forest, 9.098°N, 79.616°W, 50m, 3 July 1974 (C O’Brien, L O’Brien, B Marshall)(NMNH: ADP027330, male); Cocle, El Valle, 8.603°N, 80.152°W, 800m, 16 May 1973 (HP Stockwell)(NMNH: ADP047483, female), El Valle, 8.603°N, 80.152°W, 829m, 26 May 1983 (WE Steiner)(NMNH: ADP005802, female); Panamá, Parque Natural Metropolitano, Panamá City, 8.9946º N, 79.5428º W, 67m, 13 May 1996 (F. Øedegaard)(NMNH: ADP140497, male), 27 September 1995, (F. Øedegaard)(NMNH: ADP140507, male), Portobelo, 9.555°N, 69.653°W, 10m, 11 March 1911 (EA Schwarz)(NMNH: ADP007953, female). **Perú**, Loreto, Pacaya-Samiria National Reserve, Río Samiria, Cocha Shinguito, 5.1775°S, 76.6556°W, 112m, 19 June 1990 (TL Erwin, et al.)(NMNH: ADP094065, female), 20 August 1991 (TL Erwin, MG Pogue)(NMNH: ADP051383, female), 22 May 1990 (TL Erwin, et al.)(NMNH: ADP086946, ADP086980, ADP086980, females); Madre de Dios, Reserva Nacional Tambopata, 30 km (air) SW, Puerto Maldonado, Explorer’s Inn, 12.8364°S, 69.2936°W, 209m, 25 February 1984 (TL Erwin, et al.)(NMNH: ADP007594, female), Manu Reserved Zone, Río Manu, BIOLAT Biological Station, Pakitza, 11.9446°S, 71.2831°W, 356m, 9 September 1988 (TL Erwin)(NMNH: ADP007556, male), 14 October 1991 (TL Erwin, MG Pogue)(NMNH: ADP007555, male), 10 July 1992 (TL Erwin, GP Servat, D Silva, F Pfuno S, E Pfuno S)(NMNH: ADP007572, female), 22 June 1993 (TL Erwin, F Pfuno S)(NMNH: ADP007557, female). **Suriname**, Kwatta, Warwabos, weg nr zee, 5.8437°N, 55.1586°W, 6m, 28 January - 1 February 1964 (DC Geijskes)(NBCL: ADP005808, male, ADP005806, female); Paramaribo, Ma Retraite, 5.8437°N, 55.1586°W, 6m, 12-14 February 1964 (DC Geijskes)(NBCL: ADP005807, male), Wanica, Lelydorp, Sumatra weg, 5.700°N, 55.198°W, 7m, 26-31 March 1964 (DC Geijskes)(NBCL: ADP007598, female). **Trinidad and Tobago**, Tunapuna, Mt. St. Benedict Monastery, nr. PAX House, 10.6624°N, 61.3984°W, 204m, 8-9 July 1999 (GB Edwards)(NMNH: ADP112207, male, ADP112233, female). **Venezuela**, Bolivar, 22 km E Upata, 7.960°N, 62.212°W, 359m, 18-19 June 1996 (HF Howden, A Howden)(NMNH: ADP007596, female).

###### Geographic distribution

(Fig. 11). This species is currently known from the restricted type locality (see above) in Colombia, and from Brazil – Amazonas; Ecuador, French Guiana, Panamá, Perú, Suriname, Trinidad and Tobago, and Venezuela.

###### Notes.

Reichardt (1973) reported additional localities under his treatment for this name; however, he also synonymized *H. verrucosa* with *H. tuberculata* in Reichardt (1971). Since we do not accept his action and regard them both as good species, we cannot use his locality records without access to the specimens he saw in order for us to correct his identifications.

## Summary and future directions

Most of the 738 specimens used in this study were taken from the rainforest canopy, or upper understory, using insecticidal fogging techniques. One *H. auxilidora* adult was found by George Vogt in Texas under the webbing of a live colony of Psocoptera, reminiscent of adults of *Hyboteroides* Erwin & Ball species that live in the colonies of Embioptera (Embiidina) under their webbing (Erwin and Ball 2012). These rather flattened ‘‘blattiform’’ beetles with dorso-ventrally flattened legs and depressed bodies may live normally under the silken nets of their hosts on tree trunks and branches in the canopy and understory. If so, they may prey on the hosts with their long stiletto-like galea apices and lacinial teeth and numerous long setae on the mouthparts (reminding one of the Collembola-seizing adults of *Loricera* and *Leistus*). Such mouthparts may aid in capturing soft-bodied psocids and embiids. Adults of *Hyboptera* are also known from large bombacaceous anther rings on the forest floor in the dry season (Barro Colorado Island, Panamá – *Pseudobombax septenatum* (Jacq.) Dugand) and from FITs, malaise traps, SLAM traps, and UV/White lights. Amongst the lineages of Cryptobatida, both *Hybopteroides* and *Thoasia* adults share several structural attributes, in addition to the mouthparts mentioned above, with *Hyboptera* adults, such as a serial row of long setae on three or more elytral intervals, angulate (or, subangulate) lateral margin of the pronotum, short antennae, broadly depressed mandibles, etc.

With regard to the genus *Hyboptera*, the recent discoveries of several new species in remote parts of the upper Amazon Basin suggests that further sampling in such areas will increase the species richness of this markedly (structurally and behaviorally) interesting (architecturally and behaviorally) lineage of Carabidae. We also note that even though at present there are not many species known and adults are morphologically markedly modified from more “typical” carabids, and have a unique way of life preying on insects under webbing, the lineage is widely dispersed from Texas to southeastern Brazil with many species that are widespread in their distributions.

Adults of the (currently) monobasic *Thoasia* Liebke, 1939 are exceedingly common in canopy fogging samples (Erwin 1991); however, nothing is known about their way of life and they are only known with precise location from foggings in Perú and Ecuador and FIT samples in French Guiana. Feeding specializations such as those hypothesized herein for adult *Hyboptera* and *Hybopteroides* and commonality of morphological attributes offer a fertile field of study on *Thoasia* for coleopterists eager to spend long periods of time in the rainforest canopies. However, before that, *Thoasia* is in need of a taxonomic revision and three undescribed species need to be treated (cf. Erwin et al. 2012; Erwin, in prep). Liebke’s holotype of the type species, *Thoasia rugifrons*, is in the Polish Academy of Sciences Collection according to Mroczkowski (1960).

## Supplementary Material

XML Treatment for Hyboptera

XML Treatment for Hyboptera angulicollis

XML Treatment for Hyboptera biolat

XML Treatment for Hyboptera vestiverdis

XML Treatment for Hyboptera scheelea

XML Treatment for Hyboptera shasta

XML Treatment for Hyboptera tepui

XML Treatment for Hyboptera tiputini

XML Treatment for Hyboptera viridivittis

XML Treatment for Hyboptera apollonia

XML Treatment for Hyboptera auxilidora

XML Treatment for Hyboptera dilutior

XML Treatment for Hyboptera lucida

XML Treatment for Hyboptera tuberculata

XML Treatment for Hyboptera verrucosa

## Appendix 1

Morphological measurements and ratios for adults of species of *Hyboptera* Chaudoir 1872. All values are in millimeters. Apparent body length (ABL) is also provided in the descriptions. Means provided for ratios are “harmonic means.”

**Table:**

***angulicollis* specigroup**				
**A.** *Hyboptera angulicollis* Chaudoir				
	**Males (N19)**		**Females (11)**	
	**Range**	**Mean**	**Range**	**Mean**
Total Length (SBL)	3.344–6.958	3.695	3.353–4.148	3.724
Maximum Width	1.944–2.284	2.135	1.924–2.532	2.204
Width of Head / Width of Left Elytron	0.836–0.977	0.914	0.814–0.963	0.891
Pronotum: Width (at widest part) / Length	1.647–1.978	1.81	1.718–1.909	1.802
Length of Pronotum / Length of Head	1.256–1.495	1.364	1.279–1.515	1.381
ABL	3.943–4.800	4.296	4.015–4.795	4.377
**B.** *Hyboptera apollonia* Erwin				
	**Males (N2)**		**Females (N6)**	
	**Range**	**Mean**	**Range**	**Mean**
Total Length (SBL)	3.723–4.263	3.975	3.534–4.123	3.835
Maximum Width	2.168–2.502	2.327	2.098–2.574	2.3
Width of Head / Width of Left Elytron	0.889–0.927	0.908	0.810–0.914	0.888
Pronotum: Width (at widest part) / Length	1.703–1.714	1.709	1.799–1.955	1.861
Length of Pronotum / Length of Head	1.230–1.353	1.288	1.211–1.407	1.32
ABL	4.534–5.144	4.82	4.199–5.067	4.566
**C.** *Hyboptera biolat* Erwin & Henry, sp. n.				
	**Males (N9)**		**Females (N2)**	
	**Range**	**Mean**	**Range**	**Mean**
Total Length (SBL)	3.159–3.744	3.458	3.207–3.482	3.339
Maximum Width	1.728–2.328	1.981	1.676–1.996	1.822
Width of Head / Width of Left Elytron	0.832–0.996	0.937	0.964–1.058	1.009
Pronotum: Width (at widest part) / Length	1.738–1.965	1.85	1.793–1.943	1.865
Length of Pronotum / Length of Head	1.300–1.429	1.345	1.396–1.518	1.455
ABL	3.714–4.166	3.969	3.638–3.855	3.743
**D.** *Hyboptera vestiverdis* Henry & Erwin, sp. n.				
	**Males (N15)**		**Females (N15)**	
	**Range**	**Mean**	**Range**	**Mean**
Total Length (SBL)	3.339–3.992	3.611	3.135–3.956	3.65
Maximum Width	1.896–2.548	2.115	1.826–2.428	2.127
Width of Head / Width of Left Elytron	0.834–1.016	0.93	0.845–0.983	0.924
Pronotum: Width (at widest part) / Length	1.698–2.003	1.861	1.680–1.993	1.82
Length of Pronotum / Length of Head	1.225–1.533	1.354	1.284–1.519	1.389
ABL	3.958–4.623	4.272	3.461–4.550	4.201
**E.** *Hyboptera scheelea* Erwin & Henry, sp. n.				
	**Males (N0)**		**Females (N1)**	
	**Range**	**Mean**	**Range**	**Mean**
Total Length (SBL)				3.683
Maximum Width				2.1
Width of Head / Width of Left Elytron				0.956
Pronotum: Width (at widest part) / Length				1.921
Length of Pronotum / Length of Head				1.306
ABL				4.544
**F.** *Hyboptera shasta* Erwin, sp. n.				
	**Males (N1)**		**Females (N0)**	
	**Range**	**Mean**	**Range**	**Mean**
Total Length (SBL)		3.885		
Maximum Width		2.174		
Width of Head / Width of Left Elytron		0.966		
Pronotum: Width (at widest part) / Length		1.873		
Length of Pronotum / Length of Head		1.361		
ABL		4.819		
**G.** *Hyboptera tepui* Erwin & Henry, sp. n.				
	**Males (N0)**		**Females (N1)**	
	**Range**	**Mean**	**Range**	**Mean**
Total Length (SBL)				4.289
Maximum Width				2.458
Width of Head / Width of Left Elytron				0.878
Pronotum: Width (at widest part) / Length				1.741
Length of Pronotum / Length of Head				1.309
ABL				5.155
**H.** *Hyboptera tiputini* Erwin & Henry, sp. n.				
	**Males (N15)**		**Females (N15)**	
	**Range**	**Mean**	**Range**	**Mean**
Total Length (SBL)	3.331–4.421	4.017	3.511–4.724	4.166
Maximum Width	2.008–2.924	2.363	1.868–2.902	2.432
Width of Head / Width of Left Elytron	0.796–0.987	0.897	0.819–1.017	0.899
Pronotum: Width (at widest part) / Length	1.699–2.076	1.823	1.688–1.936	1.812
Length of Pronotum / Length of Head	1.293–1.517	1.373	1.299–1.476	1.367
ABL	4.241–5.301	4.819	4.211–5.640	4.965
**I.** *Hyboptera viridivittis* Chaudoir				
	**Males (N3)**		**Females (N5)**	
	**Range**	**Mean**	**Range**	**Mean**
Total Length (SBL)	3.785–4.052	3.933	3.759–4.167	4.003
Maximum Width	2.34–2.458	2.399	2.184–2.586	2.374
Width of Head / Width of Left Elytron	0.841–0.892	0.871	0.842–0.922	0.893
Pronotum: Width (at widest part) / Length	1.729–1.853	1.798	1.765–1.930	1.84
Length of Pronotum / Length of Head	1.262–1.517	1.355	1.199–1.316	1.278
ABL	4.787–5.089	4.934	4.646–5.272	5.003
***tuberculata* species group**				
**J.** *Hyboptera auxilidora* Erwin				
	**Males (N6)**		**Females (N6)**	
	**Range**	**Mean**	**Range**	**Mean**
Total Length (SBL)	4.389–4.938	4.654	4.408–4.983	4.755
Maximum Width	2.42–3.28	2.77	2.524–2.922	2.726
Width of Head / Width of Left Elytron	0.727–0.980	0.88	0.875–0.936	0.906
Pronotum: Width (at widest part) / Length	1.774–1.988	1.881	1.743–2.019	1.868
Length of Pronotum / Length of Head	1.208–1.297	1.252	1.243–1.573	1.361
ABL	5.058–5.923	5.483	5.265–5.925	5.539
**K.** *Hyboptera dilutior* Oberthür				
	**Males (N15)**		**Females (N15)**	
	**Range**	**Mean**	**Range**	**Mean**
Total Length (SBL)	3.545–4.665	4.256	3.888–4.559	4.339
Maximum Width	1.952–2.772	2.416	2.708–3.208	3.024
Width of Head / Width of Left Elytron	0.909–1.012	0.959	0.743–0.850	0.786
Pronotum: Width (at widest part) / Length	1.709–1.925	1.811	1.701–2.003	1.827
Length of Pronotum / Length of Head	1.236–1.403	1.333	1.210–1.350	1.292
ABL	3.916–5.979	5.035	4.760–5.772	5.129
**L.** *Hyboptera lucida* Henry & Erwin, sp. n.				
	**Males (N5)**		**Females (N9)**	
	**Range**	**Mean**	**Range**	**Mean**
Total Length (SBL)	4.376–5.066	4.752	4.690–5.265	5.029
Maximum Width	2.362–3.218	2.849	2.820–3.248	3.054
Width of Head / Width of Left Elytron	0.845–0.976	0.887	0.828–0.957	0.883
Pronotum: Width (at widest part) / Length	1.713–1.924	1.84	1.742–1.981	1.88
Length of Pronotum / Length of Head	1.130–1.274	1.207	1.150–1.351	1.266
ABL	5.641–6.294	5.852	5.514–6.667	6.093
**M.** *Hyboptera tuberculata* (Dejean)				
	**Males (N15)**		**Females (N15)**	
	**Range**	**Mean**	**Range**	**Mean**
Total Length (SBL)	3.942–4.853	4.471	4.196–4.843	4.533
Maximum Width	2.178–2.928	2.609	2.206–3.116	2.689
Width of Head / Width of Left Elytron	0.819–1.058	0.899	0.839–1.029	0.893
Pronotum: Width (at widest part) / Length	1.731–2.223	1.876	1.726–1.993	1.859
Length of Pronotum / Length of Head	1.103–1.379	1.264	1.181–1.354	1.277
ABL	4.321–5.883	5.231	4.745–5.763	5.325
**N.** *Hyboptera verrucosa* (Reiche)				
	**Males (N14)**		**Females (N16)**	
	**Range**	**Mean**	**Range**	**Mean**
Total Length (SBL)	3.824–4.587	4.203	3.793–4.898	4.417
Maximum Width	2.060–2.636	2.352	2.190–2.846	2.478
Width of Head / Width of Left Elytron	0.893–1.025	0.954	0.862–1.024	0.928
Pronotum: Width (at widest part) / Length	1.774–1.997	1.882	1.797–2.012	1.865
Length of Pronotum / Length of Head	1.186–1.359	1.269	1.185–1.398	1.268
ABL	4.493–5.511	4.932	4.474–5.660	5.06
